# Comparative effects of pharmacological interventions in the prophylactic treatment of tension-type headache: systematic review and network meta-analysis

**DOI:** 10.1080/07853890.2026.2616972

**Published:** 2026-01-18

**Authors:** Qing-Feng Tao, Can Hua, Jian-Jiao Mou, Chao-Rong Xie, Zhen-Zhi Wang, Bo-Zhu Chen, Li Lin, Xin-Yu Li, Kulachai Pantila, Hui Zheng

**Affiliations:** a Acupuncture and Tuina School, Chengdu University of Traditional Chinese Medicine, Chengdu, China; b Department of Traditional Chinese Medicine, Dazhou Dachuan District People’s Hospital (Dazhou Third People’s Hospital), Dazhou, China

**Keywords:** Pharmacological interventions, tension-type headache, systematic review, network meta-analysis

## Abstract

**Background:**

Tension-type headache (TTH) is the most common neurological disorder. The comparative effect of pharmacological interventions for TTH prophylaxis remains unclear. We aimed to assess the comparative effects of pharmacological interventions in the prophylactic treatment of TTH.

**Methods:**

Ovid Medline, Embase, and Cochrane were searched from inception to 12 December, 2025. Randomized controlled trials (RCTs) of medications compared to placebo or another medication for preventing TTH were included. The primary outcome was headache days per month. A Bayesian random-effect model was employed as the primary analysis of chronic TTH.

**Results:**

Thirty-five RCTs were included, 33 (88.6%) RCTs involved chronic TTH patients, and 24 RCTs provided available data for meta-analysis. Amitriptyline 100 mg presented more reduction of monthly headache days than placebo at 4 and 8 weeks (4 weeks: MD −6.59, 95% CrI −11.22 to −0.64; 8 weeks: MD −6.14, 95% CrI −10.27 to −0.87). BTX-A 100 U can reduce monthly headache days (MD −3.79, 95% CrI −7.16 to −0.33). Amitriptyline 100 mg was the highest-ranked treatment for monthly headache days at 4 (SUCRA 0.85), 8 (SUCRA 0.85), and 24 (SUCRA 0.87) weeks; 12 weeks was lidocaine 25 ml (SUCRA 0.75). Amitriptyline 100 mg and BTX-A 500 U showed a higher adverse event rate than placebo.

**Conclusion:**

Amitriptyline 100 mg and BTX-A 100 U may be options to reduce monthly headache days in patients with chronic TTH. Given the low to very low certainty of evidence, high risk of bias, and high heterogeneity, more studies are needed.

**Trial registration:**

PROSPERO (CRD42025639586).

## HIGHLIGHTS

- There is a lack of a comprehensive review of the effectiveness of pharmacological interventions for the prophylactic treatment of tension-type headache (TTH).
- Low to very low certainty of evidence suggested that amitriptyline 100mg reduces headache days per month at 4 and 8 weeks, and it may also confer potential preventive benefits for patients with chronic TTH at 12 and 24 weeks.
- Amitriptyline 100mg and botulinum toxin type-A 500U showed a higher adverse event rate than placebo.

## Introduction

Tension-type headache (TTH) is a global health issue with 24.9% population suffering from this neurological disorder [1,2]. TTH is characterized by recurrent episodes of mild to moderate headache and accompanying bilateral pressing or tightening sensation [3]. The pathogenesis of TTH is complex [4] and often imposes a significant burden on patients, including reduced productivity, impaired quality of life, and financial strain [5–7].

To reduce the impact of TTH on patients, strengthening prevention is important [8]. Acute pharmacological interventions and lifestyle modifications are recommended for infrequent episodic TTH (less than 1 day per month), whereas preventive behavioral and/or pharmacological interventions are recommended for frequent episodic TTH (1–14 days per month) and chronic TTH (no less than 15 days per month) [3,9,10]. Currently, there are several pharmacological interventions that have been studied for the prevention of TTH [7,10]. Randomized controlled trials (RCTs) indicate that oral medications such as amitriptyline [11], mirtazapine [12], and tizanidine [13] can reduce the number of headache days per month. Meanwhile, injectable medications like botulinum toxin type A (BTX-A) [14] (typically injected into muscles with tension or headache sites such as the temporalis, frontalis, sternocleidomastoid, trapezius, semispinalis, and splenius capitis [14]) can also decrease the number of headache days per month. However, different RCTs have shown varying results [15,16]. In some studies, the effectiveness of nonsteroidal anti-inflammatory drugs such as ibuprofen [16], as well as the local anesthetic lidocaine [17], has been investigated for the prevention of TTH.

Currently, some systematic reviews have estimated the effectiveness of different pharmacological interventions in preventing TTH, but most studies have concentrated on a single method or a specific category of drugs [18–21]. Although a few have explored the effectiveness of multiple drugs simultaneously [22], the range of drugs included remains limited. Furthermore, previous studies have not distinguished the effects of different doses on the outcomes, data from different doses of a drug are often pooled together. This limits the applicability of the conclusions for guiding clinical practice. Furthermore, the existing guidelines also exhibit variability in their recommendations for prophylactic medications. The European Federation of Neurological Societies guideline suggests that amitriptyline 30–75 mg is the first choice, and mirtazapine 30 mg and venlafaxine 150 mg can be used as a second-line option [9]. While the US guideline recommends only amitriptyline 50 and 100 mg for preventive treatment [23]. Therefore, a comprehensive overview of existing literature to determine the comparative effects of different pharmacological interventions in preventing TTH is needed.

In this study, we aimed to estimate the comparative effects of different pharmacological interventions in the prophylactic treatment of TTH by systematic review and Bayesian-model network meta-analysis (NMA).

## Materials and methods

The systematic review and meta-analysis was conducted according to the Preferred Reporting Items for Systematic Reviews and Meta Analyses for Network Meta-Analysis (PRISMA-NMA) guideline [24]. The study was preregistered with PROSPERO (CRD42025639586).

## Literature search

We searched Ovid Medline, Embase, and Cochrane databases from inception to 17 January, 2025, and a supplementary search was conducted on 12 December, 2025 (QF-T), without any language restriction. Full search strategies are shown in Tables S1–S3. Meanwhile, we read the reference lists of previous systematic reviews to identify any potential studies.

## Inclusion and exclusion criteria

Eligibility criteria were designed according to the PICOS format: (1) Patients: adult patients with TTH diagnosed by the International Classification of Headache Disorders criteria; (2) Interventions: pharmacological intervention for TTH prophylaxis; (3) Comparisons: placebo or another pharmacological intervention; (4) Outcomes: headache days per month, headache intensity (the score that was measured by VAS or NRS), headache duration (headache hours per day), and adverse event; (5) Study type: RCT.

The study was excluded when it met one of the following criteria: (1) Non-pharmacological interventions; (2) Augmentation studies (intervention A + intervention B vs. intervention A); (3) Combination interventions; (4) Duplication.

## Study selection

After the removal of duplicates, two reviewers (QF-T and CH) screen the title and abstract for potentially eligible studies independently. Then, they read the full-text to identify the eligible studies. Disagreements were resolved by consensus or, if consensus could not be reached, by a third reviewer (HZ).

## Outcome assessments

The primary outcome was headache days per month. The secondary outcomes were headache intensity, headache duration, and adverse event rate. We assessed headache days per month, headache intensity, and headache duration at 4, 8, 12, and 24 weeks, and assessed the adverse event rate at the end of treatment.

## Data extraction

One reviewer (QF-T) extracted data and another (CH) verified it. The following data were extracted by using standard forms: characteristics of the study and population, details of pharmacological interventions and control arm, and outcome data. For a continuous outcome, we extracted the change in mean with SD between baseline and postintervention measures. For adverse events, we extracted the number of patients who experienced any adverse events.

## Assessment of study quality

We assessed the risk of bias of each included RCT by using the Cochrane Risk of Bias 2 tool [25]. Each RCT was assessed as low, some concerns, or high risk of bias. Meanwhile, we assessed the certainty of evidence regarding each outcome by using the Confidence in Network Meta-Analysis (CINeMA) framework [26]. In CINeMA, reporting bias is assessed using the ROB-MEN tool [27]. Evidence of each pairwise comparison was assessed as high, moderate, low, or very low. Quality assessments were conducted by two independent reviewers (JJ-M and CR-X).

## Statistical analysis

The main analysis of Bayesian NMA was conducted by using the *multinma* package in R software version 4.3.1 [28]. We chose the vague priors to conduct the Bayesian framework. The prior distributions for the treatment effects and study-specific intercepts were set as N (0.100^2^), and the prior for the heterogeneity standard deviation was set as half-N (5^2^) of random-effect model. We evaluated the model fit by calculating the posterior total residual deviance, the number of unconstrained data points, and the deviance information criteria (DIC) of random-effect model and the fixed-effect model. The posterior total residual deviance is closer to the number of unconstrained data points, and with a lower DIC suggests a better model fit. We draw a dev-dev plot to assess the global inconsistency. If the points of the consistency model and the inconsistency model approximately lie on the equal line, it suggests there was no evidence of inconsistency. The effect size of continuous outcomes was pooled by mean difference (MD) with 95% credible intervals (CrIs), and adverse event rate was pooled by odds ratio (OR) with 95% CrIs. We calculated the heterogeneity among studies by tau-squared (τ^2^), and τ^2^ over 0.36 suggesting a significant heterogeneity [29]. We calculated the surface under the cumulative rank curve (SUCRA) value to estimate the ranking of interventions in the network, and an intervention with values nearest 1 suggests a preferred intervention [30,31].

To assess the robustness of results, we conducted three sensitivity analyses of the primary outcome: excluding RCTs at high risk of bias, excluding RCTs with less than 50 participants, and pooling data by using a frequentist approach. The frequentist analysis was conducted by using *netmeta* package.

At the same time, since 88.6% of the studies available for quantitative analysis focused on chronic tension-type headaches. Therefore, we performed the primary analysis in chronic TTH. Because frequent episodic TTH often warrants prophylactic treatment yet few RCTs report pharmacological prophylaxis separately for this subgroup, we combined headache days per month data—the primary outcome—from both episodic and chronic TTH as an exploratory analysis.

## Results

### Characteristics of the included RCTs

**Figure 1.**
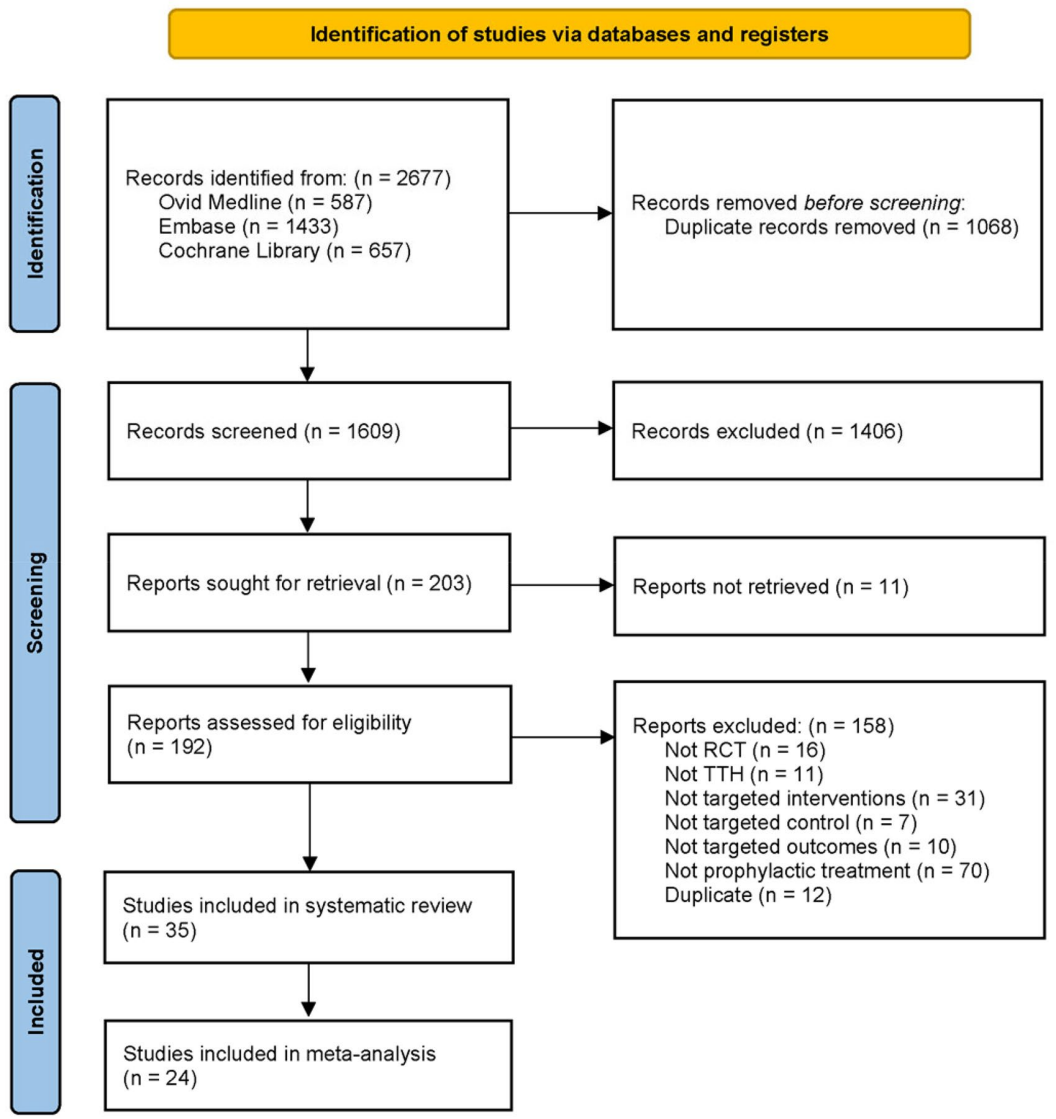
Study flow diagram.

Our search identified 2677 records. After de-duplication, 1609 records were screened in title and abstract and then 203 records were assessed for eligibility. Following full-text screening (Table S4), 35 articles (2795 participants) were included in systematic review [11–17,32–59], in which 24 articles (2006 participants) provided available data for meta-analysis [11,12,14–17,32–34,36–38,42–46,48–50,54,55,57,58]. The review process is shown in Figure 1.

The characteristics of the included RCTs are presented in Table 1. The included RCTs were conducted in 18 countries. The mean age of participants was 41.97 years, and 66% of participants were female. 33 (88.6%) RCTs involved chronic TTH patients, 1 (2.9%) RCT involved episodic TTH patients, and 3 (8.5%) RCTs involved both chronic and episodic TTH patients. The mean sample size was 80 participants and ranged between 13 and 300 participants. The included RCTs involved 23 different drugs. Ten RCTs included amitriptyline (titration: eight; fixed-dose: two), ten RCTs included BTX-A, three RCTs included mitazapine, two RCTs included fluoxetine, two RCTs included tizanidine, two RCTs included lidocaine, two RCTs included flunarizine, and one each included sertraline, buspirone, citalopram, amitriptylinoxide, desipramine, fluvoxamine, gabapentin, L-5-Hydroxytryptophan, depomedrol, mianserine, memantine, ibuprofen, paroxetine, sulpiride, sodium valproate, and venlafaxine.

**Table 1.** Characteristics of the included RCTs.

Study	Country	Diagnostic criteria	Type of TTH	Interventions	Treatment duration (weeks)	Follow-up duration (weeks)	Number of patients	Female (%)	Mean ages
Asadi 2012 [51]	Iran	ICHD-II	CTTH	Gabapentin 600 mg vs. Depomedrol 40 mg	8	0	278	65	NA
Bendtsen 2004 [12]	Denmark	ICHD-I	CTTH	Mirtazapine 30 mg vs. Placebo	8	0	24	45	45
Bendtsen 2007 [16]	Denmark	ICHD-I	CTTH	Mirtazapine 4.5 mg vs. Ibuprofen 400 mg vs. Placebo	8	0	93	50	39.25
Bendtsen 1996 [32]	Denmark	ICHD-I	CTTH	Amitriptyline 75 mg (titration) vs. Citalopram 20 mg vs. Placebo	8	0	40	63	40
Boz 2003 [52]	Turkey	ICHD-I	CTTH	Amitriptyline 25 mg (titration) vs. Sertraline 50 mg	12	0	90	88	39.1
Fogelholm 1992 [13]	Finland	ICHD-I	CTTH	Tizanidine 6 mg vs. Placebo	6	0	45	100	37
Göbel 1994 [41]	Germany	ICHD-I	CTTH	Amitriptyline 75 mg (titration) vs. Placebo	6	0	78	69	87
Hamdy 2009 [14]	Egypt	ICHD-II	CTTH	BTX-A 50 U vs. Placebo	one session	3	28	68	36.57
Harden 2009 [36]	USA	ICHD-II	CTTH	BTX-A 100 U vs. Placebo	one session	12	23	52	45.4
Holroyd 2001 [11]	USA	ICHD-I	CTTH	Amitriptyline 100 mg (titration) vs. Placebo	8	24	203	76	37
Karadaş 2013a [46]	Turkey	ICHD-II	ETTH	Lidocaine 2 ml vs. Lidocaine 10 ml vs. Placebo	2–5 sessions	24	108	70	36.28
Karadaş 2013b [17]	Turkey	ICHD-II	CTTH	Lidocaine 25 ml vs. Placebo	9 days	12	48	81	40.55
Kokoska 2004 [37]	USA	ICHD-I	CTTH	BTX-A 50 U vs. Placebo	one session	24	40	78	46.45
Langemark 1994 [53]	Denmark	ICHD-I	CTTH	Paroxetine 30 mg vs. Sulpiride 400 mg	8	0	50	60	42
Lindelof 2009 [47]	Denmark	ICHD-II	CTTH	Memantine 40 mg vs. Placebo	10	0	40	58	39
Manna 1994[40]	Italy	ICHD-I	CTTH	Fluvoxamine 100 mg vs. Mianserine 60 mg	8	0	40	63	36.2
Martín-Araguz 2003 [56]	Spain	ICHD-I	CTTH	Amitriptyline 25 mg (fixed-dose) vs. Mirtazapine 30 mg	24	0	60	70	38.4
Mitsikostas 1997 [39]	Greece	ICHD-I	CTTH	Amitriptyline 50 mg (titration) vs. Buspirone 30 mg	12	0	58	62	42.5
Murros 2000 [48]	Finland	ICHD-I	CTTH	Tizanidine 6 mg vs. Tizanidine 12 mg vs. Placebo	6	0	185	75	44
Oguzhanoglu 1999 [59]	Turkey	ICHD-I	CTTH, ETTH	Amitriptyline 50 mg (titration) vs. Fluoxetine 20 mg	4	0	13	12	38.5
Padberg 2004 [15]	Netherland	ICHD-I	CTTH	BTX-A 100 U vs. Placebo	one session	12	40	70	44.5
Pfaffenrath 1994 [44]	Germany, Austria, Switzerland	ICHD-I	CTTH	Amitriptyline 75 mg (titration) vs. Amitriptylinoxide 90 mg (titration) vs. Placebo	12	8	197	56	38
Ribeiro 2000 [45]	Portugal	ICHD-I	CTTH	L-5-Hydroxytryptophan 100 mg vs. Placebo	8	2	78	90	39.8
Rollnik 2000 [58]	Germany	ICHD-I	CTTH, ETTH	BTX-A 200 U vs. Placebo	one session	12	21	62	37.4
Schmitt 2001 [42]	Switzerland	ICHD-I	CTTH	BTX-A 20 U vs. Placebo	one session	8	60	60	46.4
Schulte-Mattler 2004 [57]	Germany	ICHD-I	CTTH	BTX-A 500 U vs. Placebo	one session	12	112	46	45.5
Silberstein 2006 [38]	USA	ICHD-I	CTTH	BTX-A 50 U vs. BTX-A 86 U vs. BTX-A 100 U vs. BTX-A 150 U vs. Placebo	one session	120day	300	62	42.6
Smuts 1999 [50]	South Africa	ICHD-I	CTTH	BTX-A 100 U vs. Placebo	one session	12	41	73	n
Straube 2008 [49]	Germany	ICHD-I	CTTH	BTX-A 210 U vs. BTX-A 420 U vs. Placebo	one session	12	125	53	41.7
Surbakti 2017 [43]	Indonesia	ICHD-II	CTTH	Amitriptyline 12.5 mg (fixed-dose) vs. Flunarizine 5 mg vs. Flunarizine 10 mg	2	0	95	82	44.6
Surbakti 2021 [54]	Indonesia	ICHD-II	CTTH	Flunarizine 5 mg vs. Flunarizine 10 mg vs. Placebo	8	0	24	84	44.5
Vernon 2009 [34]	Canada	ICHD-II	CTTH	Amitriptyline 25 mg (titration) vs. Placebo	14	12	20	80	33.9
Walker 1998 [35]	UK	ICHD-I	CTTH	Desipramine 75 mg vs. Fluoxetine 20 mg	4	8	37	81	35
Yurekli 2008 [55]	Turkey	ICHD-I	CTTH	Sodium valproate 1 g vs. Placebo	12	0	41	NA	40.2
Zissis 2007 [33]	Greece	ICHD-I	CTTH, ETTH	Venlafaxine 150 mg vs. Placebo	12	0	60	49	40.8

RCT: randomized controlled trial; TTH: tension-type headache; CTTH: chronic tension-type headache; ETTH: episodic tension-type headache.

The details of risk of bias assessment are illustrated in Figure S1. Eight (22.9%) RCTs were identified as low risk, eighteen (51.4%) RCTs with some concerns, and nine (25.7%) RCTs were high risk.

The results of total posterior residuals deviance, the number of data points, and DIC for both random-effect and fixed-effect models are shown in Table S5. Most of the random-effect models had a posterior total residual deviance that was closer to the point estimates. Considering the small difference in DIC between random-effect and fixed-effect models, and there is a large heterogeneity between studies. Therefore, we ultimately chose the random-effect models. The results of the consistency assessment are illustrated in Figure S2–S5, which suggests there is no evidence of inconsistency. Therefore, we used the random-effect consistency model to conduct meta-analysis.

### Headache days per month

**Figure 2.**
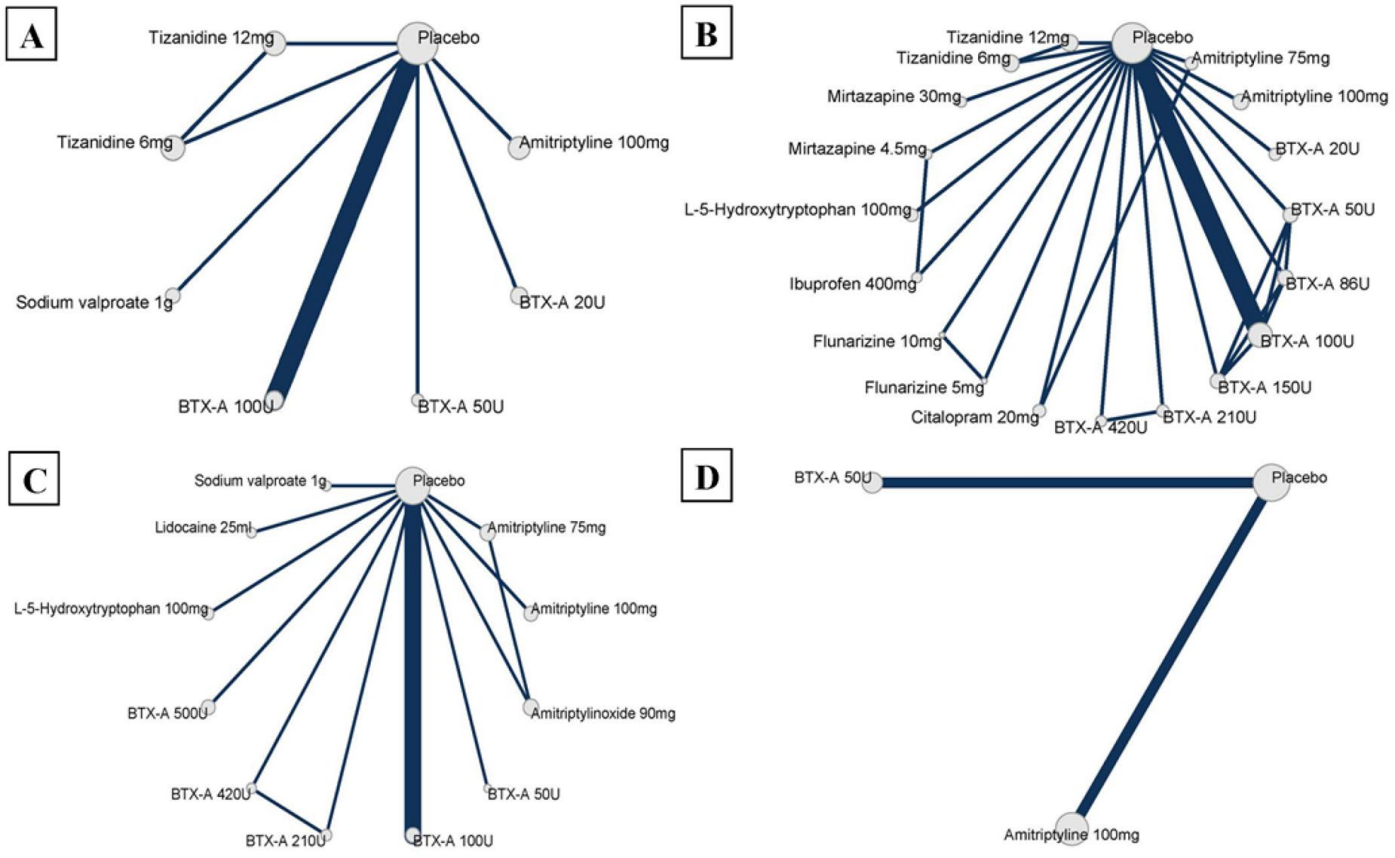
Network diagram of comparison of headache days per month. (A) 4 weeks; (B) 8 weeks; (C) 12 weeks; (D) 24 weeks. The size of the grey nodes represents the number of included participants, and the thickness of the line represents the number of included studies.

**Figure 3.**
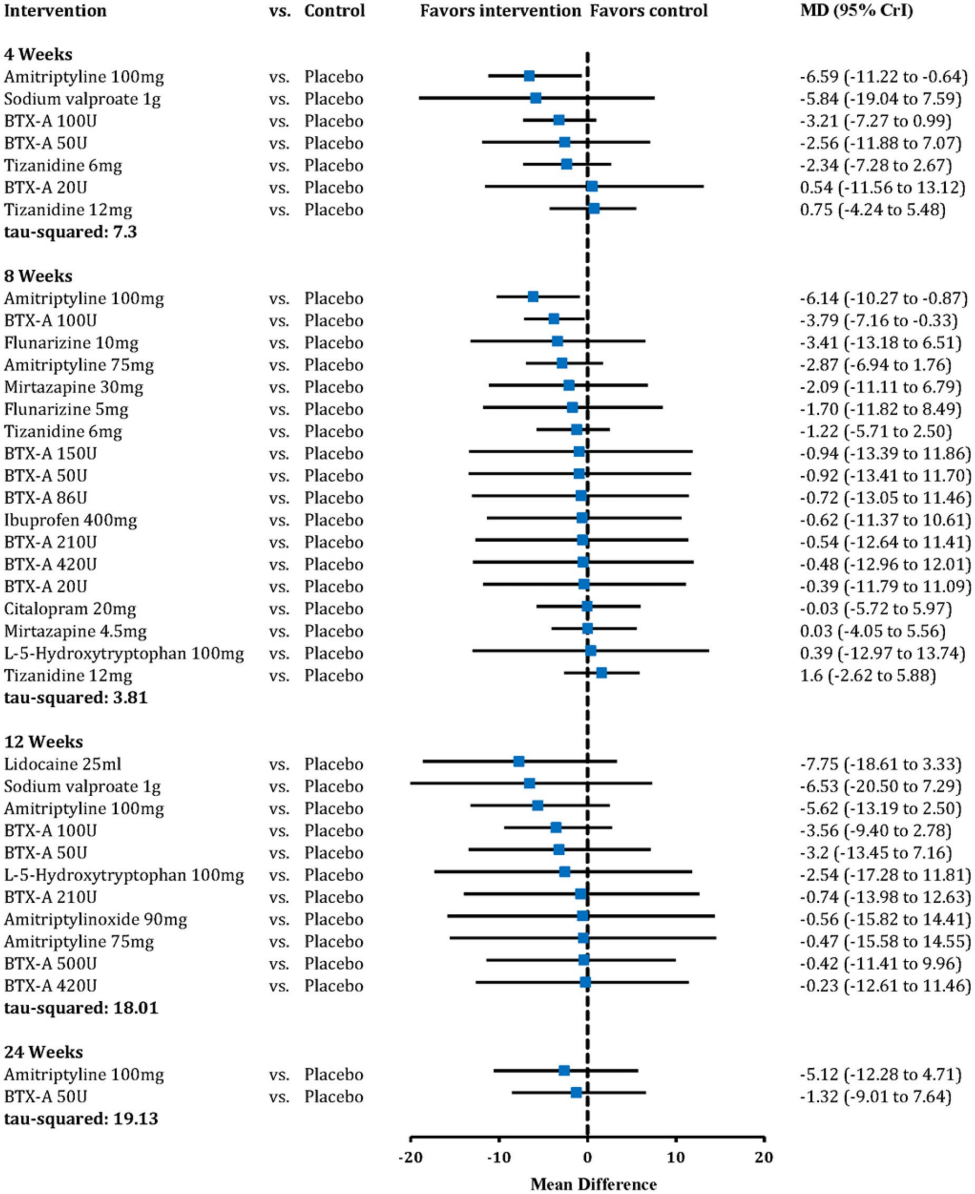
Effect size of pharmacological interventions vs. Placebo of headache days per month. BTX-A: botulinum toxin type-A; MD: mean difference; CrI: credible interval.

The details of the studies included at different data points of headache days per month are shown in Table 2 and Figure 2. Amitriptyline 100 mg was associated with a mean reduction of 6.59 headache days per month (95% CrI −11.22 to −0.64) relative to placebo at 4 weeks, and 6.14 days (95% CrI −10.27 to −0.87) at 8 weeks with low-certainty evidence (Figure 3 and Table S6). At 12 and 24 weeks, amitriptyline 100 mg showed a trend toward reduction in headache days, but the 95% CrI was wide and crossed zero with very low certainty of evidence (12 weeks: MD −5.62, 95% CrI −13.19 to 2.50; 24 weeks: MD −5.12, 95% CrI −12.28 to 4.71, Figure 3 and Table S6). BTX-A 100 U showed a better effect size than placebo at 8 weeks with very low certainty of evidence (MD −3.79, 95% CrI −7.16 to −0.33, Figure 3 and Table S6). Pair-wise comparison suggested amitriptyline 100 mg had a better effect size than tizanidine 12 mg (MD −7.74, 95% CrI 1.43 to 13.40, Table S8) at 8 weeks. All other comparisons showed wide 95% CrI that included zero, and the certainty of evidence were moderate to very low (Figure 3 and Tables S6–S10).

**Table 2.** Details of NMA.

Outcomes	Data points	Number of RCTs	Number of participants
Headache days per month	4 weeks	7[11, 14, 36, 42, 48, 50, 55]	474
	8 weeks	12[11, 12, 16, 32, 36, 38, 42, 45, 48–50, 54]	1113
	12 weeks	10[11, 14, 15, 36, 44, 45, 49, 50, 55, 57]	805
	24 weeks	2[11, 37]	141
Headache intensity	4 weeks	7[11, 14, 36, 42, 48, 50, 55]	474
	8 weeks	10[11, 12, 16, 32, 36, 38, 42, 45, 48, 50]	978
	12 weeks	9[11, 14, 15, 36, 44, 45, 49, 50, 55]	698
	24 weeks	2[11, 37]	132
Headache duration	4 weeks	3[14, 48, 49]	331
	8 weeks	5[12, 16, 32, 48, 49]	511
	12 weeks	4[14, 15, 44, 49]	383
Adverse event rate	NA	17[11, 12, 14–16, 32, 34, 36–38, 42, 44–46, 49, 55, 57]	1500

NMA: network meta-analysis; RCT: randomized controlled trial.

The SUCRA showed amitriptyline 100 mg ranked first at 4, 8, and 12 weeks (SUCRA: 4 weeks 0.85, 8 weeks 0.85, 12 weeks 0.87, Table S11) and lidocaine 25 ml ranked first at 12 weeks (SUCRA 0.75, Table S11).

### Headache intensity

The details of the studies included at different data points of headache intensity are shown in Table 2 and Figure S6. Compared to placebo, amitriptyline 100 mg, BTX-A 50 U, BTX-A 100 U, sodium valproate 1 g, L-5-Hydroxytryptophan 100 mg, and lidocaine 25 ml showed a potential reduction in headache intensity, but the credible intervals for all pharmacological interventions crossed the null line (Figure S7). Pair-wise comparison suggested there was a similar effect between interventions (Figure S6, Tables S12–S15). The certainty of evidence was moderate to very low (Table S6).

The SUCRA suggested that sodium valproate 1 g (SUCRA 0.60), BTX-A 100 U (SUCRA 0.64), lidocaine 25 ml (SUCRA 0.87), and amitriptyline 100 mg (SUCRA 0.62) were ranked first at 4, 8, 12, and 24 weeks, respectively (Table S11).

### Headache duration

The details of the studies included at different data points of headache duration are shown in Table 2 and Figure S8. Our analysis showed that pharmacological interventions had a similar effect compared to placebo in reducing headache duration (Figure S9). There was a similar effect between the pharmacological interventions’ comparisons (Figure S8, Tables S16–S18). The certainty of evidence was moderate to very low (Table S6). The SUCRA revealed that BTX-A 50 U (SUCRA 0.56), amitriptyline 75 mg (SUCRA 0.58), and BTX-A 420 U (SUCRA 0.53) were ranked first at 4, 8, and 12 weeks, respectively (Table S11).

### Adverse event rate

The details of the studies included at different data points of the adverse event rate are shown in Table 2 and Figure S10. Low-certainty evidence suggested that amitriptyline 100 mg and BTX-A 500 U were associated with a higher adverse event rate compared to placebo (amitriptyline 100 mg: OR 9.53, 95% CrI 1.15 to 15.63; BTX-A 500 U: OR 19.04, 95% CrI 1.20 to 45.76; Figure S11, Table S6 and S19). The main adverse events associated with amitriptyline 100 mg were drowsiness and dry mouth. The main adverse event of BTX-A 500 mg was transient weakness of the eyelids and the neck. The SUCRA demonstrated that BTX-A 500 U ranked last with a value of 0.14 (Table S11).

### Sensitivity analysis

We conducted three sensitivity analyses of the primary outcome to evaluate the robustness of the results. The results of sensitivity analyses are shown in Figures S12–S14. The Bayesian analyses of excluding RCTs at high risk of bias and excluding RCTs with less than 50 participants showed that the MDs have slight differences compared with the primary analysis, but the results were stable overall (Figures S12 and 13). However, the results of the frequentist approach suggested that sodium valproate 1 g, BTX-A 50 U, BTX-A 100 U, tizanidine 6 mg, lidocaine 25 ml, and amitriptyline 75 mg might also have a significant positive effect on chronic TTH (Figure S14).

### Exploratory analysis

We conducted an exploratory analysis by combining data from prophylactic treatment for episodic and chronic TTH of headache days per month, since frequent episodic TTH also needed preventive treatment in the clinic. Compared with the primary analysis, effect estimates for reducing headache days per month changed only slightly and remained broadly consistent. Amitriptyline 100 mg yielded greater reductions than placebo at 4 and 8 weeks; at 12 and 24 weeks, the effect still favored amitriptyline 100 mg, but with a wide credible interval (Figure S15).

## Discussion

In this study, we conducted a comprehensive review of pharmacological interventions in the prophylactic treatment of TTH. A total of 35 RCTs were included, and 24 RCTs provided data for performing NMA. Our findings showed that amitriptyline 100 mg was associated with a greater reduction in headache days per month than placebo at 4 and 8 weeks, and potential benefits were also observed at 12 and 24 weeks. In terms of reducing headache intensity, amitriptyline 100 mg, BTX-A 50 U, BTX-A 100 U, sodium valproate 1 g, L-5-Hydroxytryptophan 100 mg, and lidocaine 25 ml showed a potential reduction in the intensity of headache. For reducing headache duration, we found that no pharmacological intervention was superior to placebo. Amitriptyline 100 mg and BTX-A 500 U were found to lead to a higher adverse event rate than placebo.

Amitriptyline, a tricyclic antidepressant, has been recommended as a first-line treatment for the prevention of TTH in guidelines [9,23]. Previous systematic reviews have shown that amitriptyline is significantly more effective than placebo in reducing headache days per month [20,21], which is consistent with our findings. The prophylactic treatment of TTH with amitriptyline typically involves a titration approach, starting with a low dose and gradually adjusting to the optimal dose [11]. However, the previous studies pooled different doses of amitriptyline without distinguishing the effects of different doses on outcomes [20,21]. Compared to previous studies, we differentiated the doses of amitriptyline. Our study found low-quality evidence that amitriptyline 100 mg reduced headache days per month at 4 weeks (−6.59 days) and 8 weeks (−6.14 days). By 12 and 24 weeks, a preventive effect remained plausible (12 weeks: −3.56 days; 24 weeks: −5.12 days). The effect size of amitriptyline 100 mg exceeded the minimal clinically important difference (MCID), defined as a reduction of at least one day per month [18]. Also, there was a favorable trend for amitriptyline 75 mg (8 weeks: −2.87 days), but the quality of evidence was low, and the credible intervals were wide. Therefore, more studies are needed to assess the preventive effect of amitriptyline, especially the long-term effect.

Considering the potential efficacy of amitriptyline in reducing headache days per month and headache intensity and its potential for significant adverse events, shared decision-making between patients and physicians is essential in clinical practice. A patient-centered approach should be adopted to select the most appropriate preventive treatment, followed by regular monitoring of response and timely strategy adjustments [7].

BTX-A is a muscle contraction inhibitor that works by inhibiting the release of acetylcholine [60]. Evidence from previous studies remains controversial regarding the effectiveness of BTX-A for preventing TTH. It was shown to be superior to placebo in some studies [18,61], but did not show significant effectiveness in others [19]. In the latest US guidelines, BTX-A is listed as ‘weak against’ [23]. Our study showed that BTX-A 100 U presented a greater reduction of headache days per month than placebo at 8 weeks (MD −3.79), and the effect size exceeded the MCID. At 4 weeks and 12 weeks, BTX-A 100 U also exceeded the MCID, but its credible interval was wide, and the quality of evidence was low. Also, sensitivity analyses using frequentist methods showed that BTX-A 100 U achieved a statistically significant reduction in monthly headache days relative to placebo at both 4 and 8 weeks, whereas BTX-A 50 U showed a significant benefit at 4 weeks. Furthermore, the adverse event rate of BTX-A 500 U was higher than placebo. The effectiveness of BTX-A in the treatment of TTH can be explored by more studies.

Acute analgesics and nonsteroidal anti-inflammatory drugs, such as ibuprofen and acetaminophen, are commonly used for symptomatic relief of an acute episode of TTH [9,23,62]. One study has explored the prophylactic effects of ibuprofen on TTH [16]. In our study, ibuprofen showed a similar prophylactic effect compared to placebo. Additionally, overusing acute medications would lead to medication overuse headache [63], and reducing the overuse of acute medications is an important aspect of headache management. Hence, acute analgesics should be used with caution to avoid medication-overuse headache in patients with chronic TTH.

For other pharmacological interventions, such as sodium valproate 1 g, tizanidine 6 mg, mirtazapine 30 mg, L-5-Hydroxytryptophan 100 mg, lidocaine 25 ml, the 95% CrIs crossed the null value. Nevertheless, because their effect sizes reached the MCID and showed a significantly better effect than placebo, these interventions remain noteworthy for reducing the number of days and the intensity of chronic TTH.

In our study, one phenomenon warrants discussion: Bayesian and frequentist estimates may differ, with Bayesian analyses typically yielding wider credible intervals than their frequentist counterparts. This discrepancy likely stems from differences between the Bayesian and frequentist theoretical frameworks. The Bayesian NMA employed weakly informative priors that shrink small-study effects toward the null, whereas the frequentist approach gives greater weight to small trials with positive findings [64]. For this reason, and due to the small sample size included, some intervention effect sizes may have reached the MCID threshold while their credible interval still exceeded the null line, underscoring the need for cautious interpretation of the results.

Our study has several limitations. At study and outcome level: First, there was high heterogeneity among the included RCTs, which may undermine the reliability of pooled estimates. The source of heterogeneity may result from the differences in interventions and included populations, as well as the underpowered and/or poor methodological quality of studies with small samples. Therefore, we conducted a sensitivity analysis by excluding RCTs with less than 50 participants, but this did not significantly reduce heterogeneity. A systematic review of migraine excluded RCTs with less than 100 participants [65], which might reduce the impact of small sample sizes effects. However, due to the relatively small number of studies of TTH compared to migraine, excluding RCTs with less than 100 participants would exclude most RCTs from our analysis, and the analysis would not be performed. Therefore, given that most included studies had small sample sizes—potentially contributing to heterogeneity—our findings should be interpreted with caution. Second, we aimed to compare the prophylactic effects of different pharmacological interventions for TTH. However, due to the limited number of head-to-head RCTs that compared different pharmacological interventions, so our results were largely derived from indirect comparisons using placebo as a reference. While placebo effects may vary between different types of placebos, invasive placebo might have a stronger effect than oral placebo [66]. Therefore, it is important to note that combining different placebo interventions may lead to bias in our results, which could overestimate the efficacy of injectable treatments (such as BTX-A and lidocaine) and inflate their SUCRA scores. Third, among the included RCTs, only eight (22.9%) were rated as low risk of bias, while nine (25.7%) were rated as high risk of bias. The main reasons for downgrading were randomization process, deviations from intended interventions, and missing outcome data. Although we conducted a sensitivity analysis of the primary outcome by excluding these studies and found that the results remained stable overall, the high or unclear risk of bias in the included RCTs should be noted, potentially undermining the reliability of the pooled estimates.

At review level: First, when extracting data, we use a process in which one author performs the extraction and another verifies it. This approach still carries a risk of error. Second, we extracted only the overall incidence of adverse events reported in randomized controlled trials, without providing data stratified by severity. Consequently, our safety analysis cannot distinguish between mild, self-limiting symptoms and serious or treatment-limiting events, thereby limiting the clinical interpretability of the adverse event characteristics. Third, although we reported results up to 24 weeks, due to the limited number of included studies, low-quality evidence, and high risk of bias, further research is needed to validate the long-term effects of pharmacological interventions for preventing TTH. Fourth, non-pharmacological strategies were outside the scope of the review, such as vitamins, supplements, or manual or integrative therapies such as acupuncture. Also, some RCTs have explored the effectiveness of amitriptyline 12.5 mg [43], 25 mg [56], and 50 mg [39,59] for preventing TTH, but these studies were not included in our quantitative analysis due to the lack of available data or appropriate control measures. This is another limitation of our study.

## Conclusion

In this comprehensive systematic review and NMA that included 35 RCTs, we found low to very low certainty evidence suggested that amitriptyline 100 mg reduces the number of headache days per month at 4 and 8 weeks, and it may also confer potential preventive benefits for chronic TTH at 12 and 24 weeks. Meanwhile, BTX-A 100 U, BTX-A 50 U, sodium valproate 1 g, tizanidine 6 mg, lidocaine 25 ml, and amitriptyline 75 mg show considerable potential for preventing chronic TTH. Considering the low to very low certainty of evidence and the high heterogeneity, more high-quality studies are warranted.

## Supplementary Material

PRISMA checklist.docx

Supplementary materials.docx

## Data Availability

The data that supports our study are shown in the article and supplementary material; further inquiries can be directed to the corresponding author.
