# Supplementary material for: Comparative effects of pharmacological interventions in the prophylactic treatment of tension-type headache: systematic review and network meta-analysis
Source: Ann Med. 2026 Jan 18;58(1):2616972. doi: 10.1080/07853890.2026.2616972 (PMC12818289; doi:10.1080/07853890.2026.2616972)
Supplement: Supplementary materials.docx [file IANN_A_2616972_SM5552.docx]

**Supplementary materials files**

[Table S1. Search strategy in Ovid Medline 3](#_Toc218625663)

[Table S2. Search strategy in Embase 4](#_Toc218625664)

[Table S3. Search strategy in Cochrane Library 5](#_Toc218625665)

[Table S4. The reference list of the articles excluded at full text phase 6](#_Toc218625666)

[Table S5. Summary of fixed-effects and random-effects model fit statistics from network meta-analysis 21](#_Toc218625667)

[Table S6. Certainty of evidence of outcomes 24](#_Toc218625668)

[Table S7. The relative effects between different interventions for headache days per month at 4 weeks 57](#_Toc218625669)

[Table S8. The relative effects between different interventions for headache days per month at 8 weeks 59](#_Toc218625670)

[Table S9. The relative effects between different interventions for headache days per month at 12 weeks 67](#_Toc218625671)

[Table S10. The relative effects between different interventions for headache days per month at 24 weeks 71](#_Toc218625672)

[Table S11. Value of SUCRA for each treatment on outcomes 72](#_Toc218625673)

[Table S12. The relative effects between different interventions for headache intensity at 4 weeks 74](#_Toc218625674)

[Table S13. The relative effects between different interventions for headache intensity at 8 weeks 76](#_Toc218625675)

[Table S14. The relative effects between different interventions for headache intensity at 12 weeks 81](#_Toc218625676)

[Table S15. The relative effects between different interventions for headache intensity at 24 weeks 84](#_Toc218625677)

[Table S16. The relative effects between different interventions for headache duration at 4 weeks 85](#_Toc218625678)

[Table S17. The relative effects between different interventions for headache duration at 8 weeks 86](#_Toc218625679)

[Table S18. The relative effects between different interventions for headache duration at 12 weeks 89](#_Toc218625680)

[Table S19. The relative effects between different interventions for adverse event rate 90](#_Toc218625681)

[Figure S1. Risk of bias of included RCTs 99](#_Toc218625682)

[Figure S2. The “dev-dev” plots of random-effects consistency and inconsistency models for NMA of headache days per month 100](#_Toc218625683)

[Figure S3. The “dev-dev” plots of random-effects consistency and inconsistency models for NMA of headache intensity 101](#_Toc218625684)

[Figure S4. The “dev-dev” plots of random-effects consistency and inconsistency models for NMA of headache duration 102](#_Toc218625685)

[Figure S5. The “dev-dev” plots of random-effects consistency and inconsistency models for NMA of adverse event rate 103](#_Toc218625686)

[Figure S6. Network diagram of comparison of headache intensity 104](#_Toc218625687)

[Figure S7. Effect size of pharmacological interventions vs. Placebo of headache intensity 105](#_Toc218625688)

[Figure S8. Network diagram of comparison of headache duration 106](#_Toc218625689)

[Figure S9. Effect size of pharmacological interventions vs. Placebo of headache duration 107](#_Toc218625690)

[Figure S10. Network diagram of comparison of adverse event rate 108](#_Toc218625691)

[Figure S11. Effect size of pharmacological interventions vs. placebo of adverse event rate 109](#_Toc218625692)

[Figure S12. Sensitivity analysis of headache days per month after excluding RCTs at high risk of bias 110](#_Toc218625693)

[Figure S13. Sensitivity analysis of headache days per month after excluding RCTs less than 50 participants 111](#_Toc218625694)

[Figure S14. Sensitivity analysis of headache days per month using a frequentist approach 112](#_Toc218625695)

[Figure S15. Exploratory analysis of headache days per month by combining data from episodic and chronic tension-type headache 113](#_Toc218625696)

Table S1. Search strategy in Ovid Medline

| **ID** | **Search strategy** |
| --- | --- |
| 1 | randomized controlled trial.pt. |
| 2 | controlled clinical trial.pt. |
| 3 | randomized.ab,ti. |
| 4 | randomised.ab,ti. |
| 5 | randomly.ab,ti. |
| 6 | 1 or 2 or 3 or 4 or 5 |
| 7 | limit 6 to humans |
| 8 | exp tension-type headache/ |
| 9 | tension-type headache.ab,ti. |
| 10 | 8 or 9 |
| 11 | 7 and 10 |

Table S2. Search strategy in Embase

| **ID** | **Search strategy** |
| --- | --- |
| 1 | 'randomized controlled trial'/exp |
| 2 | 'controlled clinical trial'/exp |
| 3 | randomized:ab,ti |
| 4 | randomised:ab,ti |
| 5 | randomly:ab,ti |
| 6 | #1 OR #2 OR #3 OR #4 OR #5 |
| 7 | (#1 OR #2 OR #3 OR #4 OR #5) AND [humans]/lim |
| 8 | 'tension-type headache'/exp |
| 9 | 'tension-type headache':ab,ti |
| 10 | #8 OR #9 |
| 11 | #7 AND #10 |

Table S3. Search strategy in Cochrane Library

| **ID** | **Search strategy** |
| --- | --- |
| 1 | (randomized controlled trial):pt |
| 2 | (controlled clinical trial):pt |
| 3 | (randomized):ti,ab,kw |
| 4 | (randomised):ti,ab,kw |
| 5 | (randomly):ti,ab,kw |
| 6 | #1 OR #2 OR #3 OR #4 OR #5 in Trials |
| 7 | MeSH descriptor: [Tension-Type Headache] explode all trees |
| 8 | (tension-type headache):ti,ab,kw |
| 9 | #7 OR #8 |
| 10 | #6 AND #9 |

Table S4. The reference list of the articles excluded at full text phase

| Title | Author, year | Reason of excluded |
| --- | --- | --- |
| A differential response to treatment with divalproex sodium in patients with intractable headache | Rothrock, 1994 | Not RCT |
| Amitriptyline is effective in chronic but not in episodic tension-type headache: Pathogenetic implications | Cerbo,1998 | Not RCT |
| Botulinum A toxin for wrinkles: Release from tension headache | Wollina,2000 | Not RCT |
| Botulinum toxin therapy of migraine and tension-type headache: Comparing different botulinum toxin preparations | Schulte-Mattler,2006 | Not RCT |
| Chronic tension-type headache | Goadsby,2002 | Not RCT |
| Is any one analgesic superior for episodic tension-type headache? | Verhagen,2006 | Not RCT |
| IV metoclopramide Vs IV ketorolac in the treatment of acute primary headaches: A randomized clinical trial | Chowdhury,2022 | Not RCT |
| Memantine for the prophylaxis of chronic tension-type headache. | Henry,2009 | Not RCT |
| Nitric oxide synthase inhibitors for the treatment of chronic tension-type headache | Ashina,2002 | Not RCT |
| Possibilities of preventive therapy in frequent episodic tension-type headache | Tabeeva,2016 | Not RCT |
| Spinal manipulation vs. amitriptyline for the treatment of chronic tension headache: a randomized clinical trial | Hobson,1996 | Not RCT |
| Tension headache: Botulinum toxin is not effective | Diener,2008 | Not RCT |
| The effects of melatonin treatment on headache and vasomotor reactivity in patients with chronic tension-type headache | Eroglu,2021 | Not RCT |
| To use or not to use dipyrone? | Bigal,2002 | Not RCT |
| Treatment of Primary Headache Disorders with Intravenous Valproate: Initial Outpatient Experience | Stillman,2004 | Not RCT |
| Trials on chronic tension-type headache: using fair outcome measures | Al Bedah,2016 | Not RCT |
| A novel formulation of ibuprofen sodium has a safety profile comparable to standard ibuprofen tablets: A pooled analysis of randomized clinical trials | Kellstein,2013 | Not TTH |
| Big data application utilizing large patient group having chronic migraine and chronic tension-type headache: a health informatics study | Hu,2017 | Not TTH |
| Chronic daily headache prophylaxis with tizanidine: A double-blind, placebo-controlled, multicenter outcome study | Saper,2002 | Not TTH |
| EMG-guided botulinum toxin a alleviates severely disabled headache patients with hyperactive muscles. a double-blind randomized placebo-controlled cross-over study | Knudsen,2018 | Not TTH |
| Evaluation of the prophylactic efficacy of amitriptyline and citalopram, alone or in combination, in patients with comorbidity of depression, migraine, and tension-type headache | Rampello,2004 | Not TTH |
| Occipital Nerve Block Compared With Acetaminophen and Caffeine for Headache Treatment in Pregnancy A Randomized Controlled Trial | Bushman,2023 | Not TTH |
| Single-blind, randomized, pilot study combining shiatsu and amitriptyline in refractory primary headaches | Villani,2017 | Not TTH |
| Sodium valproate in severe migraine and tension-type headache: an open study of long-term efficacy and correlation with blood levels. | Lenaerts,1996 | Not TTH |
| The effectiveness comparison of valproic acid 500 mg and amitriptyline 15 mg in reducing the frequency of headache attack in patients with tension-type headache | Susanti,2020 | Not ICHD |
| The effectiveness of a group-based acceptance and commitment additive therapy on rehabilitation of female outpatients with chronic headache: preliminary findings reducing 3 dimensions of headache impact | Mo'tamedi,2012 | Not TTH |
| A personalized approach to the treatment of chronic tension-type headache: quality of life of patients | Tlisova,2024 | Not targeted interventions |
| A role of treatment of autonomic syndrome in patients with tension-type headache | Duma,2015 | Not targeted interventions |
| A trial to prove the efficacy of acupuncture as a therapeutic support in pharmacological prophylaxis for migraine and tension-type headache. Pilot study | Olivier,2022 | Not targeted interventions |
| An open label, randomised, pilot study of botulinum toxin type-A in the treatment of chronic tension-type headache | Porta,2004 | Not targeted interventions |
| Botulinum toxin type A injections for myofascial pain syndrome and tension-type headache | Porta,1999 | Not targeted interventions |
| Carthami-Semen acupuncture point injection for chronic daily headache: A pilot, randomised, double-blind, controlled trial | Park,2011 | Not targeted interventions |
| Central synaptic neurotransmission: universal self-regulation theory | Majkova,2006 | Not targeted interventions |
| Changing perceptions and efficacy of generic medicines: An intervention study | Colgan,2016 | Not targeted interventions |
| Chronic tension-type headache management with the emotional freedom technique | Bougea,2013 | Not targeted interventions |
| Combination of tizanidine and amitriptyline in the prophylaxis of chronic tension-type headache: evaluation of efficacy and impact on quality of life | Bettucci,2006 | Not targeted interventions |
| Combination treatment of psychotherapy and medication for chronic tension-type headache | Jia,2004 | Not targeted interventions |
| Comparison of Traditional Therapy Versus Biofeedback for Tension Type and Migraine Headaches | Nathu,2020 | Not targeted interventions |
| Different effects of tibolone and low-dose EPT in the management of postmenopausal women with primary headaches | Nappi,2006 | Not targeted interventions |
| Effect of rajyoga meditation on chronic tension headache | Kiran,2014 | Not targeted interventions |
| Effectiveness of suboccipital muscle inhibition combined with interferential current in patients with chronic tension-type headache: a randomised controlled clinical trial | Pérez-Llanes,2022 | Not targeted interventions |
| Effects of acupuncture and medical training therapy on depression, anxiety, and quality of life in patients with frequent tension-type headache: a randomized controlled study | Schiller,2023 | Not targeted interventions |
| Effects of occlusal splint therapy in patients with migraine or tension-type headache and comorbid temporomandibular disorder: A randomized controlled trial | Saha,2019 | Not targeted interventions |
| Effects of Valeriana officinalis (Valerian) on tension-type headache: a randomized, placebo-controlled, double-blind clinical trial | Azizi,2020 | Not targeted interventions |
| Efficacy of neuroprotectors in patients with tension headaches. | Medvedeva,2007 | Not targeted interventions |
| Interdisciplinary approach (neurologist and psychiatrist) in the treatment of chronic tension-type headaches | Ashibokova,2025 | Not targeted interventions |
| Migraine and the noradrenergic control of vasomotricity: A study with alpha-2 stimulant and alpha-2 blocker drugs | Martucci,1985 | Not targeted interventions |
| Supraorbital electrical stimulation in management of chronic type tension headache: A randomized controlled study | Hamed,2018 | Not targeted interventions |
| The Addition of Diacutaneous Fibrolysis to a Pharmacological Intervention in Patients with Tension-Type Headache: a Randomized Controlled Trial | Cabanillas-Barea,2022 | Not targeted interventions |
| The effect of manual therapies on tension-type headache in patients who do not respond to drug therapy: a randomized clinical trial | Azhdari,2023 | Not targeted interventions |
| The effect of trigger point management by positional release therapy on tension type headache | Ghanbari,2012 | Not targeted interventions |
| The effectiveness of etoricoxib in the treatment of severe exacerbation of chronic daily tension headache (CDTH) | Elchami,2011 | Not targeted interventions |
| The effectiveness of etoricoxib plus lidocaine patches 5% in the treatment of allodynia in chronic daily tension headache (CDTH) | Elcham,2013 | Not targeted interventions |
| The Efficacy of Manual Therapy and Pressure Biofeedback-Guided Deep Cervical Flexor Muscle Strength Training on Pain and Functional Limitations in Individuals with Cervicogenic Headaches: A Randomized Comparative Study | Hasan,2023 | Not targeted interventions |
| The headache management trial: A randomized study of coordinated care | Matchar,2008 | Not targeted interventions |
| Transcranial Doppler ultrasound in migraine and tension-type headache after apomorphine administration: Double-blind crossover versus placebo study | Piccini,1995 | Not targeted interventions |
| Treating chronic tension-type headache not responding to amitriptyline hydrochloride with paroxetine hydrochloride: A pilot evaluation | Holroyd,2003 | Not targeted interventions |
| A comparison of pharmacological (amitriptyline HCL) and nonpharmacological (cognitive-behavioral) therapies for chronic tension headaches | Holroyd,1991 | Not targeted control |
| A Randomized Controlled Trial on the Effectiveness of Court-Type Traditional Thai Massage versus Amitriptyline in Patients with Chronic Tension-Type Headache | Damapong,2015 | Not targeted control |
| Comparison between efficacy of imipramine and transcutaneous electrical nerve stimulation in the prophylaxis of chronic tension-type headache: A randomized controlled clinical trial | Mousavi,2011 | Not targeted control强化治疗 |
| Effects of amitriptyline and intra-oral device appliance on clinical and laser-evoked potentials features in chronic tension-type headache | de Tommaso,2005 | Not targeted control |
| Intra-oral orthosis vs amitriptyline in chronic tension-type headache: a clinical and laser evoked potentials study | de Tommaso,2006 | Not targeted control |
| Osteopathic Manipulative Therapy in Patients With Chronic Tension-Type Headache: a Pilot Study | Deodato,2019 | Not targeted control |
| Spinal manipulation vs. amitriptyline for the treatment of chronic tension-type headaches: A randomized clinical trial | Boline,1995 | Not targeted control |
| Acetylsalicylic acid activates antinociceptive brain-stem reflex activity in headache patients and in healthy subjects | Göbel,1992 | Not targeted outcome |
| Amitriptyline versus tizanidine in the management of tension-type headache in Nepal. | Thapa,2013 | Not targeted outcome |
| Botulinum toxin A for chronic daily headache: A randomized, placebo-controlled, parallel design study | Ondo,2004 | Not targeted outcome |
| Botulinum toxin type A and EMG: A key to the understanding of chronic tension-type headaches? | Rollnik,2001 | Not targeted outcome |
| Dextroamphetamine pilot crossover trials and n of 1 trials in patients with chronic tension-type and migraine headache | Haas,2004 | Not targeted outcome |
| Effect of serotonergic antidepressant therapy on temperament and character scales in patients with chronic tension-type headache. | Boz,2007 | Not targeted outcome |
| Effect of sulpiride or paroxetine on cerebrospinal fluid neuropeptide concentrations in patients with chronic tension-type headache | Bach,1994 | Not targeted outcome |
| Influence of venlafaxine hydrochloride on the quality of life in patients with chronic tension headache | Zhou,2006 | Not targeted outcome |
| Local infiltration of gonyautoxin is safe and effective in treatment of chronic tension-type headache | Lattes,2009 | Not targeted outcome |
| Moderation and mediation in the psychological and drug treatment of chronic tension-type headache: the role of disorder severity and psychiatric comorbidity. | Holroyd,2009 | Not targeted outcome |
| A comparison of naproxen sodium, acetaminophen and placebo in the treatment of muscle contraction headache | Miller,1987 | Not prophylactic treatment |
| A randomized trial of intravenous ketorolac versus intravenous metoclopramide plus diphenhydramine for tension-type and all nonmigraine, noncluster recurrent headaches | Friedman,2013 | Not prophylactic treatment |
| Analgesic efficacy and tolerability of locally applied oleum menthae piperitae preparation LI 170 in patients with migraine or tension-type headache | Gobel,2001 | Not prophylactic treatment |
| Aspirin in episodic tension-type headache: Placebo-controlled dose-ranging comparison with paracetamol | Steiner,2003 | Not prophylactic treatment |
| Assessment of the Efficacy and Safety Profiles of Aspirin and Acetaminophen With Codeine: Results From 2 Randomized, Controlled Trials in Individuals With Tension-Type Headache and Postoperative Dental Pain | Gatoulis,2012 | Not prophylactic treatment |
| Caffeine as an analgesic adjuvant in tension headache | Migliardi,1994 | Not prophylactic treatment |
| Comparative efficacy of ibuprofen arginine and β-cyclodextrin piroxicam as treatment for tension-type headache | Laveneziana,1996 | Not prophylactic treatment |
| Comparison of 650 mg aspirin and 1,000 mg acetaminophen with each other, and with placebo in moderately severe headaches | Peters,1983 | Not prophylactic treatment |
| Comparison of Intravenous Metoclopramide and Acetaminophen in Primary Headaches: a Randomized Controlled Trial | Faridaalaee,2015 | Not prophylactic treatment |
| Comparison of ketoprofen, ibuprofen and naproxen sodium in the treatment of tension-type headache | Lange,1995 | Not prophylactic treatment |
| Controlled trial of ketorolac in tension-type headache | Harden,1998 | Not prophylactic treatment |
| Dipyrone plus caffeine versus dipyrone alone to acute treatment of mild to moderate primary headache attacks: A double blind, randomized, compared clinical study | Carvalho,2007 | Not prophylactic treatment |
| Effect of inhibition of nitric oxide synthase on chronic tension-type headache: a randomised crossover trial | Ashina,1999 | Not prophylactic treatment |
| Effectiveness of Oleum menthae piperitae and paracetamol in therapy of headache of the tension type | Göbel,1996 | Not prophylactic treatment |
| Efficacy and safety of acetaminophen and naproxen in the treatment of tension-type headache. A randomized, double-blind, placebo-controlled trial | Prior,2002 | Not prophylactic treatment |
| Efficacy and safety of an ethanolic solution of peppermint oil for patients with episodic Tension type headache: EUMINZ® a randomized controlled trial | Shah,2017 | Not prophylactic treatment |
| Efficacy and safety of metamizol vs. acetylsalicylic acid in patients with moderate episodic tension-type headache: a randomized, double-blind, placebo- and active-controlled, multicentre study | Martínez-Martín,2001 | Not prophylactic treatment |
| Efficacy and safety of two fast-absorbing formulations of paracetamol in combination with caffeine for episodic tension-type headache: results from two randomized placebo- and active-controlled trials | Yue,2017 | Not prophylactic treatment |
| Efficacy and tolerability of combined dipyrone, isometheptene and caffeine in the treatment of mild-to-moderate primary headache episodes | De Souza Carvalho,2012 | Not prophylactic treatment |
| Efficacy of a fixed combination of indomethacin, prochlorperazine, and caffeine in the treatment of episodic tension-type headache: a double-blind, randomized, nimesulide-controlled, parallel group, multicentre trial | Cerbo,2005 | Not prophylactic treatment |
| Evaluation of placebo use in migraine without aura, migraine with aura and episodic tension-type headache acute attacks | Bigal,2001 | Not prophylactic treatment |
| Fiorinal® with Codeine in the management of tension headache: Impact of placebo response | Hwang,1987 | Not prophylactic treatment |
| Fiorinal® with codeine in the treatment of tension headache - The contribution of components to the combination drug | Friedman,1988 | Not prophylactic treatment |
| Greater Occipital Nerve Blockade in the Treatment of Tension-type Headaches in the Emergency Department | Çimen,2024 | Not prophylactic treatment |
| I-FiBH trial: Intravenous fluids in benign headaches - A randomised, single-blinded clinical trial | Zitek,2020 | Not prophylactic treatment |
| Ibuprofen plus caffeine in the treatment of tension-type headache | Diamond,2000 | Not prophylactic treatment |
| Inhibition of nitric oxide synthase has an analgesic effect in chronic pain | Ashina,2000 | Not prophylactic treatment |
| Intravenous chlorpromazine in the acute treatment of episodic tension-type headache: A randomized, placebo controlled, double-blind study | Bigal,2002 | Not prophylactic treatment |
| Intravenous dipyrone for the acute treatment of episodic tension-type headache: a randomized, placebo-controlled, double-blind study | Bigal,2002 | Not prophylactic treatment |
| Intravenous metamizol (Dipyrone) in acute migraine treatment and in episodic tension-type headache - A placebo-controlled study | Bigal,2001 | Not prophylactic treatment |
| Intravenous versus rectal prochlorperazine in the treatment of benign vascular or tension headache: A randomized, prospective, double-blind trial | Thomas,1994 | Not prophylactic treatment |
| Is the combination of ibuprofen and caffeine effective for the treatment of a tension-type headache? | Sparano,2001 | Not prophylactic treatment |
| Ketoprofen (25 mg) in the symptomatic treatment of episodic tension- type headache: Double-blind placebo-controlled comparison with acetaminophen (1000 mg) | Steiner,1998 | Not prophylactic treatment |
| Ketoprofen, acetaminophen, and placebo in the treatment of tension headache | Mehlisch,1998 | Not prophylactic treatment |
| Ketoprofen, paracetamol and placebo in the treatment of episodic tension-type headache | Dahlöf,1996 | Not prophylactic treatment |
| Lidocaine versus dexketoprofen in treatment of tension-type headache: a double-blind randomized controlled trial | Akbas,2021 | Not prophylactic treatment |
| Low-dose diclofenac potassium in the treatment of episodic tension-type headache | Kubitzek,2003 | Not prophylactic treatment |
| Low-dose ibuprofen in self-medication of mild to moderate headache: A comparison with acetylsalicylic acid and placebo | Nebe,1995 | Not prophylactic treatment |
| Lumiracoxib is effective in the treatment of episodic tension-type headache | Packman,2005 | Not prophylactic treatment |
| Mint oil solution in tension headache is comparatively as effective as paracetamol or acetylsalicylic acid | Göbel,2001 | Not prophylactic treatment |
| Multi-center randomized control trial of etizolam plus NSAID combination for tension-type headache | Hirata,2007 | Not prophylactic treatment |
| Muscle contraction (tension) headache: a double-blind study comparing the efficacy and safety of meprobamate-aspirin with butalbital-aspirin-phenacetin-caffeine | Glassman,1980 | Not prophylactic treatment |
| Nitric oxide-induced headache in patients with chronic tension-type headache | Ashina,2000 | Not prophylactic treatment |
| Nonprescription ibuprofen and acetaminophen in the treatment of tension- type headache | Schachtel,1996 | Not prophylactic treatment |
| Oleum menthae piperitae in the acute therapy of migraine and tension-type headache | Gobel,2004 | Not prophylactic treatment |
| Onset of analgesia with ibuprofen sodium in tension-type headache: a randomized trial | Packman,2015 | Not prophylactic treatment |
| Possible mechanisms of action of nitric oxide synthase inhibitors in chronic tension-type headache | Ashina,1999 | Not prophylactic treatment |
| Possible mechanisms of pain perception in patients with episodic tension-type headache. A new experimental model of myofascial pain | Mørk,2004 | Not prophylactic treatment |
| Proquazone for tension headache - A multicenter trial | DiSerio,1985 | Not prophylactic treatment |
| Prospective, randomised, double blind, controlled comparison of metaclopramide and pethidine in the emergency treatment of acute primary vascular and tension type headache episodes | Cicek,2004 | Not prophylactic treatment |
| Randomised trial of IV metoclopramide vs IV ketorolac in treatment of acute primary headaches | Soltani,2021 | Not prophylactic treatment |
| Randomized double-blind trial of intravenous prochlorperazine for the treatment of acute headache | Jones,1989 | Not prophylactic treatment |
| Randomized trial of adding parenteral acetaminophen to prochlorperazine and diphenhydramine to treat headache in the emergency department | Meyering,2017 | Not prophylactic treatment |
| Relationship between Chronic Tension-type Headache, Cranial Hemodynamics, and Cerebrospinal Pressure: Study Involving Provocation with Sumatriptan | Hannerz,2004 | Not prophylactic treatment |
| Responsiveness of non-IHS migraine and tension-type headache to sumatriptan | Cady,1997 | Not prophylactic treatment |
| Safety of a novel formulation of ibuprofen sodium compared with standard ibuprofen and placebo | Jayawardena,2015 | Not prophylactic treatment |
| Self-medication of a single headache episode with ketoprofen, ibuprofen or placebo, home-monitored with an electronic patient diary | Van Gerven,1996 | Not prophylactic treatment |
| Solubilized ibuprofen: evaluation of onset, relief, and safety of a novel formulation in the treatment of episodic tension-type headache | Packman,2000 | Not prophylactic treatment |
| Sumatriptan for the treatment of undifferentiated primary headaches in the ED | Miner,2007 | Not prophylactic treatment |
| Sumatriptan has no clinically relevant effect in the treatment of episodic tension-type headache | Brennum,1996 | Not prophylactic treatment |
| Symptomatic treatment of chronically recurring tension headache: A placebo-controlled, multicenter investigation of Fioricet® and acetaminophen with codeine | Friedman,1987 | Not prophylactic treatment |
| The 5-HT1-like agonist sumatriptan has a significant effect in chronic tension-type headache. | Brennum,1992 | Not prophylactic treatment |
| The combination of ASA, paracetamol and caffeine is efficacious in the treatment of severe headache attacks: Results from a multicentre, randomized, double-blind, placebo-controlled study | Diener,2011 | Not prophylactic treatment |
| The efficacy and tolerability of a fixed combination of acetylsalicylic acid, paracetamol, and caffeine in patients with severe headache: A post-hoc subgroup analysis from a multicentre, randomized, double-blind, single-dose, placebo-controlled parallel group study | Diener,2011 | Not prophylactic treatment |
| The fixed combination of acetylsalicylic acid, paracetamol and caffeine is more effective than single substances and dual combination for the treatment of headache: A multicentre, randomized, double-blind, single-dose, placebo-controlled parallel group study | Diener,2005 | Not prophylactic treatment |
| The use of ibuprofen plus caffeine to treat tension-type headache. | Diamond,2001 | Not prophylactic treatment |
| Tolerability and efficacy of a combination of paracetamol and caffeine in the treatment of tension-type headache: A randomised, double-blind, double-dummy, cross-over study versus placebo and naproxen sodium | Pini,2008 | Not prophylactic treatment |
| Treatment of episodic tension-type headache with a novel formulation of ibuprofen sodium | Packman,2013 | Not prophylactic treatment |
| Treatment of episodic tension-type headache with a novel formulation of ibuprofen sodium | Packman,2014 | Not prophylactic treatment |
| Treatment of muscle contraction headache: Micrainin vs. aspirin | Glassman,1982 | Not prophylactic treatment |
| Amitriptyline in the treatment of chronic tension-type headache | Gobel,1994 | Duplicate |
| Amitriptyline reduces myofascial tenderness in patients with chronic tension-type headache | Bendtsen,2000 | Duplicate |
| Amitriptyline versus amitriptyline-N-oxide versus placebo in the treatment of chronic tension type headache: a multi-centre, randomised parallel-group double-blind study | Pfaffenrath,1991 | Duplicate |
| Amitriptyline, a combined serotonin and noradrenaline re-uptake inhibitor, reduces exteroceptive suppression of temporal muscle activity in patients with chronic tension-type headache | Bendtsen,1996 | Duplicate |
| Analgesic effect of amitriptyline in chronic tension-type headache is not directly related to serotonin reuptake inhibition | Ashina,2004 | Duplicate |
| Blindness and bias in a trial of antidepressant medication for chronic tension-type headache | Holroyd,2006 | Duplicate |
| Botulinum toxin (DYSPORT) in tension-type headaches | Rollnik,2002 | Duplicate |
| Comparison ketoprofen, ibuprofen and naproxen sodium in the treatment of tension-type headache | Lange,1995 | Duplicate |
| [Effectiveness and tolerance of amitriptyline oxide in chronic tension headache--a multicenter double-blind study versus amitriptyline versus placebo]. | Pfaffenrath,1993 | Duplicate |
| Separate and combined effects of tricyclic antidepressant medication and cognitive-behavior therapy in the management of chronic tension-type headaches: a randomized controlled trial | Cordingley,2001 | Duplicate |
| The efficacy of local lidocaine injection in patients with frequent episodic tension-type headache: a randomized, placebo-controlled double-blind clinical trial | Karadas,2011 | Duplicate |
| Treatment of chronic tension-type headache with mirtazapine and amitriptyline | Martín-Araguz,2003 | Duplicate |

Table S5. Summary of fixed-effects and random-effects model fit statistics from network meta-analysis

| **Model** | **Number of data points** | **Posterior total residual deviance** | **DIC** |
| --- | --- | --- | --- |
| **Headache days per month** | | | |
| **4W** | | | |
| **FE consistency** | 15 | 13.5 | 26.6 |
| **FE inconsistency** | 15 | 13.6 | 26.6 |
| **RE consistency** | 15 | 13.7 | 27 |
| **8W** | | | |
| **FE consistency** | 32 | 26.4 | 52.4 |
| **FE inconsistency** | 32 | 26.4 | 52.4 |
| **RE consistency** | 32 | 27.3 | 54.3 |
| **12W** | | | |
| **FE consistency** | 24 | 28.6 | 47.6 |
| **FE inconsistency** | 24 | 28.3 | 46.9 |
| **RE consistency** | 24 | 21.3 | 41.4 |
| **24W** | | | |
| **FE consistency** | 4 | 4 | 8 |
| **FE inconsistency** | 4 | 4 | 7.8 |
| **RE consistency** | 4 | 3.9 | 7.8 |
| **Headache intensity** | | | |
| **4W** | | | |
| **FE consistency** | 15 | 13.4 | 26.7 |
| **FE inconsistency** | 15 | 13.6 | 27.1 |
| **RE consistency** | 15 | 14.0 | 27.9 |
| **8W** | | | |
| **FE consistency** | 26 | 23.3 | 46.4 |
| **FE inconsistency** | 26 | 23.5 | 46.8 |
| **RE consistency** | 26 | 23.7 | 47.4 |
| **12W** | | | |
| **FE consistency** | 22 | 20.1 | 39.8 |
| **FE inconsistency** | 22 | 20.2 | 40.0 |
| **RE consistency** | 22 | 20.5 | 40.8 |
| **24W** | | | |
| **FE consistency** | 4 | 4.1 | 8.2 |
| **FE inconsistency** | 4 | 4.1 | 8.1 |
| **RE consistency** | 4 | 3.9 | 7.7 |
| **Headache duration** | | | |
| **4W** | | | |
| **FE consistency** | 8 | 6.8 | 13.6 |
| **FE inconsistency** | 8 | 7.0 | 13.9 |
| **RE consistency** | 8 | 7.1 | 14.1 |
| **8W** | | | |
| **FE consistency** | 14 | 11.2 | 22.3 |
| **FE inconsistency** | 14 | 10.9 | 21.7 |
| **RE consistency** | 14 | 11.1 | 22.2 |
| **12W** | | | |
| **FE consistency** | 10 | 8.2 | 16.4 |
| **FE inconsistency** | 10 | 8.2 | 16.4 |
| **RE consistency** | 10 | 8.4 | 16.7 |
| **Adverse event rate** | | | |
| **FE consistency** | 41 | 45.2 | 82.3 |
| **FE inconsistency** | 41 | 45.3 | 82.4 |
| **RE consistency** | 41 | 41.9 | 81.7 |

**Footnote:** DIC: deviance information criteria; FE: fixed-effects; RE: random-effects.

Table S6. Certainty of evidence of outcomes

| Comparison | Number of studies | Within-study bias | Reporting bias | Indirectness | Imprecision | Heterogeneity | Incoherence | Confidence rating |
| --- | --- | --- | --- | --- | --- | --- | --- | --- |
| Headache days per month | | | | | | | | |
| 4 weeks | | | | | | | | |
| Amitriptyline 100mg:Placebo | 1 | Some concerns | Low risk | No concerns | No concerns | Some concerns | No concerns | Low |
| BTX-A 100U:Placebo | 2 | Some concerns | Low risk | No concerns | Some concerns | Some concerns | No concerns | Very low |
| BTX-A 20U:Placebo | 1 | Some concerns | Low risk | No concerns | Some concerns | No concerns | No concerns | Low |
| BTX-A 50U:Placebo | 1 | No concerns | Low risk | No concerns | Some concerns | Some concerns | No concerns | Low |
| Placebo:Sodium valproate 1g | 1 | Some concerns | Low risk | No concerns | Some concerns | Some concerns | No concerns | Very low |
| Placebo:Tizanidine 12mg | 1 | Some concerns | Low risk | No concerns | Some concerns | Some concerns | No concerns | Very low |
| Placebo:Tizanidine 6mg | 1 | Some concerns | Low risk | No concerns | Some concerns | Some concerns | No concerns | Very low |
| Tizanidine 12mg:Tizanidine 6mg | 1 | Some concerns | Low risk | No concerns | Some concerns | Major concerns | No concerns | Very low |
| Amitriptyline 100mg:BTX-A 100U | 0 | Some concerns | Low risk | Some concerns | Some concerns | Major concerns | No concerns | Very low |
| Amitriptyline 100mg:BTX-A 20U | 0 | Some concerns | Low risk | Some concerns | Some concerns | Some concerns | No concerns | Very low |
| Amitriptyline 100mg:BTX-A 50U | 0 | Some concerns | Low risk | Some concerns | Some concerns | Major concerns | No concerns | Very low |
| Amitriptyline 100mg:Sodium valproate 1g | 0 | Some concerns | Low risk | Some concerns | Some concerns | Some concerns | No concerns | Very low |
| Amitriptyline 100mg:Tizanidine 12mg | 0 | Some concerns | Low risk | Some concerns | Some concerns | Major concerns | No concerns | Very low |
| Amitriptyline 100mg:Tizanidine 6mg | 0 | Some concerns | Low risk | Some concerns | Some concerns | Major concerns | No concerns | Very low |
| BTX-A 100U:BTX-A 20U | 0 | Some concerns | Low risk | Some concerns | Some concerns | No concerns | No concerns | Very low |
| BTX-A 100U:BTX-A 50U | 0 | Some concerns | Low risk | Some concerns | Some concerns | No concerns | No concerns | Very low |
| BTX-A 100U:Sodium valproate 1g | 0 | Some concerns | Low risk | Some concerns | Some concerns | Some concerns | No concerns | Very low |
| BTX-A 100U:Tizanidine 12mg | 0 | Some concerns | Low risk | Some concerns | Some concerns | Some concerns | No concerns | Very low |
| BTX-A 100U:Tizanidine 6mg | 0 | Some concerns | Low risk | Some concerns | Some concerns | Some concerns | No concerns | Very low |
| BTX-A 20U:BTX-A 50U | 0 | Some concerns | Low risk | Some concerns | Some concerns | No concerns | No concerns | Very low |
| BTX-A 20U:Sodium valproate 1g | 0 | Some concerns | Low risk | Some concerns | Some concerns | Some concerns | No concerns | Very low |
| BTX-A 20U:Tizanidine 12mg | 0 | Some concerns | Low risk | Some concerns | Some concerns | Some concerns | No concerns | Very low |
| BTX-A 20U:Tizanidine 6mg | 0 | Some concerns | Low risk | Some concerns | Some concerns | Some concerns | No concerns | Very low |
| BTX-A 50U:Sodium valproate 1g | 0 | Some concerns | Low risk | Some concerns | Some concerns | Some concerns | No concerns | Very low |
| BTX-A 50U:Tizanidine 12mg | 0 | Some concerns | Low risk | Some concerns | Some concerns | Some concerns | No concerns | Very low |
| BTX-A 50U:Tizanidine 6mg | 0 | Some concerns | Low risk | Some concerns | Some concerns | Some concerns | No concerns | Very low |
| Sodium valproate 1g:Tizanidine 12mg | 0 | Some concerns | Low risk | Some concerns | Some concerns | Some concerns | No concerns | Very low |
| Sodium valproate 1g:Tizanidine 6mg | 0 | Some concerns | Low risk | Some concerns | Some concerns | Some concerns | No concerns | Very low |
| 8 weeks | | | | | | | | |
| Amitriptyline 100mg:Placebo | 1 | Some concerns | Low risk | No concerns | No concerns | Some concerns | No concerns | Low |
| Amitriptyline 75mg:Citalopram 20mg | 1 | Major concerns | Low risk | No concerns | No concerns | Some concerns | No concerns | Very low |
| Amitriptyline 75mg:Placebo | 1 | Major concerns | Low risk | No concerns | No concerns | Some concerns | No concerns | Very low |
| BTX-A 100U:BTX-A 150U | 1 | No concerns | Low risk | No concerns | Major concerns | No concerns | No concerns | Very low |
| BTX-A 100U:BTX-A 50U | 1 | No concerns | Low risk | No concerns | Some concerns | Some concerns | No concerns | Low |
| BTX-A 100U:BTX-A 86U | 1 | No concerns | Low risk | No concerns | Some concerns | Some concerns | No concerns | Low |
| BTX-A 100U:Placebo | 3 | Some concerns | Low risk | No concerns | No concerns | Major concerns | No concerns | Very low |
| BTX-A 150U:BTX-A 50U | 1 | No concerns | Low risk | No concerns | Major concerns | No concerns | No concerns | Low |
| BTX-A 150U:BTX-A 86U | 1 | No concerns | Low risk | No concerns | Major concerns | No concerns | No concerns | Low |
| BTX-A 150U:Placebo | 1 | No concerns | Low risk | No concerns | Some concerns | Some concerns | No concerns | Low |
| BTX-A 20U:Placebo | 1 | Some concerns | Low risk | No concerns | Major concerns | No concerns | No concerns | Very low |
| BTX-A 210U:BTX-A 420U | 1 | No concerns | Low risk | No concerns | Major concerns | No concerns | No concerns | Low |
| BTX-A 210U:Placebo | 1 | No concerns | Low risk | No concerns | Major concerns | No concerns | No concerns | Low |
| BTX-A 420U:Placebo | 1 | No concerns | Low risk | No concerns | Major concerns | No concerns | No concerns | Low |
| BTX-A 50U:BTX-A 86U | 1 | No concerns | Low risk | No concerns | Major concerns | No concerns | No concerns | Low |
| BTX-A 50U:Placebo | 1 | No concerns | Low risk | No concerns | Some concerns | Some concerns | No concerns | Low |
| BTX-A 86U:Placebo | 1 | No concerns | Low risk | No concerns | Some concerns | Some concerns | No concerns | Low |
| Citalopram 20mg:Placebo | 1 | Major concerns | Low risk | No concerns | Major concerns | No concerns | No concerns | Very low |
| Flunarizine 10mg:Flunarizine 5mg | 1 | Major concerns | Low risk | No concerns | Major concerns | No concerns | No concerns | Very low |
| Flunarizine 10mg:Placebo | 1 | Major concerns | Low risk | No concerns | Some concerns | Some concerns | No concerns | Very low |
| Flunarizine 5mg:Placebo | 1 | Major concerns | Low risk | No concerns | Major concerns | No concerns | No concerns | Very low |
| Ibuprofen 400mg:Mitazapine 4.5mg | 1 | No concerns | Low risk | No concerns | Major concerns | No concerns | No concerns | Low |
| Ibuprofen 400mg:Placebo | 1 | No concerns | Low risk | No concerns | Major concerns | No concerns | No concerns | Low |
| L-5-Hydroxytryptophan 100mg:Placebo | 1 | Major concerns | Low risk | No concerns | Major concerns | No concerns | No concerns | Very low |
| Mitazapine 30mg:Placebo | 1 | No concerns | Low risk | No concerns | Some concerns | Some concerns | No concerns | Low |
| Mitazapine 4.5mg:Placebo | 1 | No concerns | Low risk | No concerns | Major concerns | No concerns | No concerns | Low |
| Placebo:Tizanidine 12mg | 1 | Some concerns | Low risk | No concerns | Some concerns | Some concerns | No concerns | Very low |
| Placebo:Tizanidine 6mg | 1 | Some concerns | Low risk | No concerns | Some concerns | Some concerns | No concerns | Very low |
| Tizanidine 12mg:Tizanidine 6mg | 1 | Some concerns | Low risk | No concerns | No concerns | Major concerns | No concerns | Very low |
| Amitriptyline 100mg:Amitriptyline 75mg | 0 | Major concerns | Low risk | Some concerns | Some concerns | Some concerns | No concerns | Very low |
| Amitriptyline 100mg:BTX-A 100U | 0 | Some concerns | Low risk | Some concerns | No concerns | Major concerns | No concerns | Very low |
| Amitriptyline 100mg:BTX-A 150U | 0 | No concerns | Low risk | Some concerns | No concerns | Major concerns | No concerns | Very low |
| Amitriptyline 100mg:BTX-A 20U | 0 | Some concerns | Low risk | Some concerns | No concerns | Major concerns | No concerns | Very low |
| Amitriptyline 100mg:BTX-A 210U | 0 | No concerns | Low risk | Some concerns | No concerns | Major concerns | No concerns | Very low |
| Amitriptyline 100mg:BTX-A 420U | 0 | No concerns | Low risk | Some concerns | No concerns | Major concerns | No concerns | Very low |
| Amitriptyline 100mg:BTX-A 50U | 0 | No concerns | Low risk | Some concerns | No concerns | Major concerns | No concerns | Very low |
| Amitriptyline 100mg:BTX-A 86U | 0 | No concerns | Low risk | Some concerns | No concerns | Major concerns | No concerns | Very low |
| Amitriptyline 100mg:Citalopram 20mg | 0 | Major concerns | Low risk | Some concerns | No concerns | Major concerns | No concerns | Very low |
| Amitriptyline 100mg:Flunarizine 10mg | 0 | Major concerns | Low risk | Some concerns | Major concerns | No concerns | No concerns | Very low |
| Amitriptyline 100mg:Flunarizine 5mg | 0 | Major concerns | Low risk | Some concerns | Major concerns | No concerns | No concerns | Very low |
| Amitriptyline 100mg:Ibuprofen 400mg | 0 | No concerns | Low risk | Some concerns | No concerns | Major concerns | No concerns | Very low |
| Amitriptyline 100mg:L-5-Hydroxytryptophan 100mg | 0 | Major concerns | Low risk | Some concerns | No concerns | Major concerns | No concerns | Very low |
| Amitriptyline 100mg:Mitazapine 30mg | 0 | Some concerns | Low risk | Some concerns | Some concerns | Some concerns | No concerns | Very low |
| Amitriptyline 100mg:Mitazapine 4.5mg | 0 | No concerns | Low risk | Some concerns | No concerns | Major concerns | No concerns | Very low |
| Amitriptyline 100mg:Tizanidine 12mg | 0 | Some concerns | Low risk | Some concerns | No concerns | Major concerns | No concerns | Very low |
| Amitriptyline 100mg:Tizanidine 6mg | 0 | Some concerns | Low risk | Some concerns | No concerns | Major concerns | No concerns | Very low |
| Amitriptyline 75mg:BTX-A 100U | 0 | Some concerns | Low risk | Some concerns | Major concerns | No concerns | No concerns | Very low |
| Amitriptyline 75mg:BTX-A 150U | 0 | Some concerns | Low risk | Some concerns | Major concerns | No concerns | No concerns | Very low |
| Amitriptyline 75mg:BTX-A 20U | 0 | Major concerns | Low risk | Some concerns | Some concerns | Some concerns | No concerns | Very low |
| Amitriptyline 75mg:BTX-A 210U | 0 | Some concerns | Low risk | Some concerns | Major concerns | No concerns | No concerns | Very low |
| Amitriptyline 75mg:BTX-A 420U | 0 | Some concerns | Low risk | Some concerns | Some concerns | Some concerns | No concerns | Very low |
| Amitriptyline 75mg:BTX-A 50U | 0 | Some concerns | Low risk | Some concerns | Major concerns | No concerns | No concerns | Very low |
| Amitriptyline 75mg:BTX-A 86U | 0 | Some concerns | Low risk | Some concerns | Major concerns | No concerns | No concerns | Very low |
| Amitriptyline 75mg:Flunarizine 10mg | 0 | Major concerns | Low risk | Some concerns | Major concerns | No concerns | No concerns | Very low |
| Amitriptyline 75mg:Flunarizine 5mg | 0 | Major concerns | Low risk | Some concerns | Major concerns | No concerns | No concerns | Very low |
| Amitriptyline 75mg:Ibuprofen 400mg | 0 | Some concerns | Low risk | Some concerns | Major concerns | No concerns | No concerns | Very low |
| Amitriptyline 75mg:L-5-Hydroxytryptophan 100mg | 0 | Major concerns | Low risk | Some concerns | Some concerns | Some concerns | No concerns | Very low |
| Amitriptyline 75mg:Mitazapine 30mg | 0 | Some concerns | Low risk | Some concerns | Major concerns | No concerns | No concerns | Very low |
| Amitriptyline 75mg:Mitazapine 4.5mg | 0 | Some concerns | Low risk | Some concerns | Some concerns | Some concerns | No concerns | Very low |
| Amitriptyline 75mg:Tizanidine 12mg | 0 | Some concerns | Low risk | Some concerns | No concerns | Major concerns | No concerns | Very low |
| Amitriptyline 75mg:Tizanidine 6mg | 0 | Major concerns | Low risk | Some concerns | Some concerns | Some concerns | No concerns | Very low |
| BTX-A 100U:BTX-A 20U | 0 | Some concerns | Low risk | Some concerns | Some concerns | Some concerns | No concerns | Very low |
| BTX-A 100U:BTX-A 210U | 0 | No concerns | Low risk | Some concerns | Some concerns | Some concerns | No concerns | Very low |
| BTX-A 100U:BTX-A 420U | 0 | No concerns | Low risk | Some concerns | Some concerns | Some concerns | No concerns | Very low |
| BTX-A 100U:Citalopram 20mg | 0 | Some concerns | Low risk | Some concerns | Some concerns | Some concerns | No concerns | Very low |
| BTX-A 100U:Flunarizine 10mg | 0 | Some concerns | Low risk | Some concerns | Major concerns | No concerns | No concerns | Very low |
| BTX-A 100U:Flunarizine 5mg | 0 | Some concerns | Low risk | Some concerns | Major concerns | No concerns | No concerns | Very low |
| BTX-A 100U:Ibuprofen 400mg | 0 | No concerns | Low risk | Some concerns | Major concerns | No concerns | No concerns | Very low |
| BTX-A 100U:L-5-Hydroxytryptophan 100mg | 0 | Some concerns | Low risk | Some concerns | Some concerns | Some concerns | No concerns | Very low |
| BTX-A 100U:Mitazapine 30mg | 0 | No concerns | Low risk | Some concerns | Major concerns | No concerns | No concerns | Very low |
| BTX-A 100U:Mitazapine 4.5mg | 0 | No concerns | Low risk | Some concerns | Some concerns | Some concerns | No concerns | Very low |
| BTX-A 100U:Tizanidine 12mg | 0 | Some concerns | Low risk | Some concerns | No concerns | Major concerns | No concerns | Very low |
| BTX-A 100U:Tizanidine 6mg | 0 | Some concerns | Low risk | Some concerns | Some concerns | Some concerns | No concerns | Very low |
| BTX-A 150U:BTX-A 20U | 0 | No concerns | Low risk | Some concerns | Major concerns | No concerns | No concerns | Very low |
| BTX-A 150U:BTX-A 210U | 0 | No concerns | Low risk | Some concerns | Major concerns | No concerns | No concerns | Very low |
| BTX-A 150U:BTX-A 420U | 0 | No concerns | Low risk | Some concerns | Major concerns | No concerns | No concerns | Very low |
| BTX-A 150U:Citalopram 20mg | 0 | Some concerns | Low risk | Some concerns | Major concerns | No concerns | No concerns | Very low |
| BTX-A 150U:Flunarizine 10mg | 0 | Some concerns | Low risk | Some concerns | Major concerns | No concerns | No concerns | Very low |
| BTX-A 150U:Flunarizine 5mg | 0 | Some concerns | Low risk | Some concerns | Major concerns | No concerns | No concerns | Very low |
| BTX-A 150U:Ibuprofen 400mg | 0 | No concerns | Low risk | Some concerns | Major concerns | No concerns | No concerns | Very low |
| BTX-A 150U:L-5-Hydroxytryptophan 100mg | 0 | Some concerns | Low risk | Some concerns | Major concerns | No concerns | No concerns | Very low |
| BTX-A 150U:Mitazapine 30mg | 0 | No concerns | Low risk | Some concerns | Major concerns | No concerns | No concerns | Very low |
| BTX-A 150U:Mitazapine 4.5mg | 0 | No concerns | Low risk | Some concerns | Some concerns | Some concerns | No concerns | Very low |
| BTX-A 150U:Tizanidine 12mg | 0 | Some concerns | Low risk | Some concerns | Some concerns | Some concerns | No concerns | Very low |
| BTX-A 150U:Tizanidine 6mg | 0 | Some concerns | Low risk | Some concerns | Major concerns | No concerns | No concerns | Very low |
| BTX-A 20U:BTX-A 210U | 0 | No concerns | Low risk | Some concerns | Major concerns | No concerns | No concerns | Very low |
| BTX-A 20U:BTX-A 420U | 0 | No concerns | Low risk | Some concerns | Major concerns | No concerns | No concerns | Very low |
| BTX-A 20U:BTX-A 50U | 0 | No concerns | Low risk | Some concerns | Major concerns | No concerns | No concerns | Very low |
| BTX-A 20U:BTX-A 86U | 0 | No concerns | Low risk | Some concerns | Major concerns | No concerns | No concerns | Very low |
| BTX-A 20U:Citalopram 20mg | 0 | Major concerns | Low risk | Some concerns | Major concerns | No concerns | No concerns | Very low |
| BTX-A 20U:Flunarizine 10mg | 0 | Major concerns | Low risk | Some concerns | Major concerns | No concerns | No concerns | Very low |
| BTX-A 20U:Flunarizine 5mg | 0 | Major concerns | Low risk | Some concerns | Major concerns | No concerns | No concerns | Very low |
| BTX-A 20U:Ibuprofen 400mg | 0 | No concerns | Low risk | Some concerns | Major concerns | No concerns | No concerns | Very low |
| BTX-A 20U:L-5-Hydroxytryptophan 100mg | 0 | Major concerns | Low risk | Some concerns | Major concerns | No concerns | No concerns | Very low |
| BTX-A 20U:Mitazapine 30mg | 0 | Some concerns | Low risk | Some concerns | Major concerns | No concerns | No concerns | Very low |
| BTX-A 20U:Mitazapine 4.5mg | 0 | No concerns | Low risk | Some concerns | Major concerns | No concerns | No concerns | Very low |
| BTX-A 20U:Tizanidine 12mg | 0 | Some concerns | Low risk | Some concerns | Major concerns | No concerns | No concerns | Very low |
| BTX-A 20U:Tizanidine 6mg | 0 | Some concerns | Low risk | Some concerns | Major concerns | No concerns | No concerns | Very low |
| BTX-A 210U:BTX-A 50U | 0 | No concerns | Low risk | Some concerns | Major concerns | No concerns | No concerns | Very low |
| BTX-A 210U:BTX-A 86U | 0 | No concerns | Low risk | Some concerns | Major concerns | No concerns | No concerns | Very low |
| BTX-A 210U:Citalopram 20mg | 0 | Some concerns | Low risk | Some concerns | Major concerns | No concerns | No concerns | Very low |
| BTX-A 210U:Flunarizine 10mg | 0 | Some concerns | Low risk | Some concerns | Major concerns | No concerns | No concerns | Very low |
| BTX-A 210U:Flunarizine 5mg | 0 | Some concerns | Low risk | Some concerns | Major concerns | No concerns | No concerns | Very low |
| BTX-A 210U:Ibuprofen 400mg | 0 | No concerns | Low risk | Some concerns | Major concerns | No concerns | No concerns | Very low |
| BTX-A 210U:L-5-Hydroxytryptophan 100mg | 0 | Some concerns | Low risk | Some concerns | Major concerns | No concerns | No concerns | Very low |
| BTX-A 210U:Mitazapine 30mg | 0 | No concerns | Low risk | Some concerns | Major concerns | No concerns | No concerns | Very low |
| BTX-A 210U:Mitazapine 4.5mg | 0 | No concerns | Low risk | Some concerns | Major concerns | No concerns | No concerns | Very low |
| BTX-A 210U:Tizanidine 12mg | 0 | No concerns | Low risk | Some concerns | Some concerns | Some concerns | No concerns | Very low |
| BTX-A 210U:Tizanidine 6mg | 0 | No concerns | Low risk | Some concerns | Major concerns | No concerns | No concerns | Very low |
| BTX-A 420U:BTX-A 50U | 0 | No concerns | Low risk | Some concerns | Major concerns | No concerns | No concerns | Very low |
| BTX-A 420U:BTX-A 86U | 0 | No concerns | Low risk | Some concerns | Major concerns | No concerns | No concerns | Very low |
| BTX-A 420U:Citalopram 20mg | 0 | Some concerns | Low risk | Some concerns | Major concerns | No concerns | No concerns | Very low |
| BTX-A 420U:Flunarizine 10mg | 0 | Some concerns | Low risk | Some concerns | Major concerns | No concerns | No concerns | Very low |
| BTX-A 420U:Flunarizine 5mg | 0 | Some concerns | Low risk | Some concerns | Major concerns | No concerns | No concerns | Very low |
| BTX-A 420U:Ibuprofen 400mg | 0 | No concerns | Low risk | Some concerns | Major concerns | No concerns | No concerns | Very low |
| BTX-A 420U:L-5-Hydroxytryptophan 100mg | 0 | Some concerns | Low risk | Some concerns | Major concerns | No concerns | No concerns | Very low |
| BTX-A 420U:Mitazapine 30mg | 0 | No concerns | Low risk | Some concerns | Major concerns | No concerns | No concerns | Very low |
| BTX-A 420U:Mitazapine 4.5mg | 0 | No concerns | Low risk | Some concerns | Major concerns | No concerns | No concerns | Very low |
| BTX-A 420U:Tizanidine 12mg | 0 | Some concerns | Low risk | Some concerns | Some concerns | Some concerns | No concerns | Very low |
| BTX-A 420U:Tizanidine 6mg | 0 | Some concerns | Low risk | Some concerns | Major concerns | No concerns | No concerns | Very low |
| BTX-A 50U:Citalopram 20mg | 0 | Some concerns | Low risk | Some concerns | Major concerns | No concerns | No concerns | Very low |
| BTX-A 50U:Flunarizine 10mg | 0 | Some concerns | Low risk | Some concerns | Major concerns | No concerns | No concerns | Very low |
| BTX-A 50U:Flunarizine 5mg | 0 | Some concerns | Low risk | Some concerns | Major concerns | No concerns | No concerns | Very low |
| BTX-A 50U:Ibuprofen 400mg | 0 | No concerns | Low risk | Some concerns | Major concerns | No concerns | No concerns | Very low |
| BTX-A 50U:L-5-Hydroxytryptophan 100mg | 0 | Some concerns | Low risk | Some concerns | Major concerns | No concerns | No concerns | Very low |
| BTX-A 50U:Mitazapine 30mg | 0 | No concerns | Low risk | Some concerns | Major concerns | No concerns | No concerns | Very low |
| BTX-A 50U:Mitazapine 4.5mg | 0 | No concerns | Low risk | Some concerns | Major concerns | No concerns | No concerns | Very low |
| BTX-A 50U:Tizanidine 12mg | 0 | Some concerns | Low risk | Some concerns | Some concerns | Some concerns | No concerns | Very low |
| BTX-A 50U:Tizanidine 6mg | 0 | Some concerns | Low risk | Some concerns | Major concerns | No concerns | No concerns | Very low |
| BTX-A 86U:Citalopram 20mg | 0 | Some concerns | Low risk | Some concerns | Major concerns | No concerns | No concerns | Very low |
| BTX-A 86U:Flunarizine 10mg | 0 | Some concerns | Low risk | Some concerns | Major concerns | No concerns | No concerns | Very low |
| BTX-A 86U:Flunarizine 5mg | 0 | Some concerns | Low risk | Some concerns | Major concerns | No concerns | No concerns | Very low |
| BTX-A 86U:Ibuprofen 400mg | 0 | No concerns | Low risk | Some concerns | Major concerns | No concerns | No concerns | Very low |
| BTX-A 86U:L-5-Hydroxytryptophan 100mg | 0 | Some concerns | Low risk | Some concerns | Major concerns | No concerns | No concerns | Very low |
| BTX-A 86U:Mitazapine 30mg | 0 | No concerns | Low risk | Some concerns | Major concerns | No concerns | No concerns | Very low |
| BTX-A 86U:Mitazapine 4.5mg | 0 | No concerns | Low risk | Some concerns | Major concerns | No concerns | No concerns | Very low |
| BTX-A 86U:Tizanidine 12mg | 0 | Some concerns | Low risk | Some concerns | Some concerns | Some concerns | No concerns | Very low |
| BTX-A 86U:Tizanidine 6mg | 0 | Some concerns | Low risk | Some concerns | Major concerns | No concerns | No concerns | Very low |
| Citalopram 20mg:Flunarizine 10mg | 0 | Major concerns | Low risk | Some concerns | Some concerns | Some concerns | No concerns | Very low |
| Citalopram 20mg:Flunarizine 5mg | 0 | Major concerns | Low risk | Some concerns | Major concerns | No concerns | No concerns | Very low |
| Citalopram 20mg:Ibuprofen 400mg | 0 | Some concerns | Low risk | Some concerns | Major concerns | No concerns | No concerns | Very low |
| Citalopram 20mg:L-5-Hydroxytryptophan 100mg | 0 | Major concerns | Low risk | Some concerns | Major concerns | No concerns | No concerns | Very low |
| Citalopram 20mg:Mitazapine 30mg | 0 | Some concerns | Low risk | Some concerns | Some concerns | Some concerns | No concerns | Very low |
| Citalopram 20mg:Mitazapine 4.5mg | 0 | Some concerns | Low risk | Some concerns | Major concerns | No concerns | No concerns | Very low |
| Citalopram 20mg:Tizanidine 12mg | 0 | Some concerns | Low risk | Some concerns | Some concerns | Some concerns | No concerns | Very low |
| Citalopram 20mg:Tizanidine 6mg | 0 | Major concerns | Low risk | Some concerns | Major concerns | No concerns | No concerns | Very low |
| Flunarizine 10mg:Ibuprofen 400mg | 0 | Some concerns | Low risk | Some concerns | Major concerns | No concerns | No concerns | Very low |
| Flunarizine 10mg:L-5-Hydroxytryptophan 100mg | 0 | Major concerns | Low risk | Some concerns | Some concerns | Some concerns | No concerns | Very low |
| Flunarizine 10mg:Mitazapine 30mg | 0 | Some concerns | Low risk | Some concerns | Major concerns | No concerns | No concerns | Very low |
| Flunarizine 10mg:Mitazapine 4.5mg | 0 | Some concerns | Low risk | Some concerns | Some concerns | Some concerns | No concerns | Very low |
| Flunarizine 10mg:Tizanidine 12mg | 0 | Some concerns | Low risk | Some concerns | No concerns | Major concerns | No concerns | Very low |
| Flunarizine 10mg:Tizanidine 6mg | 0 | Some concerns | Low risk | Some concerns | Major concerns | No concerns | No concerns | Very low |
| Flunarizine 5mg:Ibuprofen 400mg | 0 | Some concerns | Low risk | Some concerns | Major concerns | No concerns | No concerns | Very low |
| Flunarizine 5mg:L-5-Hydroxytryptophan 100mg | 0 | Major concerns | Low risk | Some concerns | Major concerns | No concerns | No concerns | Very low |
| Flunarizine 5mg:Mitazapine 30mg | 0 | Some concerns | Low risk | Some concerns | Major concerns | No concerns | No concerns | Very low |
| Flunarizine 5mg:Mitazapine 4.5mg | 0 | Some concerns | Low risk | Some concerns | Major concerns | No concerns | No concerns | Very low |
| Flunarizine 5mg:Tizanidine 12mg | 0 | Some concerns | Low risk | Some concerns | Some concerns | Some concerns | No concerns | Very low |
| Flunarizine 5mg:Tizanidine 6mg | 0 | Some concerns | Low risk | Some concerns | Major concerns | No concerns | No concerns | Very low |
| Ibuprofen 400mg:L-5-Hydroxytryptophan 100mg | 0 | Some concerns | Low risk | Some concerns | Major concerns | No concerns | No concerns | Very low |
| Ibuprofen 400mg:Mitazapine 30mg | 0 | No concerns | Low risk | Some concerns | Major concerns | No concerns | No concerns | Very low |
| Ibuprofen 400mg:Tizanidine 12mg | 0 | No concerns | Low risk | Some concerns | Some concerns | Some concerns | No concerns | Very low |
| Ibuprofen 400mg:Tizanidine 6mg | 0 | No concerns | Low risk | Some concerns | Major concerns | No concerns | No concerns | Very low |
| L-5-Hydroxytryptophan 100mg:Mitazapine 30mg | 0 | Some concerns | Low risk | Some concerns | Major concerns | No concerns | No concerns | Very low |
| L-5-Hydroxytryptophan 100mg:Mitazapine 4.5mg | 0 | Some concerns | Low risk | Some concerns | Major concerns | No concerns | No concerns | Very low |
| L-5-Hydroxytryptophan 100mg:Tizanidine 12mg | 0 | Some concerns | Low risk | Some concerns | Major concerns | No concerns | No concerns | Very low |
| L-5-Hydroxytryptophan 100mg:Tizanidine 6mg | 0 | Some concerns | Low risk | Some concerns | Major concerns | No concerns | No concerns | Very low |
| Mitazapine 30mg:Mitazapine 4.5mg | 0 | No concerns | Low risk | Some concerns | Some concerns | Some concerns | No concerns | Very low |
| Mitazapine 30mg:Tizanidine 12mg | 0 | Some concerns | Low risk | Some concerns | No concerns | Major concerns | No concerns | Very low |
| Mitazapine 30mg:Tizanidine 6mg | 0 | Some concerns | Low risk | Some concerns | Major concerns | No concerns | No concerns | Very low |
| Mitazapine 4.5mg:Tizanidine 12mg | 0 | Some concerns | Low risk | Some concerns | Major concerns | No concerns | No concerns | Very low |
| Mitazapine 4.5mg:Tizanidine 6mg | 0 | Some concerns | Low risk | Some concerns | Major concerns | No concerns | No concerns | Very low |
| 12 Weeks | | | | | | | | |
| Amitriptyline 100mg:Placebo | 1 | Some concerns | Low risk | No concerns | Some concerns | Some concerns | No concerns | Very low |
| Amitriptyline 75mg:Amitriptylinoxide 90mg | 1 | No concerns | Low risk | No concerns | Some concerns | No concerns | No concerns | Moderate |
| Amitriptyline 75mg:Placebo | 1 | No concerns | Low risk | No concerns | Some concerns | Some concerns | No concerns | Low |
| Amitriptylinoxide 90mg:Placebo | 1 | No concerns | Low risk | No concerns | Major concerns | Major concerns | No concerns | Very low |
| BTX-A 100U:Placebo | 3 | Some concerns | Low risk | No concerns | Some concerns | Some concerns | No concerns | Very low |
| BTX-A 210U:BTX-A 420U | 1 | No concerns | Low risk | No concerns | No concerns | Some concerns | No concerns | Moderate |
| BTX-A 210U:Placebo | 1 | No concerns | Low risk | No concerns | No concerns | Major concerns | No concerns | Low |
| BTX-A 420U:Placebo | 1 | No concerns | Low risk | No concerns | No concerns | Major concerns | No concerns | Low |
| BTX-A 500U:Placebo | 1 | No concerns | Low risk | No concerns | No concerns | Major concerns | No concerns | Low |
| BTX-A 50U:Placebo | 1 | No concerns | Low risk | No concerns | No concerns | Major concerns | No concerns | Low |
| L-5-Hydroxytryptophan 100mg:Placebo | 1 | Major concerns | Low risk | No concerns | Major concerns | Some concerns | No concerns | Very low |
| Lidocaine 25ml:Placebo | 1 | Some concerns | Low risk | No concerns | No concerns | Major concerns | No concerns | Very low |
| Placebo:Sodium valproate 1g | 1 | Some concerns | Low risk | No concerns | No concerns | Major concerns | No concerns | Very low |
| Amitriptyline 100mg:Amitriptyline 75mg | 0 | No concerns | Low risk | Some concerns | Major concerns | Some concerns | No concerns | Very low |
| Amitriptyline 100mg:Amitriptylinoxide 90mg | 0 | No concerns | Low risk | Some concerns | Major concerns | Some concerns | No concerns | Very low |
| Amitriptyline 100mg:BTX-A 100U | 0 | Some concerns | Low risk | Some concerns | Major concerns | Some concerns | No concerns | Very low |
| Amitriptyline 100mg:BTX-A 210U | 0 | No concerns | Low risk | Some concerns | Major concerns | Some concerns | No concerns | Very low |
| Amitriptyline 100mg:BTX-A 420U | 0 | No concerns | Low risk | Some concerns | Major concerns | Some concerns | No concerns | Very low |
| Amitriptyline 100mg:BTX-A 500U | 0 | Some concerns | Low risk | Some concerns | Major concerns | Some concerns | No concerns | Very low |
| Amitriptyline 100mg:BTX-A 50U | 0 | Some concerns | Low risk | Some concerns | Major concerns | Some concerns | No concerns | Very low |
| Amitriptyline 100mg:L-5-Hydroxytryptophan 100mg | 0 | Major concerns | Low risk | Some concerns | Major concerns | Some concerns | No concerns | Very low |
| Amitriptyline 100mg:Lidocaine 25ml | 0 | Some concerns | Low risk | Some concerns | Major concerns | Some concerns | No concerns | Very low |
| Amitriptyline 100mg:Sodium valproate 1g | 0 | Some concerns | Low risk | Some concerns | Major concerns | Some concerns | No concerns | Very low |
| Amitriptyline 75mg:BTX-A 100U | 0 | No concerns | Low risk | Some concerns | Major concerns | Some concerns | No concerns | Very low |
| Amitriptyline 75mg:BTX-A 210U | 0 | No concerns | Low risk | Some concerns | Major concerns | Some concerns | No concerns | Very low |
| Amitriptyline 75mg:BTX-A 420U | 0 | No concerns | Low risk | Some concerns | Major concerns | Some concerns | No concerns | Very low |
| Amitriptyline 75mg:BTX-A 500U | 0 | No concerns | Low risk | Some concerns | Major concerns | Some concerns | No concerns | Very low |
| Amitriptyline 75mg:BTX-A 50U | 0 | No concerns | Low risk | Some concerns | Major concerns | Some concerns | No concerns | Very low |
| Amitriptyline 75mg:L-5-Hydroxytryptophan 100mg | 0 | Some concerns | Low risk | Some concerns | Major concerns | Some concerns | No concerns | Very low |
| Amitriptyline 75mg:Lidocaine 25ml | 0 | No concerns | Low risk | Some concerns | Major concerns | Some concerns | No concerns | Very low |
| Amitriptyline 75mg:Sodium valproate 1g | 0 | No concerns | Low risk | Some concerns | Major concerns | Some concerns | No concerns | Very low |
| Amitriptylinoxide 90mg:BTX-A 100U | 0 | No concerns | Low risk | Some concerns | Major concerns | Some concerns | No concerns | Very low |
| Amitriptylinoxide 90mg:BTX-A 210U | 0 | No concerns | Low risk | Some concerns | Major concerns | Some concerns | No concerns | Very low |
| Amitriptylinoxide 90mg:BTX-A 420U | 0 | No concerns | Low risk | Some concerns | Major concerns | Some concerns | No concerns | Very low |
| Amitriptylinoxide 90mg:BTX-A 500U | 0 | No concerns | Low risk | Some concerns | Major concerns | Some concerns | No concerns | Very low |
| Amitriptylinoxide 90mg:BTX-A 50U | 0 | No concerns | Low risk | Some concerns | Major concerns | Some concerns | No concerns | Very low |
| Amitriptylinoxide 90mg:L-5-Hydroxytryptophan 100mg | 0 | Some concerns | Low risk | Some concerns | Major concerns | Some concerns | No concerns | Very low |
| Amitriptylinoxide 90mg:Lidocaine 25ml | 0 | No concerns | Low risk | Some concerns | Major concerns | Some concerns | No concerns | Very low |
| Amitriptylinoxide 90mg:Sodium valproate 1g | 0 | No concerns | Low risk | Some concerns | Major concerns | Some concerns | No concerns | Very low |
| BTX-A 100U:BTX-A 210U | 0 | No concerns | Low risk | Some concerns | Major concerns | Some concerns | No concerns | Very low |
| BTX-A 100U:BTX-A 420U | 0 | No concerns | Low risk | Some concerns | Major concerns | Some concerns | No concerns | Very low |
| BTX-A 100U:BTX-A 500U | 0 | Some concerns | Low risk | Some concerns | Major concerns | Some concerns | No concerns | Very low |
| BTX-A 100U:BTX-A 50U | 0 | Some concerns | Low risk | Some concerns | Major concerns | Some concerns | No concerns | Very low |
| BTX-A 100U:L-5-Hydroxytryptophan 100mg | 0 | Major concerns | Low risk | Some concerns | Major concerns | Some concerns | No concerns | Very low |
| BTX-A 100U:Lidocaine 25ml | 0 | Some concerns | Low risk | Some concerns | Major concerns | Some concerns | No concerns | Very low |
| BTX-A 100U:Sodium valproate 1g | 0 | Some concerns | Low risk | Some concerns | Major concerns | Some concerns | No concerns | Very low |
| BTX-A 210U:BTX-A 500U | 0 | No concerns | Low risk | Some concerns | Major concerns | Some concerns | No concerns | Very low |
| BTX-A 210U:BTX-A 50U | 0 | No concerns | Low risk | Some concerns | Major concerns | Some concerns | No concerns | Very low |
| BTX-A 210U:L-5-Hydroxytryptophan 100mg | 0 | Some concerns | Low risk | Some concerns | Major concerns | Some concerns | No concerns | Very low |
| BTX-A 210U:Lidocaine 25ml | 0 | No concerns | Low risk | Some concerns | Major concerns | Some concerns | No concerns | Very low |
| BTX-A 210U:Sodium valproate 1g | 0 | No concerns | Low risk | Some concerns | Major concerns | Some concerns | No concerns | Very low |
| BTX-A 420U:BTX-A 500U | 0 | No concerns | Low risk | Some concerns | Major concerns | Some concerns | No concerns | Very low |
| BTX-A 420U:BTX-A 50U | 0 | No concerns | Low risk | Some concerns | Major concerns | Some concerns | No concerns | Very low |
| BTX-A 420U:L-5-Hydroxytryptophan 100mg | 0 | Some concerns | Low risk | Some concerns | Major concerns | Some concerns | No concerns | Very low |
| BTX-A 420U:Lidocaine 25ml | 0 | No concerns | Low risk | Some concerns | Major concerns | Some concerns | No concerns | Very low |
| BTX-A 420U:Sodium valproate 1g | 0 | No concerns | Low risk | Some concerns | Major concerns | Some concerns | No concerns | Very low |
| BTX-A 500U:BTX-A 50U | 0 | No concerns | Low risk | Some concerns | Major concerns | Some concerns | No concerns | Very low |
| BTX-A 500U:L-5-Hydroxytryptophan 100mg | 0 | Some concerns | Low risk | Some concerns | Major concerns | Some concerns | No concerns | Very low |
| BTX-A 500U:Lidocaine 25ml | 0 | Some concerns | Low risk | Some concerns | Major concerns | Some concerns | No concerns | Very low |
| BTX-A 500U:Sodium valproate 1g | 0 | Some concerns | Low risk | Some concerns | Major concerns | Some concerns | No concerns | Very low |
| BTX-A 50U:L-5-Hydroxytryptophan 100mg | 0 | Some concerns | Low risk | Some concerns | Major concerns | Some concerns | No concerns | Very low |
| BTX-A 50U:Lidocaine 25ml | 0 | Some concerns | Low risk | Some concerns | Major concerns | Some concerns | No concerns | Very low |
| BTX-A 50U:Sodium valproate 1g | 0 | Some concerns | Low risk | Some concerns | Major concerns | Some concerns | No concerns | Very low |
| L-5-Hydroxytryptophan 100mg:Lidocaine 25ml | 0 | Major concerns | Low risk | Some concerns | Major concerns | Some concerns | No concerns | Very low |
| L-5-Hydroxytryptophan 100mg:Sodium valproate 1g | 0 | Major concerns | Low risk | Some concerns | Major concerns | Some concerns | No concerns | Very low |
| Lidocaine 25ml:Sodium valproate 1g | 0 | Some concerns | Low risk | Some concerns | Major concerns | Some concerns | No concerns | Very low |
| 24 weeks | | | | | | | | |
| Amitriptyline 100mg:Placebo | 1 | Some concerns | Low risk | No concerns | Some concerns | Some concerns | No concerns | Very low |
| BTX-A 50U:Placebo | 1 | Some concerns | Low risk | No concerns | Some concerns | Some concerns | No concerns | Very low |
| Amitriptyline 100mg:BTX-A 50U | 0 | Some concerns | Low risk | Some concerns | Some concerns | Some concerns | No concerns | Very low |
| Headache intensity | | | | | | | | |
| 4 weeks | | | | | | | | |
| Amitriptyline 100mg:Placebo | 1 | Some concerns | Low risk | No concerns | Some concerns | Some concerns | No concerns | Very low |
| BTX-A 100U:Placebo | 2 | Some concerns | Low risk | No concerns | Some concerns | Some concerns | No concerns | Very low |
| BTX-A 20U:Placebo | 1 | Some concerns | Low risk | No concerns | Some concerns | Some concerns | No concerns | Very low |
| BTX-A 50U:Placebo | 1 | No concerns | Low risk | No concerns | Some concerns | Some concerns | No concerns | Low |
| Placebo:Sodium valproate 1g | 1 | Some concerns | Low risk | No concerns | Some concerns | Some concerns | No concerns | Very low |
| Placebo:Tizanidine 12mg | 1 | Some concerns | Low risk | No concerns | Some concerns | Some concerns | No concerns | Very low |
| Placebo:Tizanidine 6mg | 1 | Some concerns | Low risk | No concerns | Some concerns | Some concerns | No concerns | Very low |
| Tizanidine 12mg:Tizanidine 6mg | 1 | Some concerns | Low risk | No concerns | Some concerns | No concerns | No concerns | Low |
| Amitriptyline 100mg:BTX-A 100U | 0 | Some concerns | Low risk | Some concerns | Some concerns | Some concerns | No concerns | Very low |
| Amitriptyline 100mg:BTX-A 20U | 0 | Some concerns | Low risk | Some concerns | Some concerns | Some concerns | No concerns | Very low |
| Amitriptyline 100mg:BTX-A 50U | 0 | Some concerns | Low risk | Some concerns | Some concerns | Some concerns | No concerns | Very low |
| Amitriptyline 100mg:Sodium valproate 1g | 0 | Some concerns | Low risk | Some concerns | Some concerns | Some concerns | No concerns | Very low |
| Amitriptyline 100mg:Tizanidine 12mg | 0 | Some concerns | Low risk | Some concerns | Some concerns | Some concerns | No concerns | Very low |
| Amitriptyline 100mg:Tizanidine 6mg | 0 | Some concerns | Low risk | Some concerns | Some concerns | Some concerns | No concerns | Very low |
| BTX-A 100U:BTX-A 20U | 0 | Some concerns | Low risk | Some concerns | Some concerns | Some concerns | No concerns | Very low |
| BTX-A 100U:BTX-A 50U | 0 | Some concerns | Low risk | Some concerns | Some concerns | Some concerns | No concerns | Very low |
| BTX-A 100U:Sodium valproate 1g | 0 | Some concerns | Low risk | Some concerns | Some concerns | Some concerns | No concerns | Very low |
| BTX-A 100U:Tizanidine 12mg | 0 | Some concerns | Low risk | Some concerns | Some concerns | Some concerns | No concerns | Very low |
| BTX-A 100U:Tizanidine 6mg | 0 | Some concerns | Low risk | Some concerns | Some concerns | Some concerns | No concerns | Very low |
| BTX-A 20U:BTX-A 50U | 0 | Some concerns | Low risk | Some concerns | Some concerns | Some concerns | No concerns | Very low |
| BTX-A 20U:Sodium valproate 1g | 0 | Some concerns | Low risk | Some concerns | Some concerns | Some concerns | No concerns | Very low |
| BTX-A 20U:Tizanidine 12mg | 0 | Some concerns | Low risk | Some concerns | Some concerns | Some concerns | No concerns | Very low |
| BTX-A 20U:Tizanidine 6mg | 0 | Some concerns | Low risk | Some concerns | Some concerns | Some concerns | No concerns | Very low |
| BTX-A 50U:Sodium valproate 1g | 0 | Some concerns | Low risk | Some concerns | Some concerns | Some concerns | No concerns | Very low |
| BTX-A 50U:Tizanidine 12mg | 0 | Some concerns | Low risk | Some concerns | Some concerns | Some concerns | No concerns | Very low |
| BTX-A 50U:Tizanidine 6mg | 0 | Some concerns | Low risk | Some concerns | Some concerns | Some concerns | No concerns | Very low |
| Sodium valproate 1g:Tizanidine 12mg | 0 | Some concerns | Low risk | Some concerns | Some concerns | Some concerns | No concerns | Very low |
| Sodium valproate 1g:Tizanidine 6mg | 0 | Some concerns | Low risk | Some concerns | Some concerns | Some concerns | No concerns | Very low |
| 8 weeks | | | | | | | | |
| Amitriptyline 100mg:Placebo | 1 | Some concerns | Low risk | No concerns | No concerns | Some concerns | No concerns | Low |
| Amitriptyline 75mg:Citalopram 20mg | 1 | Major concerns | Low risk | No concerns | Major concerns | No concerns | No concerns | Very low |
| Amitriptyline 75mg:Placebo | 1 | Major concerns | Low risk | No concerns | No concerns | No concerns | No concerns | Low |
| BTX-A 100U:BTX-A 150U | 1 | Some concerns | Low risk | No concerns | Major concerns | No concerns | No concerns | Very low |
| BTX-A 100U:BTX-A 50U | 1 | Some concerns | Low risk | No concerns | Major concerns | No concerns | No concerns | Very low |
| BTX-A 100U:BTX-A 86U | 1 | Some concerns | Low risk | No concerns | Major concerns | No concerns | No concerns | Very low |
| BTX-A 100U:Placebo | 3 | Some concerns | Low risk | No concerns | Some concerns | Some concerns | No concerns | Very low |
| BTX-A 150U:BTX-A 50U | 1 | No concerns | Low risk | No concerns | Major concerns | No concerns | No concerns | Low |
| BTX-A 150U:BTX-A 86U | 1 | No concerns | Low risk | No concerns | Major concerns | No concerns | No concerns | Low |
| BTX-A 150U:Placebo | 1 | Some concerns | Low risk | No concerns | No concerns | No concerns | No concerns | Moderate |
| BTX-A 20U:Placebo | 1 | Some concerns | Low risk | No concerns | No concerns | No concerns | No concerns | Moderate |
| BTX-A 50U:BTX-A 86U | 1 | No concerns | Low risk | No concerns | Major concerns | No concerns | No concerns | Low |
| BTX-A 50U:Placebo | 1 | Some concerns | Low risk | No concerns | No concerns | Some concerns | No concerns | Low |
| BTX-A 86U:Placebo | 1 | Some concerns | Low risk | No concerns | No concerns | Some concerns | No concerns | Low |
| Citalopram 20mg:Placebo | 1 | Some concerns | Low risk | No concerns | Some concerns | Some concerns | No concerns | Very low |
| Ibuprofen 400mg:Mitazapine 4.5mg | 1 | Some concerns | Low risk | No concerns | Some concerns | Some concerns | No concerns | Very low |
| Ibuprofen 400mg:Placebo | 1 | Some concerns | Low risk | No concerns | Some concerns | Some concerns | No concerns | Very low |
| L-5-Hydroxytryptophan 100mg:Placebo | 1 | Some concerns | Low risk | No concerns | Some concerns | Some concerns | No concerns | Very low |
| Mitazapine 30mg:Placebo | 1 | Some concerns | Low risk | No concerns | Major concerns | No concerns | No concerns | Very low |
| Mitazapine 4.5mg:Placebo | 1 | Some concerns | Low risk | No concerns | Major concerns | No concerns | No concerns | Very low |
| Placebo:Tizanidine 12mg | 1 | Some concerns | Low risk | No concerns | Major concerns | No concerns | No concerns | Very low |
| Placebo:Tizanidine 6mg | 1 | Some concerns | Low risk | No concerns | Major concerns | No concerns | No concerns | Very low |
| Tizanidine 12mg:Tizanidine 6mg | 1 | Some concerns | Low risk | No concerns | Major concerns | No concerns | No concerns | Very low |
| Amitriptyline 100mg:Amitriptyline 75mg | 0 | Major concerns | Low risk | Some concerns | Major concerns | No concerns | No concerns | Very low |
| Amitriptyline 100mg:BTX-A 100U | 0 | Some concerns | Low risk | Some concerns | Major concerns | No concerns | No concerns | Very low |
| Amitriptyline 100mg:BTX-A 150U | 0 | Some concerns | Low risk | Some concerns | Major concerns | No concerns | No concerns | Very low |
| Amitriptyline 100mg:BTX-A 20U | 0 | Some concerns | Low risk | Some concerns | Major concerns | No concerns | No concerns | Very low |
| Amitriptyline 100mg:BTX-A 50U | 0 | Some concerns | Low risk | Some concerns | Major concerns | No concerns | No concerns | Very low |
| Amitriptyline 100mg:BTX-A 86U | 0 | Some concerns | Low risk | Some concerns | Major concerns | No concerns | No concerns | Very low |
| Amitriptyline 100mg:Citalopram 20mg | 0 | Some concerns | Low risk | Some concerns | Major concerns | No concerns | No concerns | Very low |
| Amitriptyline 100mg:Ibuprofen 400mg | 0 | Some concerns | Low risk | Some concerns | Some concerns | Some concerns | No concerns | Very low |
| Amitriptyline 100mg:L-5-Hydroxytryptophan 100mg | 0 | Some concerns | Low risk | Some concerns | Major concerns | No concerns | No concerns | Very low |
| Amitriptyline 100mg:Mitazapine 30mg | 0 | Some concerns | Low risk | Some concerns | Major concerns | No concerns | No concerns | Very low |
| Amitriptyline 100mg:Mitazapine 4.5mg | 0 | Some concerns | Low risk | Some concerns | Major concerns | No concerns | No concerns | Very low |
| Amitriptyline 100mg:Tizanidine 12mg | 0 | Some concerns | Low risk | Some concerns | Major concerns | No concerns | No concerns | Very low |
| Amitriptyline 100mg:Tizanidine 6mg | 0 | Some concerns | Low risk | Some concerns | Major concerns | No concerns | No concerns | Very low |
| Amitriptyline 75mg:BTX-A 100U | 0 | Some concerns | Low risk | Some concerns | Major concerns | No concerns | No concerns | Very low |
| Amitriptyline 75mg:BTX-A 150U | 0 | Some concerns | Low risk | Some concerns | Major concerns | No concerns | No concerns | Very low |
| Amitriptyline 75mg:BTX-A 20U | 0 | Some concerns | Low risk | Some concerns | Major concerns | No concerns | No concerns | Very low |
| Amitriptyline 75mg:BTX-A 50U | 0 | Some concerns | Low risk | Some concerns | Major concerns | No concerns | No concerns | Very low |
| Amitriptyline 75mg:BTX-A 86U | 0 | Some concerns | Low risk | Some concerns | Major concerns | No concerns | No concerns | Very low |
| Amitriptyline 75mg:Ibuprofen 400mg | 0 | Some concerns | Low risk | Some concerns | Major concerns | No concerns | No concerns | Very low |
| Amitriptyline 75mg:L-5-Hydroxytryptophan 100mg | 0 | Some concerns | Low risk | Some concerns | Major concerns | No concerns | No concerns | Very low |
| Amitriptyline 75mg:Mitazapine 30mg | 0 | Some concerns | Low risk | Some concerns | Major concerns | No concerns | No concerns | Very low |
| Amitriptyline 75mg:Mitazapine 4.5mg | 0 | Some concerns | Low risk | Some concerns | Major concerns | No concerns | No concerns | Very low |
| Amitriptyline 75mg:Tizanidine 12mg | 0 | Some concerns | Low risk | Some concerns | Major concerns | No concerns | No concerns | Very low |
| Amitriptyline 75mg:Tizanidine 6mg | 0 | Some concerns | Low risk | Some concerns | Major concerns | No concerns | No concerns | Very low |
| BTX-A 100U:BTX-A 20U | 0 | Some concerns | Low risk | Some concerns | Some concerns | Some concerns | No concerns | Very low |
| BTX-A 100U:Citalopram 20mg | 0 | Some concerns | Low risk | Some concerns | Major concerns | No concerns | No concerns | Very low |
| BTX-A 100U:Ibuprofen 400mg | 0 | No concerns | Low risk | Some concerns | Some concerns | Some concerns | No concerns | Very low |
| BTX-A 100U:L-5-Hydroxytryptophan 100mg | 0 | Some concerns | Low risk | Some concerns | Major concerns | No concerns | No concerns | Very low |
| BTX-A 100U:Mitazapine 30mg | 0 | Some concerns | Low risk | Some concerns | Major concerns | No concerns | No concerns | Very low |
| BTX-A 100U:Mitazapine 4.5mg | 0 | Some concerns | Low risk | Some concerns | Some concerns | Some concerns | No concerns | Very low |
| BTX-A 100U:Tizanidine 12mg | 0 | Some concerns | Low risk | Some concerns | Major concerns | No concerns | No concerns | Very low |
| BTX-A 100U:Tizanidine 6mg | 0 | Some concerns | Low risk | Some concerns | Major concerns | No concerns | No concerns | Very low |
| BTX-A 150U:BTX-A 20U | 0 | Some concerns | Low risk | Some concerns | Major concerns | No concerns | No concerns | Very low |
| BTX-A 150U:Citalopram 20mg | 0 | Some concerns | Low risk | Some concerns | Major concerns | No concerns | No concerns | Very low |
| BTX-A 150U:Ibuprofen 400mg | 0 | No concerns | Low risk | Some concerns | Some concerns | Some concerns | No concerns | Very low |
| BTX-A 150U:L-5-Hydroxytryptophan 100mg | 0 | Some concerns | Low risk | Some concerns | Major concerns | No concerns | No concerns | Very low |
| BTX-A 150U:Mitazapine 30mg | 0 | No concerns | Low risk | Some concerns | Major concerns | No concerns | No concerns | Very low |
| BTX-A 150U:Mitazapine 4.5mg | 0 | No concerns | Low risk | Some concerns | Major concerns | No concerns | No concerns | Very low |
| BTX-A 150U:Tizanidine 12mg | 0 | Some concerns | Low risk | Some concerns | Major concerns | No concerns | No concerns | Very low |
| BTX-A 150U:Tizanidine 6mg | 0 | Some concerns | Low risk | Some concerns | Major concerns | No concerns | No concerns | Very low |
| BTX-A 20U:BTX-A 50U | 0 | No concerns | Low risk | Some concerns | Major concerns | No concerns | No concerns | Very low |
| BTX-A 20U:BTX-A 86U | 0 | No concerns | Low risk | Some concerns | Major concerns | No concerns | No concerns | Very low |
| BTX-A 20U:Citalopram 20mg | 0 | Major concerns | Low risk | Some concerns | Major concerns | No concerns | No concerns | Very low |
| BTX-A 20U:Ibuprofen 400mg | 0 | No concerns | Low risk | Some concerns | Major concerns | No concerns | No concerns | Very low |
| BTX-A 20U:L-5-Hydroxytryptophan 100mg | 0 | Some concerns | Low risk | Some concerns | Some concerns | Some concerns | No concerns | Very low |
| BTX-A 20U:Mitazapine 30mg | 0 | Some concerns | Low risk | Some concerns | Major concerns | No concerns | No concerns | Very low |
| BTX-A 20U:Mitazapine 4.5mg | 0 | No concerns | Low risk | Some concerns | Major concerns | No concerns | No concerns | Very low |
| BTX-A 20U:Tizanidine 12mg | 0 | Some concerns | Low risk | Some concerns | Major concerns | No concerns | No concerns | Very low |
| BTX-A 20U:Tizanidine 6mg | 0 | Some concerns | Low risk | Some concerns | Major concerns | No concerns | No concerns | Very low |
| BTX-A 50U:Citalopram 20mg | 0 | Some concerns | Low risk | Some concerns | Major concerns | No concerns | No concerns | Very low |
| BTX-A 50U:Ibuprofen 400mg | 0 | No concerns | Low risk | Some concerns | Some concerns | Some concerns | No concerns | Very low |
| BTX-A 50U:L-5-Hydroxytryptophan 100mg | 0 | No concerns | Low risk | Some concerns | Major concerns | No concerns | No concerns | Very low |
| BTX-A 50U:Mitazapine 30mg | 0 | No concerns | Low risk | Some concerns | Major concerns | No concerns | No concerns | Very low |
| BTX-A 50U:Mitazapine 4.5mg | 0 | No concerns | Low risk | Some concerns | Major concerns | No concerns | No concerns | Very low |
| BTX-A 50U:Tizanidine 12mg | 0 | Some concerns | Low risk | Some concerns | Major concerns | No concerns | No concerns | Very low |
| BTX-A 50U:Tizanidine 6mg | 0 | Some concerns | Low risk | Some concerns | Major concerns | No concerns | No concerns | Very low |
| BTX-A 86U:Citalopram 20mg | 0 | Some concerns | Low risk | Some concerns | Major concerns | No concerns | No concerns | Very low |
| BTX-A 86U:Ibuprofen 400mg | 0 | No concerns | Low risk | Some concerns | Some concerns | Some concerns | No concerns | Very low |
| BTX-A 86U:L-5-Hydroxytryptophan 100mg | 0 | No concerns | Low risk | Some concerns | Major concerns | No concerns | No concerns | Very low |
| BTX-A 86U:Mitazapine 30mg | 0 | No concerns | Low risk | Some concerns | Major concerns | No concerns | No concerns | Very low |
| BTX-A 86U:Mitazapine 4.5mg | 0 | No concerns | Low risk | Some concerns | Major concerns | No concerns | No concerns | Very low |
| BTX-A 86U:Tizanidine 12mg | 0 | Some concerns | Low risk | Some concerns | Major concerns | No concerns | No concerns | Very low |
| BTX-A 86U:Tizanidine 6mg | 0 | Some concerns | Low risk | Some concerns | Major concerns | No concerns | No concerns | Very low |
| Citalopram 20mg:Ibuprofen 400mg | 0 | Some concerns | Low risk | Some concerns | Some concerns | Some concerns | No concerns | Very low |
| Citalopram 20mg:L-5-Hydroxytryptophan 100mg | 0 | Major concerns | Low risk | Some concerns | Major concerns | No concerns | No concerns | Very low |
| Citalopram 20mg:Mitazapine 30mg | 0 | Some concerns | Low risk | Some concerns | Major concerns | No concerns | No concerns | Very low |
| Citalopram 20mg:Mitazapine 4.5mg | 0 | Some concerns | Low risk | Some concerns | Major concerns | No concerns | No concerns | Very low |
| Citalopram 20mg:Tizanidine 12mg | 0 | Some concerns | Low risk | Some concerns | Major concerns | No concerns | No concerns | Very low |
| Citalopram 20mg:Tizanidine 6mg | 0 | Some concerns | Low risk | Some concerns | Major concerns | No concerns | No concerns | Very low |
| Ibuprofen 400mg:L-5-Hydroxytryptophan 100mg | 0 | No concerns | Low risk | Some concerns | Some concerns | Some concerns | No concerns | Very low |
| Ibuprofen 400mg:Mitazapine 30mg | 0 | No concerns | Low risk | Some concerns | Major concerns | No concerns | No concerns | Very low |
| Ibuprofen 400mg:Tizanidine 12mg | 0 | Some concerns | Low risk | Some concerns | Major concerns | No concerns | No concerns | Very low |
| Ibuprofen 400mg:Tizanidine 6mg | 0 | Some concerns | Low risk | Some concerns | Major concerns | No concerns | No concerns | Very low |
| L-5-Hydroxytryptophan 100mg:Mitazapine 30mg | 0 | Some concerns | Low risk | Some concerns | Major concerns | No concerns | No concerns | Very low |
| L-5-Hydroxytryptophan 100mg:Mitazapine 4.5mg | 0 | No concerns | Low risk | Some concerns | Some concerns | Some concerns | No concerns | Very low |
| L-5-Hydroxytryptophan 100mg:Tizanidine 12mg | 0 | Some concerns | Low risk | Some concerns | Major concerns | No concerns | No concerns | Very low |
| L-5-Hydroxytryptophan 100mg:Tizanidine 6mg | 0 | Some concerns | Low risk | Some concerns | Major concerns | No concerns | No concerns | Very low |
| Mitazapine 30mg:Mitazapine 4.5mg | 0 | No concerns | Low risk | Some concerns | Major concerns | No concerns | No concerns | Very low |
| Mitazapine 30mg:Tizanidine 12mg | 0 | Some concerns | Low risk | Some concerns | Major concerns | No concerns | No concerns | Very low |
| Mitazapine 30mg:Tizanidine 6mg | 0 | Some concerns | Low risk | Some concerns | Major concerns | No concerns | No concerns | Very low |
| Mitazapine 4.5mg:Tizanidine 12mg | 0 | Some concerns | Low risk | Some concerns | Major concerns | No concerns | No concerns | Very low |
| Mitazapine 4.5mg:Tizanidine 6mg | 0 | Some concerns | Low risk | Some concerns | Major concerns | No concerns | No concerns | Very low |
| 12 weeks | | | | | | | | |
| Amitriptyline 100mg:Placebo | 1 | Some concerns | Low risk | No concerns | Some concerns | Some concerns | No concerns | Very low |
| Amitriptyline 75mg:Amitriptylinoxide 90mg | 1 | No concerns | Low risk | No concerns | Major concerns | No concerns | No concerns | Low |
| Amitriptyline 75mg:Placebo | 1 | No concerns | Low risk | No concerns | Some concerns | Some concerns | No concerns | Low |
| Amitriptylinoxide 90mg:Placebo | 1 | No concerns | Low risk | No concerns | Some concerns | Some concerns | No concerns | Low |
| BTX-A 100U:Placebo | 3 | Some concerns | Low risk | No concerns | Some concerns | Some concerns | No concerns | Very low |
| BTX-A 210U:BTX-A 420U | 1 | No concerns | Low risk | No concerns | Major concerns | No concerns | No concerns | Low |
| BTX-A 210U:Placebo | 1 | No concerns | Low risk | No concerns | Major concerns | No concerns | No concerns | Low |
| BTX-A 420U:Placebo | 1 | No concerns | Low risk | No concerns | Major concerns | No concerns | No concerns | Low |
| BTX-A 50U:Placebo | 1 | No concerns | Low risk | No concerns | Some concerns | Some concerns | No concerns | Low |
| L-5-Hydroxytryptophan 100mg:Placebo | 1 | Some concerns | Low risk | No concerns | Some concerns | Some concerns | No concerns | Very low |
| Lidocaine 25ml:Placebo | 1 | Some concerns | Low risk | No concerns | No concerns | Major concerns | No concerns | Very low |
| Placebo:Sodium valproate 1g | 1 | Some concerns | Low risk | No concerns | Some concerns | Some concerns | No concerns | Very low |
| Amitriptyline 100mg:Amitriptyline 75mg | 0 | No concerns | Low risk | Some concerns | Some concerns | Some concerns | No concerns | Very low |
| Amitriptyline 100mg:Amitriptylinoxide 90mg | 0 | No concerns | Low risk | Some concerns | Some concerns | Some concerns | No concerns | Very low |
| Amitriptyline 100mg:BTX-A 100U | 0 | Some concerns | Low risk | Some concerns | Major concerns | No concerns | No concerns | Very low |
| Amitriptyline 100mg:BTX-A 210U | 0 | No concerns | Low risk | Some concerns | Major concerns | No concerns | No concerns | Very low |
| Amitriptyline 100mg:BTX-A 420U | 0 | No concerns | Low risk | Some concerns | Major concerns | No concerns | No concerns | Very low |
| Amitriptyline 100mg:BTX-A 50U | 0 | Some concerns | Low risk | Some concerns | Major concerns | No concerns | No concerns | Very low |
| Amitriptyline 100mg:L-5-Hydroxytryptophan 100mg | 0 | Some concerns | Low risk | Some concerns | Major concerns | No concerns | No concerns | Very low |
| Amitriptyline 100mg:Lidocaine 25ml | 0 | Some concerns | Low risk | Some concerns | No concerns | Major concerns | No concerns | Very low |
| Amitriptyline 100mg:Sodium valproate 1g | 0 | Some concerns | Low risk | Some concerns | Major concerns | No concerns | No concerns | Very low |
| Amitriptyline 75mg:BTX-A 100U | 0 | No concerns | Low risk | Some concerns | No concerns | Major concerns | No concerns | Very low |
| Amitriptyline 75mg:BTX-A 210U | 0 | No concerns | Low risk | Some concerns | Some concerns | Some concerns | No concerns | Very low |
| Amitriptyline 75mg:BTX-A 420U | 0 | No concerns | Low risk | Some concerns | Some concerns | Some concerns | No concerns | Very low |
| Amitriptyline 75mg:BTX-A 50U | 0 | No concerns | Low risk | Some concerns | No concerns | Major concerns | No concerns | Very low |
| Amitriptyline 75mg:L-5-Hydroxytryptophan 100mg | 0 | No concerns | Low risk | Some concerns | Some concerns | Some concerns | No concerns | Very low |
| Amitriptyline 75mg:Lidocaine 25ml | 0 | No concerns | Low risk | Some concerns | No concerns | Major concerns | No concerns | Very low |
| Amitriptyline 75mg:Sodium valproate 1g | 0 | No concerns | Low risk | Some concerns | Some concerns | Some concerns | No concerns | Very low |
| Amitriptylinoxide 90mg:BTX-A 100U | 0 | No concerns | Low risk | Some concerns | No concerns | Major concerns | No concerns | Very low |
| Amitriptylinoxide 90mg:BTX-A 210U | 0 | No concerns | Low risk | Some concerns | Some concerns | Some concerns | No concerns | Very low |
| Amitriptylinoxide 90mg:BTX-A 420U | 0 | No concerns | Low risk | Some concerns | Some concerns | Some concerns | No concerns | Very low |
| Amitriptylinoxide 90mg:BTX-A 50U | 0 | No concerns | Low risk | Some concerns | No concerns | Major concerns | No concerns | Very low |
| Amitriptylinoxide 90mg:L-5-Hydroxytryptophan 100mg | 0 | No concerns | Low risk | Some concerns | Some concerns | Some concerns | No concerns | Very low |
| Amitriptylinoxide 90mg:Lidocaine 25ml | 0 | No concerns | Low risk | Some concerns | No concerns | Major concerns | No concerns | Very low |
| Amitriptylinoxide 90mg:Sodium valproate 1g | 0 | No concerns | Low risk | Some concerns | Some concerns | Some concerns | No concerns | Very low |
| BTX-A 100U:BTX-A 210U | 0 | No concerns | Low risk | Some concerns | Major concerns | No concerns | No concerns | Very low |
| BTX-A 100U:BTX-A 420U | 0 | No concerns | Low risk | Some concerns | Major concerns | No concerns | No concerns | Very low |
| BTX-A 100U:BTX-A 50U | 0 | Some concerns | Low risk | Some concerns | Major concerns | No concerns | No concerns | Very low |
| BTX-A 100U:L-5-Hydroxytryptophan 100mg | 0 | Some concerns | Low risk | Some concerns | Major concerns | No concerns | No concerns | Very low |
| BTX-A 100U:Lidocaine 25ml | 0 | Some concerns | Low risk | Some concerns | No concerns | Major concerns | No concerns | Very low |
| BTX-A 100U:Sodium valproate 1g | 0 | Some concerns | Low risk | Some concerns | Major concerns | No concerns | No concerns | Very low |
| BTX-A 210U:BTX-A 50U | 0 | No concerns | Low risk | Some concerns | Some concerns | Some concerns | No concerns | Very low |
| BTX-A 210U:L-5-Hydroxytryptophan 100mg | 0 | No concerns | Low risk | Some concerns | Major concerns | No concerns | No concerns | Very low |
| BTX-A 210U:Lidocaine 25ml | 0 | No concerns | Low risk | Some concerns | No concerns | Major concerns | No concerns | Very low |
| BTX-A 210U:Sodium valproate 1g | 0 | No concerns | Low risk | Some concerns | Major concerns | No concerns | No concerns | Very low |
| BTX-A 420U:BTX-A 50U | 0 | No concerns | Low risk | Some concerns | Some concerns | Some concerns | No concerns | Very low |
| BTX-A 420U:L-5-Hydroxytryptophan 100mg | 0 | No concerns | Low risk | Some concerns | Major concerns | No concerns | No concerns | Very low |
| BTX-A 420U:Lidocaine 25ml | 0 | No concerns | Low risk | Some concerns | No concerns | Major concerns | No concerns | Very low |
| BTX-A 420U:Sodium valproate 1g | 0 | No concerns | Low risk | Some concerns | Major concerns | No concerns | No concerns | Very low |
| BTX-A 50U:L-5-Hydroxytryptophan 100mg | 0 | Some concerns | Low risk | Some concerns | Major concerns | No concerns | No concerns | Very low |
| BTX-A 50U:Lidocaine 25ml | 0 | Some concerns | Low risk | Some concerns | Some concerns | Some concerns | No concerns | Very low |
| BTX-A 50U:Sodium valproate 1g | 0 | Some concerns | Low risk | Some concerns | Major concerns | No concerns | No concerns | Very low |
| L-5-Hydroxytryptophan 100mg:Lidocaine 25ml | 0 | Some concerns | Low risk | Some concerns | No concerns | Major concerns | No concerns | Very low |
| L-5-Hydroxytryptophan 100mg:Sodium valproate 1g | 0 | Some concerns | Low risk | Some concerns | Major concerns | No concerns | No concerns | Very low |
| Lidocaine 25ml:Sodium valproate 1g | 0 | Some concerns | Low risk | Some concerns | No concerns | Major concerns | No concerns | Very low |
| 24 weeks | | | | | | | | |
| Amitriptyline 100mg:Placebo | 1 | Some concerns | Low risk | No concerns | Some concerns | Some concerns | No concerns | Very low |
| BTX-A 50U:Placebo | 1 | Some concerns | Low risk | No concerns | Some concerns | Some concerns | No concerns | Very low |
| Amitriptyline 100mg:BTX-A 50U | 0 | Some concerns | Low risk | Some concerns | Some concerns | Some concerns | No concerns | Very low |
| Headache duration | | | | | | | | |
| 4 weeks | | | | | | | | |
| BTX-A 210U:BTX-A 420U | 1 | No concerns | Low risk | No concerns | Major concerns | No concerns | No concerns | Low |
| BTX-A 210U:Placebo | 1 | Some concerns | Low risk | No concerns | Some concerns | Some concerns | No concerns | Very low |
| BTX-A 420U:Placebo | 1 | Some concerns | Low risk | No concerns | Some concerns | Some concerns | No concerns | Very low |
| BTX-A 50U:Placebo | 1 | Some concerns | Low risk | No concerns | Some concerns | Some concerns | No concerns | Very low |
| Placebo:Tizanidine 12mg | 1 | Some concerns | Low risk | No concerns | Some concerns | Some concerns | No concerns | Very low |
| Placebo:Tizanidine 6mg | 1 | Some concerns | Low risk | No concerns | Some concerns | Some concerns | No concerns | Very low |
| Tizanidine 12mg:Tizanidine 6mg | 1 | Some concerns | Low risk | No concerns | Some concerns | Some concerns | No concerns | Very low |
| BTX-A 210U:BTX-A 50U | 0 | No concerns | Low risk | Some concerns | Some concerns | Some concerns | No concerns | Very low |
| BTX-A 210U:Tizanidine 12mg | 0 | Some concerns | Low risk | Some concerns | Some concerns | Some concerns | No concerns | Very low |
| BTX-A 210U:Tizanidine 6mg | 0 | Some concerns | Low risk | Some concerns | Some concerns | Some concerns | No concerns | Very low |
| BTX-A 420U:BTX-A 50U | 0 | No concerns | Low risk | Some concerns | Some concerns | Some concerns | No concerns | Very low |
| BTX-A 420U:Tizanidine 12mg | 0 | Some concerns | Low risk | Some concerns | Some concerns | Some concerns | No concerns | Very low |
| BTX-A 420U:Tizanidine 6mg | 0 | Some concerns | Low risk | Some concerns | Some concerns | Some concerns | No concerns | Very low |
| BTX-A 50U:Tizanidine 12mg | 0 | Some concerns | Low risk | Some concerns | Some concerns | Some concerns | No concerns | Very low |
| BTX-A 50U:Tizanidine 6mg | 0 | Some concerns | Low risk | Some concerns | Some concerns | Some concerns | No concerns | Very low |
| 8 weeks | | | | | | | | |
| Amitriptyline 75mg:Citalopram 20mg | 1 | Major concerns | Low risk | No concerns | Some concerns | No concerns | No concerns | Very low |
| Amitriptyline 75mg:Placebo | 1 | Some concerns | Low risk | No concerns | Some concerns | Some concerns | No concerns | Very low |
| BTX-A 210U:BTX-A 420U | 1 | No concerns | Low risk | No concerns | Some concerns | No concerns | No concerns | Moderate |
| BTX-A 210U:Placebo | 1 | Some concerns | Low risk | No concerns | Some concerns | Some concerns | No concerns | Very low |
| BTX-A 420U:Placebo | 1 | Some concerns | Low risk | No concerns | Some concerns | Some concerns | No concerns | Very low |
| Citalopram 20mg:Placebo | 1 | Some concerns | Low risk | No concerns | Some concerns | Some concerns | No concerns | Very low |
| Ibuprofen 400mg:Mitazapine 4.5mg | 1 | No concerns | Low risk | No concerns | Some concerns | No concerns | No concerns | Moderate |
| Ibuprofen 400mg:Placebo | 1 | Some concerns | Low risk | No concerns | Some concerns | Some concerns | No concerns | Very low |
| Mitazapine 30mg:Placebo | 1 | Some concerns | Low risk | No concerns | Some concerns | Some concerns | No concerns | Very low |
| Mitazapine 4.5mg:Placebo | 1 | Some concerns | Low risk | No concerns | Some concerns | Some concerns | No concerns | Very low |
| Placebo:Tizanidine 12mg | 1 | Some concerns | Low risk | No concerns | Some concerns | Some concerns | No concerns | Very low |
| Placebo:Tizanidine 6mg | 1 | Some concerns | Low risk | No concerns | Some concerns | Some concerns | No concerns | Very low |
| Tizanidine 12mg:Tizanidine 6mg | 1 | Some concerns | Low risk | No concerns | Some concerns | No concerns | No concerns | Low |
| Amitriptyline 75mg:BTX-A 210U | 0 | Some concerns | Low risk | Some concerns | Some concerns | Some concerns | No concerns | Very low |
| Amitriptyline 75mg:BTX-A 420U | 0 | Some concerns | Low risk | Some concerns | Some concerns | Some concerns | No concerns | Very low |
| Amitriptyline 75mg:Ibuprofen 400mg | 0 | Some concerns | Low risk | Some concerns | Some concerns | Some concerns | No concerns | Very low |
| Amitriptyline 75mg:Mitazapine 30mg | 0 | Some concerns | Low risk | Some concerns | Some concerns | Some concerns | No concerns | Very low |
| Amitriptyline 75mg:Mitazapine 4.5mg | 0 | Some concerns | Low risk | Some concerns | Some concerns | Some concerns | No concerns | Very low |
| Amitriptyline 75mg:Tizanidine 12mg | 0 | Some concerns | Low risk | Some concerns | Some concerns | Some concerns | No concerns | Very low |
| Amitriptyline 75mg:Tizanidine 6mg | 0 | Some concerns | Low risk | Some concerns | Some concerns | Some concerns | No concerns | Very low |
| BTX-A 210U:Citalopram 20mg | 0 | Some concerns | Low risk | Some concerns | Some concerns | Some concerns | No concerns | Very low |
| BTX-A 210U:Ibuprofen 400mg | 0 | Some concerns | Low risk | Some concerns | Some concerns | Some concerns | No concerns | Very low |
| BTX-A 210U:Mitazapine 30mg | 0 | Some concerns | Low risk | Some concerns | Some concerns | Some concerns | No concerns | Very low |
| BTX-A 210U:Mitazapine 4.5mg | 0 | Some concerns | Low risk | Some concerns | Some concerns | Some concerns | No concerns | Very low |
| BTX-A 210U:Tizanidine 12mg | 0 | Some concerns | Low risk | Some concerns | Some concerns | Some concerns | No concerns | Very low |
| BTX-A 210U:Tizanidine 6mg | 0 | Some concerns | Low risk | Some concerns | Some concerns | Some concerns | No concerns | Very low |
| BTX-A 420U:Citalopram 20mg | 0 | Some concerns | Low risk | Some concerns | Some concerns | Some concerns | No concerns | Very low |
| BTX-A 420U:Ibuprofen 400mg | 0 | Some concerns | Low risk | Some concerns | Some concerns | Some concerns | No concerns | Very low |
| BTX-A 420U:Mitazapine 30mg | 0 | Some concerns | Low risk | Some concerns | Some concerns | Some concerns | No concerns | Very low |
| BTX-A 420U:Mitazapine 4.5mg | 0 | Some concerns | Low risk | Some concerns | Some concerns | Some concerns | No concerns | Very low |
| BTX-A 420U:Tizanidine 12mg | 0 | Some concerns | Low risk | Some concerns | Some concerns | Some concerns | No concerns | Very low |
| BTX-A 420U:Tizanidine 6mg | 0 | Some concerns | Low risk | Some concerns | Some concerns | Some concerns | No concerns | Very low |
| Citalopram 20mg:Ibuprofen 400mg | 0 | Some concerns | Low risk | Some concerns | Some concerns | Some concerns | No concerns | Very low |
| Citalopram 20mg:Mitazapine 30mg | 0 | Some concerns | Low risk | Some concerns | Some concerns | Some concerns | No concerns | Very low |
| Citalopram 20mg:Mitazapine 4.5mg | 0 | Some concerns | Low risk | Some concerns | Some concerns | Some concerns | No concerns | Very low |
| Citalopram 20mg:Tizanidine 12mg | 0 | Some concerns | Low risk | Some concerns | Some concerns | Some concerns | No concerns | Very low |
| Citalopram 20mg:Tizanidine 6mg | 0 | Some concerns | Low risk | Some concerns | Some concerns | Some concerns | No concerns | Very low |
| Ibuprofen 400mg:Mitazapine 30mg | 0 | Some concerns | Low risk | Some concerns | Some concerns | Some concerns | No concerns | Very low |
| Ibuprofen 400mg:Tizanidine 12mg | 0 | Some concerns | Low risk | Some concerns | Some concerns | Some concerns | No concerns | Very low |
| Ibuprofen 400mg:Tizanidine 6mg | 0 | Some concerns | Low risk | Some concerns | Some concerns | Some concerns | No concerns | Very low |
| Mitazapine 30mg:Mitazapine 4.5mg | 0 | Some concerns | Low risk | Some concerns | Some concerns | Some concerns | No concerns | Very low |
| Mitazapine 30mg:Tizanidine 12mg | 0 | Some concerns | Low risk | Some concerns | Some concerns | Some concerns | No concerns | Very low |
| Mitazapine 30mg:Tizanidine 6mg | 0 | Some concerns | Low risk | Some concerns | Some concerns | Some concerns | No concerns | Very low |
| Mitazapine 4.5mg:Tizanidine 12mg | 0 | Some concerns | Low risk | Some concerns | Some concerns | Some concerns | No concerns | Very low |
| Mitazapine 4.5mg:Tizanidine 6mg | 0 | Some concerns | Low risk | Some concerns | Some concerns | Some concerns | No concerns | Very low |
| 12 weeks | | | | | | | | |
| Amitriptyline 75mg:Amitriptylinoxide 90mg | 1 | No concerns | Low risk | No concerns | Some concerns | No concerns | No concerns | Moderate |
| Amitriptyline 75mg:Placebo | 1 | Some concerns | Low risk | No concerns | Some concerns | Some concerns | No concerns | Very low |
| Amitriptylinoxide 90mg:Placebo | 1 | Some concerns | Low risk | No concerns | Some concerns | Some concerns | No concerns | Very low |
| BTX-A 100U:Placebo | 1 | Some concerns | Low risk | No concerns | Some concerns | Some concerns | No concerns | Very low |
| BTX-A 210U:BTX-A 420U | 1 | No concerns | Low risk | No concerns | Some concerns | No concerns | No concerns | Moderate |
| BTX-A 210U:Placebo | 1 | Some concerns | Low risk | No concerns | Some concerns | Some concerns | No concerns | Very low |
| BTX-A 420U:Placebo | 1 | Some concerns | Low risk | No concerns | Some concerns | Some concerns | No concerns | Very low |
| BTX-A 50U:Placebo | 1 | Some concerns | Low risk | No concerns | Some concerns | Some concerns | No concerns | Very low |
| Amitriptyline 75mg:BTX-A 100U | 0 | Some concerns | Low risk | Some concerns | Some concerns | Some concerns | No concerns | Very low |
| Amitriptyline 75mg:BTX-A 210U | 0 | No concerns | Low risk | Some concerns | Some concerns | Some concerns | No concerns | Very low |
| Amitriptyline 75mg:BTX-A 420U | 0 | No concerns | Low risk | Some concerns | Some concerns | Some concerns | No concerns | Very low |
| Amitriptyline 75mg:BTX-A 50U | 0 | No concerns | Low risk | Some concerns | Some concerns | Some concerns | No concerns | Very low |
| Amitriptylinoxide 90mg:BTX-A 100U | 0 | Some concerns | Low risk | Some concerns | Some concerns | Some concerns | No concerns | Very low |
| Amitriptylinoxide 90mg:BTX-A 210U | 0 | No concerns | Low risk | Some concerns | Some concerns | Some concerns | No concerns | Very low |
| Amitriptylinoxide 90mg:BTX-A 420U | 0 | No concerns | Low risk | Some concerns | Some concerns | Some concerns | No concerns | Very low |
| Amitriptylinoxide 90mg:BTX-A 50U | 0 | No concerns | Low risk | Some concerns | Some concerns | Some concerns | No concerns | Very low |
| BTX-A 100U:BTX-A 210U | 0 | Some concerns | Low risk | Some concerns | Some concerns | Some concerns | No concerns | Very low |
| BTX-A 100U:BTX-A 420U | 0 | Some concerns | Low risk | Some concerns | Some concerns | Some concerns | No concerns | Very low |
| BTX-A 100U:BTX-A 50U | 0 | Some concerns | Low risk | Some concerns | Some concerns | Some concerns | No concerns | Very low |
| BTX-A 210U:BTX-A 50U | 0 | Major concerns | Low risk | Some concerns | Some concerns | Some concerns | No concerns | Very low |
| Adverse event rate | | | | | | | | |
| Amitriptyline 100mg:Placebo | 1 | Some concerns | Low risk | No concerns | No concerns | Some concerns | No concerns | Low |
| Amitriptyline 25mg:Placebo | 1 | Major concerns | Low risk | No concerns | No concerns | Some concerns | No concerns | Very low |
| Amitriptyline 75mg:Amitriptylinoxide 90mg | 1 | No concerns | Low risk | No concerns | Some concerns | Some concerns | No concerns | Low |
| Amitriptyline 75mg:Citalopram 20mg | 1 | Major concerns | Low risk | No concerns | Some concerns | Some concerns | No concerns | Very low |
| Amitriptyline 75mg:Placebo | 2 | Some concerns | Low risk | No concerns | No concerns | Some concerns | No concerns | Low |
| Amitriptylinoxide 90mg:Placebo | 1 | Some concerns | Low risk | No concerns | No concerns | Major concerns | No concerns | Very low |
| BTX-A 100U:BTX-A 150U | 1 | Some concerns | Low risk | No concerns | Some concerns | Major concerns | No concerns | Very low |
| BTX-A 100U:BTX-A 50U | 1 | Some concerns | Low risk | No concerns | Some concerns | Major concerns | No concerns | Very low |
| BTX-A 100U:BTX-A 86U | 1 | Some concerns | Low risk | No concerns | Some concerns | Major concerns | No concerns | Very low |
| BTX-A 100U:Placebo | 3 | Some concerns | Low risk | No concerns | No concerns | Some concerns | No concerns | Low |
| BTX-A 150U:BTX-A 50U | 1 | No concerns | Low risk | No concerns | Some concerns | Major concerns | No concerns | Very low |
| BTX-A 150U:BTX-A 86U | 1 | No concerns | Low risk | No concerns | Some concerns | Major concerns | No concerns | Very low |
| BTX-A 150U:Placebo | 1 | Some concerns | Low risk | No concerns | No concerns | Major concerns | No concerns | Very low |
| BTX-A 20U:Placebo | 1 | Some concerns | Low risk | No concerns | No concerns | Some concerns | No concerns | Low |
| BTX-A 210U:BTX-A 420U | 1 | No concerns | Low risk | No concerns | Some concerns | Major concerns | No concerns | Very low |
| BTX-A 210U:Placebo | 1 | Some concerns | Low risk | No concerns | No concerns | Some concerns | No concerns | Low |
| BTX-A 420U:Placebo | 1 | Some concerns | Low risk | No concerns | No concerns | Some concerns | No concerns | Low |
| BTX-A 500U:Placebo | 1 | Some concerns | Low risk | No concerns | No concerns | Some concerns | No concerns | Low |
| BTX-A 50U:BTX-A 86U | 1 | No concerns | Low risk | No concerns | Some concerns | Major concerns | No concerns | Very low |
| BTX-A 50U:Placebo | 3 | Some concerns | Low risk | No concerns | No concerns | Some concerns | No concerns | Low |
| BTX-A 86U:Placebo | 1 | Some concerns | Low risk | No concerns | No concerns | Major concerns | No concerns | Very low |
| Citalopram 20mg:Placebo | 1 | Major concerns | Low risk | No concerns | No concerns | Major concerns | No concerns | Very low |
| Ibuprofen 400mg:Mitazapine 4.5mg | 1 | No concerns | Low risk | No concerns | Some concerns | Some concerns | No concerns | Low |
| Ibuprofen 400mg:Placebo | 1 | Some concerns | Low risk | No concerns | No concerns | Major concerns | No concerns | Very low |
| L-5-Hydroxytryptophan 100mg:Placebo | 1 | Major concerns | Low risk | No concerns | Some concerns | Some concerns | No concerns | Very low |
| Lidocaine 25ml:Placebo | 1 | Some concerns | Low risk | No concerns | No concerns | Major concerns | No concerns | Very low |
| Mitazapine 30mg:Placebo | 1 | Some concerns | Low risk | No concerns | No concerns | Major concerns | No concerns | Very low |
| Mitazapine 4.5mg:Placebo | 1 | Some concerns | Low risk | No concerns | No concerns | Major concerns | No concerns | Very low |
| Placebo:Sodium valproate 1g | 1 | Some concerns | Low risk | No concerns | Some concerns | Some concerns | No concerns | Very low |
| Amitriptyline 100mg:Amitriptyline 25mg | 0 | Major concerns | Low risk | Some concerns | Some concerns | Some concerns | No concerns | Very low |
| Amitriptyline 100mg:Amitriptyline 75mg | 0 | Some concerns | Low risk | Some concerns | Some concerns | No concerns | No concerns | Very low |
| Amitriptyline 100mg:Amitriptylinoxide 90mg | 0 | Some concerns | Low risk | Some concerns | Some concerns | No concerns | No concerns | Very low |
| Amitriptyline 100mg:BTX-A 100U | 0 | Some concerns | Low risk | Some concerns | Some concerns | Some concerns | No concerns | Very low |
| Amitriptyline 100mg:BTX-A 150U | 0 | Some concerns | Low risk | Some concerns | Some concerns | Some concerns | No concerns | Very low |
| Amitriptyline 100mg:BTX-A 20U | 0 | Some concerns | Low risk | Some concerns | Some concerns | No concerns | No concerns | Very low |
| Amitriptyline 100mg:BTX-A 210U | 0 | Some concerns | Low risk | Some concerns | Some concerns | Some concerns | No concerns | Very low |
| Amitriptyline 100mg:BTX-A 420U | 0 | Some concerns | Low risk | Some concerns | Some concerns | Some concerns | No concerns | Very low |
| Amitriptyline 100mg:BTX-A 500U | 0 | Some concerns | Low risk | Some concerns | Major concerns | No concerns | No concerns | Very low |
| Amitriptyline 100mg:BTX-A 50U | 0 | Some concerns | Low risk | Some concerns | Some concerns | Some concerns | No concerns | Very low |
| Amitriptyline 100mg:BTX-A 86U | 0 | Some concerns | Low risk | Some concerns | Some concerns | Some concerns | No concerns | Very low |
| Amitriptyline 100mg:Citalopram 20mg | 0 | Some concerns | Low risk | Some concerns | Some concerns | Some concerns | No concerns | Very low |
| Amitriptyline 100mg:Ibuprofen 400mg | 0 | Some concerns | Low risk | Some concerns | Some concerns | No concerns | No concerns | Very low |
| Amitriptyline 100mg:L-5-Hydroxytryptophan 100mg | 0 | Major concerns | Low risk | Some concerns | Some concerns | No concerns | No concerns | Very low |
| Amitriptyline 100mg:Lidocaine 25ml | 0 | Some concerns | Low risk | Some concerns | Some concerns | Some concerns | No concerns | Very low |
| Amitriptyline 100mg:Mitazapine 30mg | 0 | Some concerns | Low risk | Some concerns | Major concerns | No concerns | No concerns | Very low |
| Amitriptyline 100mg:Mitazapine 4.5mg | 0 | Some concerns | Low risk | Some concerns | Some concerns | Some concerns | No concerns | Very low |
| Amitriptyline 100mg:Sodium valproate 1g | 0 | Some concerns | Low risk | Some concerns | Some concerns | Some concerns | No concerns | Very low |
| Amitriptyline 25mg:Amitriptyline 75mg | 0 | Some concerns | Low risk | Some concerns | Some concerns | Some concerns | No concerns | Very low |
| Amitriptyline 25mg:Amitriptylinoxide 90mg | 0 | Some concerns | Low risk | Some concerns | Some concerns | Some concerns | No concerns | Very low |
| Amitriptyline 25mg:BTX-A 100U | 0 | Some concerns | Low risk | Some concerns | Some concerns | Some concerns | No concerns | Very low |
| Amitriptyline 25mg:BTX-A 150U | 0 | Some concerns | Low risk | Some concerns | Some concerns | Some concerns | No concerns | Very low |
| Amitriptyline 25mg:BTX-A 20U | 0 | Major concerns | Low risk | Some concerns | Major concerns | No concerns | No concerns | Very low |
| Amitriptyline 25mg:BTX-A 210U | 0 | Some concerns | Low risk | Some concerns | Some concerns | Some concerns | No concerns | Very low |
| Amitriptyline 25mg:BTX-A 420U | 0 | Some concerns | Low risk | Some concerns | Some concerns | Some concerns | No concerns | Very low |
| Amitriptyline 25mg:BTX-A 500U | 0 | Some concerns | Low risk | Some concerns | Some concerns | Some concerns | No concerns | Very low |
| Amitriptyline 25mg:BTX-A 50U | 0 | Some concerns | Low risk | Some concerns | Some concerns | Some concerns | No concerns | Very low |
| Amitriptyline 25mg:BTX-A 86U | 0 | Some concerns | Low risk | Some concerns | Some concerns | Some concerns | No concerns | Very low |
| Amitriptyline 25mg:Citalopram 20mg | 0 | Major concerns | Low risk | Some concerns | Some concerns | Some concerns | No concerns | Very low |
| Amitriptyline 25mg:Ibuprofen 400mg | 0 | Some concerns | Low risk | Some concerns | Some concerns | Some concerns | No concerns | Very low |
| Amitriptyline 25mg:L-5-Hydroxytryptophan 100mg | 0 | Major concerns | Low risk | Some concerns | Major concerns | No concerns | No concerns | Very low |
| Amitriptyline 25mg:Lidocaine 25ml | 0 | Major concerns | Low risk | Some concerns | Some concerns | Some concerns | No concerns | Very low |
| Amitriptyline 25mg:Mitazapine 30mg | 0 | Some concerns | Low risk | Some concerns | Some concerns | Some concerns | No concerns | Very low |
| Amitriptyline 25mg:Mitazapine 4.5mg | 0 | Some concerns | Low risk | Some concerns | Major concerns | No concerns | No concerns | Very low |
| Amitriptyline 25mg:Sodium valproate 1g | 0 | Major concerns | Low risk | Some concerns | Major concerns | No concerns | No concerns | Very low |
| Amitriptyline 75mg:BTX-A 100U | 0 | Some concerns | Low risk | Some concerns | Some concerns | No concerns | No concerns | Very low |
| Amitriptyline 75mg:BTX-A 150U | 0 | Some concerns | Low risk | Some concerns | Some concerns | No concerns | No concerns | Very low |
| Amitriptyline 75mg:BTX-A 20U | 0 | Some concerns | Low risk | Some concerns | Some concerns | Some concerns | No concerns | Very low |
| Amitriptyline 75mg:BTX-A 210U | 0 | Some concerns | Low risk | Some concerns | Some concerns | Some concerns | No concerns | Very low |
| Amitriptyline 75mg:BTX-A 420U | 0 | Some concerns | Low risk | Some concerns | Some concerns | Some concerns | No concerns | Very low |
| Amitriptyline 75mg:BTX-A 500U | 0 | Some concerns | Low risk | Some concerns | Some concerns | Some concerns | No concerns | Very low |
| Amitriptyline 75mg:BTX-A 50U | 0 | Some concerns | Low risk | Some concerns | Some concerns | No concerns | No concerns | Very low |
| Amitriptyline 75mg:BTX-A 86U | 0 | Some concerns | Low risk | Some concerns | Some concerns | No concerns | No concerns | Very low |
| Amitriptyline 75mg:Ibuprofen 400mg | 0 | Some concerns | Low risk | Some concerns | Some concerns | No concerns | No concerns | Very low |
| Amitriptyline 75mg:L-5-Hydroxytryptophan 100mg | 0 | Some concerns | Low risk | Some concerns | Some concerns | Some concerns | No concerns | Very low |
| Amitriptyline 75mg:Lidocaine 25ml | 0 | Some concerns | Low risk | Some concerns | Some concerns | No concerns | No concerns | Very low |
| Amitriptyline 75mg:Mitazapine 30mg | 0 | Some concerns | Low risk | Some concerns | Some concerns | Some concerns | No concerns | Very low |
| Amitriptyline 75mg:Mitazapine 4.5mg | 0 | Some concerns | Low risk | Some concerns | Some concerns | Some concerns | No concerns | Very low |
| Amitriptyline 75mg:Sodium valproate 1g | 0 | Some concerns | Low risk | Some concerns | Major concerns | No concerns | No concerns | Very low |
| Amitriptylinoxide 90mg:BTX-A 100U | 0 | Some concerns | Low risk | Some concerns | Some concerns | Major concerns | No concerns | Very low |
| Amitriptylinoxide 90mg:BTX-A 150U | 0 | No concerns | Low risk | Some concerns | Some concerns | Major concerns | No concerns | Very low |
| Amitriptylinoxide 90mg:BTX-A 20U | 0 | Some concerns | Low risk | Some concerns | Some concerns | Some concerns | No concerns | Very low |
| Amitriptylinoxide 90mg:BTX-A 210U | 0 | No concerns | Low risk | Some concerns | Some concerns | Some concerns | No concerns | Very low |
| Amitriptylinoxide 90mg:BTX-A 420U | 0 | No concerns | Low risk | Some concerns | Some concerns | No concerns | No concerns | Low |
| Amitriptylinoxide 90mg:BTX-A 500U | 0 | No concerns | Low risk | Some concerns | Some concerns | No concerns | No concerns | Low |
| Amitriptylinoxide 90mg:BTX-A 50U | 0 | No concerns | Low risk | Some concerns | Some concerns | Major concerns | No concerns | Very low |
| Amitriptylinoxide 90mg:BTX-A 86U | 0 | No concerns | Low risk | Some concerns | Some concerns | Major concerns | No concerns | Very low |
| Amitriptylinoxide 90mg:Citalopram 20mg | 0 | Some concerns | Low risk | Some concerns | Some concerns | Some concerns | No concerns | Very low |
| Amitriptylinoxide 90mg:Ibuprofen 400mg | 0 | No concerns | Low risk | Some concerns | Some concerns | Some concerns | No concerns | Very low |
| Amitriptylinoxide 90mg:L-5-Hydroxytryptophan 100mg | 0 | Some concerns | Low risk | Some concerns | Some concerns | Some concerns | No concerns | Very low |
| Amitriptylinoxide 90mg:Lidocaine 25ml | 0 | Some concerns | Low risk | Some concerns | Some concerns | Some concerns | No concerns | Very low |
| Amitriptylinoxide 90mg:Mitazapine 30mg | 0 | No concerns | Low risk | Some concerns | Some concerns | Some concerns | No concerns | Very low |
| Amitriptylinoxide 90mg:Mitazapine 4.5mg | 0 | No concerns | Low risk | Some concerns | Some concerns | Some concerns | No concerns | Very low |
| Amitriptylinoxide 90mg:Sodium valproate 1g | 0 | Some concerns | Low risk | Some concerns | Major concerns | No concerns | No concerns | Very low |
| BTX-A 100U:BTX-A 20U | 0 | Some concerns | Low risk | Some concerns | Some concerns | Some concerns | No concerns | Very low |
| BTX-A 100U:BTX-A 210U | 0 | Some concerns | Low risk | Some concerns | Some concerns | No concerns | No concerns | Very low |
| BTX-A 100U:BTX-A 420U | 0 | Some concerns | Low risk | Some concerns | Some concerns | No concerns | No concerns | Very low |
| BTX-A 100U:BTX-A 500U | 0 | Some concerns | Low risk | Some concerns | Some concerns | No concerns | No concerns | Very low |
| BTX-A 100U:Citalopram 20mg | 0 | Some concerns | Low risk | Some concerns | Some concerns | Major concerns | No concerns | Very low |
| BTX-A 100U:Ibuprofen 400mg | 0 | Some concerns | Low risk | Some concerns | Some concerns | Major concerns | No concerns | Very low |
| BTX-A 100U:L-5-Hydroxytryptophan 100mg | 0 | Some concerns | Low risk | Some concerns | Some concerns | Some concerns | No concerns | Very low |
| BTX-A 100U:Lidocaine 25ml | 0 | Some concerns | Low risk | Some concerns | Some concerns | Some concerns | No concerns | Very low |
| BTX-A 100U:Mitazapine 30mg | 0 | Some concerns | Low risk | Some concerns | Some concerns | Some concerns | No concerns | Very low |
| BTX-A 100U:Mitazapine 4.5mg | 0 | Some concerns | Low risk | Some concerns | Some concerns | Some concerns | No concerns | Very low |
| BTX-A 100U:Sodium valproate 1g | 0 | Some concerns | Low risk | Some concerns | Some concerns | Some concerns | No concerns | Very low |
| BTX-A 150U:BTX-A 20U | 0 | Some concerns | Low risk | Some concerns | Some concerns | Some concerns | No concerns | Very low |
| BTX-A 150U:BTX-A 210U | 0 | No concerns | Low risk | Some concerns | Some concerns | No concerns | No concerns | Low |
| BTX-A 150U:BTX-A 420U | 0 | No concerns | Low risk | Some concerns | Some concerns | No concerns | No concerns | Low |
| BTX-A 150U:BTX-A 500U | 0 | No concerns | Low risk | Some concerns | Some concerns | No concerns | No concerns | Low |
| BTX-A 150U:Citalopram 20mg | 0 | Some concerns | Low risk | Some concerns | Some concerns | Some concerns | No concerns | Very low |
| BTX-A 150U:Ibuprofen 400mg | 0 | No concerns | Low risk | Some concerns | Some concerns | Major concerns | No concerns | Very low |
| BTX-A 150U:L-5-Hydroxytryptophan 100mg | 0 | Some concerns | Low risk | Some concerns | Some concerns | Some concerns | No concerns | Very low |
| BTX-A 150U:Lidocaine 25ml | 0 | Some concerns | Low risk | Some concerns | Some concerns | Some concerns | No concerns | Very low |
| BTX-A 150U:Mitazapine 30mg | 0 | No concerns | Low risk | Some concerns | Some concerns | Some concerns | No concerns | Very low |
| BTX-A 150U:Mitazapine 4.5mg | 0 | No concerns | Low risk | Some concerns | Some concerns | Some concerns | No concerns | Very low |
| BTX-A 150U:Sodium valproate 1g | 0 | Some concerns | Low risk | Some concerns | Some concerns | Some concerns | No concerns | Very low |
| BTX-A 20U:BTX-A 210U | 0 | Some concerns | Low risk | Some concerns | Some concerns | Some concerns | No concerns | Very low |
| BTX-A 20U:BTX-A 420U | 0 | Some concerns | Low risk | Some concerns | Some concerns | Some concerns | No concerns | Very low |
| BTX-A 20U:BTX-A 500U | 0 | Some concerns | Low risk | Some concerns | Some concerns | Some concerns | No concerns | Very low |
| BTX-A 20U:BTX-A 50U | 0 | Some concerns | Low risk | Some concerns | Some concerns | Some concerns | No concerns | Very low |
| BTX-A 20U:BTX-A 86U | 0 | Some concerns | Low risk | Some concerns | Some concerns | Some concerns | No concerns | Very low |
| BTX-A 20U:Citalopram 20mg | 0 | Some concerns | Low risk | Some concerns | Some concerns | No concerns | No concerns | Very low |
| BTX-A 20U:Ibuprofen 400mg | 0 | Some concerns | Low risk | Some concerns | Some concerns | Some concerns | No concerns | Very low |
| BTX-A 20U:L-5-Hydroxytryptophan 100mg | 0 | Major concerns | Low risk | Some concerns | Some concerns | Some concerns | No concerns | Very low |
| BTX-A 20U:Lidocaine 25ml | 0 | Some concerns | Low risk | Some concerns | Some concerns | Some concerns | No concerns | Very low |
| BTX-A 20U:Mitazapine 30mg | 0 | Some concerns | Low risk | Some concerns | Some concerns | Some concerns | No concerns | Very low |
| BTX-A 20U:Mitazapine 4.5mg | 0 | Some concerns | Low risk | Some concerns | Major concerns | No concerns | No concerns | Very low |
| BTX-A 20U:Sodium valproate 1g | 0 | Some concerns | Low risk | Some concerns | Major concerns | No concerns | No concerns | Very low |
| BTX-A 210U:BTX-A 500U | 0 | No concerns | Low risk | Some concerns | Some concerns | Some concerns | No concerns | Very low |
| BTX-A 210U:BTX-A 50U | 0 | No concerns | Low risk | Some concerns | Some concerns | No concerns | No concerns | Very low |
| BTX-A 210U:BTX-A 86U | 0 | No concerns | Low risk | Some concerns | Some concerns | No concerns | No concerns | Very low |
| BTX-A 210U:Citalopram 20mg | 0 | Some concerns | Low risk | Some concerns | Some concerns | No concerns | No concerns | Very low |
| BTX-A 210U:Ibuprofen 400mg | 0 | No concerns | Low risk | Some concerns | Some concerns | Some concerns | No concerns | Very low |
| BTX-A 210U:L-5-Hydroxytryptophan 100mg | 0 | Some concerns | Low risk | Some concerns | Some concerns | Some concerns | No concerns | Very low |
| BTX-A 210U:Lidocaine 25ml | 0 | Some concerns | Low risk | Some concerns | Some concerns | No concerns | No concerns | Very low |
| BTX-A 210U:Mitazapine 30mg | 0 | No concerns | Low risk | Some concerns | Major concerns | No concerns | No concerns | Very low |
| BTX-A 210U:Mitazapine 4.5mg | 0 | No concerns | Low risk | Some concerns | Some concerns | Some concerns | No concerns | Very low |
| BTX-A 210U:Sodium valproate 1g | 0 | No concerns | Low risk | Some concerns | Major concerns | No concerns | No concerns | Very low |
| BTX-A 420U:BTX-A 500U | 0 | No concerns | Low risk | Some concerns | Some concerns | Some concerns | No concerns | Very low |
| BTX-A 420U:BTX-A 50U | 0 | No concerns | Low risk | Some concerns | Some concerns | No concerns | No concerns | Low |
| BTX-A 420U:BTX-A 86U | 0 | No concerns | Low risk | Some concerns | Some concerns | No concerns | No concerns | Low |
| BTX-A 420U:Citalopram 20mg | 0 | Some concerns | Low risk | Some concerns | Some concerns | No concerns | No concerns | Very low |
| BTX-A 420U:Ibuprofen 400mg | 0 | No concerns | Low risk | Some concerns | Some concerns | Some concerns | No concerns | Very low |
| BTX-A 420U:L-5-Hydroxytryptophan 100mg | 0 | Some concerns | Low risk | Some concerns | Some concerns | Some concerns | No concerns | Very low |
| BTX-A 420U:Lidocaine 25ml | 0 | Some concerns | Low risk | Some concerns | Some concerns | No concerns | No concerns | Very low |
| BTX-A 420U:Mitazapine 30mg | 0 | No concerns | Low risk | Some concerns | Major concerns | No concerns | No concerns | Very low |
| BTX-A 420U:Mitazapine 4.5mg | 0 | No concerns | Low risk | Some concerns | Some concerns | Some concerns | No concerns | Very low |
| BTX-A 420U:Sodium valproate 1g | 0 | No concerns | Low risk | Some concerns | Major concerns | No concerns | No concerns | Very low |
| BTX-A 500U:BTX-A 50U | 0 | No concerns | Low risk | Some concerns | Some concerns | No concerns | No concerns | Low |
| BTX-A 500U:BTX-A 86U | 0 | No concerns | Low risk | Some concerns | Some concerns | No concerns | No concerns | Low |
| BTX-A 500U:Citalopram 20mg | 0 | Some concerns | Low risk | Some concerns | Some concerns | Some concerns | No concerns | Very low |
| BTX-A 500U:Ibuprofen 400mg | 0 | No concerns | Low risk | Some concerns | Some concerns | Some concerns | No concerns | Very low |
| BTX-A 500U:L-5-Hydroxytryptophan 100mg | 0 | Some concerns | Low risk | Some concerns | Some concerns | Some concerns | No concerns | Very low |
| BTX-A 500U:Lidocaine 25ml | 0 | Some concerns | Low risk | Some concerns | Some concerns | Some concerns | No concerns | Very low |
| BTX-A 500U:Mitazapine 30mg | 0 | No concerns | Low risk | Some concerns | Major concerns | No concerns | No concerns | Very low |
| BTX-A 500U:Mitazapine 4.5mg | 0 | No concerns | Low risk | Some concerns | Some concerns | Some concerns | No concerns | Very low |
| BTX-A 500U:Sodium valproate 1g | 0 | Some concerns | Low risk | Some concerns | Some concerns | Some concerns | No concerns | Very low |
| BTX-A 50U:Citalopram 20mg | 0 | Some concerns | Low risk | Some concerns | Some concerns | Major concerns | No concerns | Very low |
| BTX-A 50U:Ibuprofen 400mg | 0 | No concerns | Low risk | Some concerns | Some concerns | Major concerns | No concerns | Very low |
| BTX-A 50U:L-5-Hydroxytryptophan 100mg | 0 | Some concerns | Low risk | Some concerns | Some concerns | Some concerns | No concerns | Very low |
| BTX-A 50U:Lidocaine 25ml | 0 | No concerns | Low risk | Some concerns | Some concerns | Some concerns | No concerns | Very low |
| BTX-A 50U:Mitazapine 30mg | 0 | No concerns | Low risk | Some concerns | Some concerns | Some concerns | No concerns | Very low |
| BTX-A 50U:Mitazapine 4.5mg | 0 | No concerns | Low risk | Some concerns | Some concerns | Some concerns | No concerns | Very low |
| BTX-A 50U:Sodium valproate 1g | 0 | Some concerns | Low risk | Some concerns | Some concerns | Some concerns | No concerns | Very low |
| BTX-A 86U:Citalopram 20mg | 0 | Some concerns | Low risk | Some concerns | Some concerns | Some concerns | No concerns | Very low |
| BTX-A 86U:Ibuprofen 400mg | 0 | No concerns | Low risk | Some concerns | Some concerns | Some concerns | No concerns | Very low |
| BTX-A 86U:L-5-Hydroxytryptophan 100mg | 0 | Some concerns | Low risk | Some concerns | Some concerns | Some concerns | No concerns | Very low |
| BTX-A 86U:Lidocaine 25ml | 0 | Some concerns | Low risk | Some concerns | Some concerns | Some concerns | No concerns | Very low |
| BTX-A 86U:Mitazapine 30mg | 0 | No concerns | Low risk | Some concerns | Some concerns | Some concerns | No concerns | Very low |
| BTX-A 86U:Mitazapine 4.5mg | 0 | No concerns | Low risk | Some concerns | Some concerns | Some concerns | No concerns | Very low |
| BTX-A 86U:Sodium valproate 1g | 0 | No concerns | Low risk | Some concerns | Some concerns | Some concerns | No concerns | Very low |
| Citalopram 20mg:Ibuprofen 400mg | 0 | Some concerns | Low risk | Some concerns | Some concerns | Some concerns | No concerns | Very low |
| Citalopram 20mg:L-5-Hydroxytryptophan 100mg | 0 | Major concerns | Low risk | Some concerns | Some concerns | Some concerns | No concerns | Very low |
| Citalopram 20mg:Lidocaine 25ml | 0 | Some concerns | Low risk | Some concerns | Some concerns | Some concerns | No concerns | Very low |
| Citalopram 20mg:Mitazapine 30mg | 0 | Some concerns | Low risk | Some concerns | Some concerns | No concerns | No concerns | Very low |
| Citalopram 20mg:Mitazapine 4.5mg | 0 | Some concerns | Low risk | Some concerns | Some concerns | Some concerns | No concerns | Very low |
| Citalopram 20mg:Sodium valproate 1g | 0 | Some concerns | Low risk | Some concerns | Some concerns | Some concerns | No concerns | Very low |
| Ibuprofen 400mg:L-5-Hydroxytryptophan 100mg | 0 | Some concerns | Low risk | Some concerns | Major concerns | No concerns | No concerns | Very low |
| Ibuprofen 400mg:Lidocaine 25ml | 0 | Some concerns | Low risk | Some concerns | Some concerns | Some concerns | No concerns | Very low |
| Ibuprofen 400mg:Mitazapine 30mg | 0 | No concerns | Low risk | Some concerns | Some concerns | Some concerns | No concerns | Very low |
| Ibuprofen 400mg:Sodium valproate 1g | 0 | Some concerns | Low risk | Some concerns | Major concerns | No concerns | No concerns | Very low |
| L-5-Hydroxytryptophan 100mg:Lidocaine 25ml | 0 | Major concerns | Low risk | Some concerns | Some concerns | Some concerns | No concerns | Very low |
| L-5-Hydroxytryptophan 100mg:Mitazapine 30mg | 0 | Some concerns | Low risk | Some concerns | Some concerns | Some concerns | No concerns | Very low |
| L-5-Hydroxytryptophan 100mg:Mitazapine 4.5mg | 0 | Some concerns | Low risk | Some concerns | Some concerns | Some concerns | No concerns | Very low |
| L-5-Hydroxytryptophan 100mg:Sodium valproate 1g | 0 | Major concerns | Low risk | Some concerns | Major concerns | No concerns | No concerns | Very low |
| Lidocaine 25ml:Mitazapine 30mg | 0 | Some concerns | Low risk | Some concerns | Some concerns | No concerns | No concerns | Very low |
| Lidocaine 25ml:Mitazapine 4.5mg | 0 | Some concerns | Low risk | Some concerns | Some concerns | Some concerns | No concerns | Very low |
| Lidocaine 25ml:Sodium valproate 1g | 0 | Some concerns | Low risk | Some concerns | Some concerns | Some concerns | No concerns | Very low |
| Mitazapine 30mg:Mitazapine 4.5mg | 0 | No concerns | Low risk | Some concerns | Some concerns | Some concerns | No concerns | Very low |
| Mitazapine 30mg:Sodium valproate 1g | 0 | Some concerns | Low risk | Some concerns | Some concerns | Some concerns | No concerns | Very low |
| Mitazapine 4.5mg:Sodium valproate 1g | 0 | Some concerns | Low risk | Some concerns | Major concerns | No concerns | No concerns | Very low |

**Footnote:** BTX-A, botulinum toxin type-A.

Table S7. The relative effects between different interventions for headache days per month at 4 weeks

| **Treatment** | **Control** | **Mean difference** | **95% CrI** | |
| --- | --- | --- | --- | --- |
| **Amitriptyline 100mg** | **Placebo** | **-6.59** | **-11.22** | **-0.64** |
| BTX-A 20U | Placebo | 0.54 | -11.56 | 13.12 |
| BTX-A 50U | Placebo | -2.56 | -11.88 | 7.07 |
| BTX-A 100U | Placebo | -3.21 | -7.27 | 0.99 |
| Sodium valproate 1g | Placebo | -5.84 | -19.04 | 7.59 |
| Tizanidine 6mg | Placebo | -2.34 | -7.28 | 2.67 |
| Tizanidine 12mg | Placebo | 0.75 | -4.24 | 5.48 |
| BTX-A 20U | Amitriptyline 100mg | 7.13 | -6.06 | 20.05 |
| BTX-A 50U | Amitriptyline 100mg | 4.03 | -7.08 | 14.29 |
| BTX-A 100U | Amitriptyline 100mg | 3.38 | -4.11 | 9.89 |
| Sodium valproate 1g | Amitriptyline 100mg | 0.75 | -13.25 | 14.82 |
| Tizanidine 6mg | Amitriptyline 100mg | 4.25 | -3.44 | 11.21 |
| Tizanidine 12mg | Amitriptyline 100mg | 7.35 | -1.03 | 13.82 |
| BTX-A 50U | BTX-A 20U | -3.10 | -18.57 | 11.81 |
| BTX-A 100U | BTX-A 20U | -3.74 | -16.89 | 8.83 |
| Sodium valproate 1g | BTX-A 20U | -6.37 | -24.25 | 11.57 |
| Tizanidine 6mg | BTX-A 20U | -2.87 | -15.74 | 9.98 |
| Tizanidine 12mg | BTX-A 20U | 0.22 | -12.55 | 12.88 |
| BTX-A 100U | BTX-A 50U | -0.65 | -11.00 | 9.61 |
| Sodium valproate 1g | BTX-A 50U | -3.28 | -19.73 | 13.07 |
| Tizanidine 6mg | BTX-A 50U | 0.22 | -10.31 | 11.04 |
| Tizanidine 12mg | BTX-A 50U | 3.31 | -7.38 | 13.67 |
| Sodium valproate 1g | BTX-A 100U | -2.63 | -16.30 | 11.30 |
| Tizanidine 6mg | BTX-A 100U | 0.87 | -5.19 | 7.49 |
| Tizanidine 12mg | BTX-A 100U | 3.96 | -2.25 | 10.06 |
| Tizanidine 6mg | Sodium valproate 1g | 3.50 | -10.38 | 17.49 |
| Tizanidine 12mg | Sodium valproate 1g | 6.59 | -7.48 | 20.74 |
| Tizanidine 12mg | Tizanidine 6mg | 3.09 | -2.73 | 8.15 |

Table S8. The relative effects between different interventions for headache days per month at 8 weeks

| **Treatment** | **Control** | **Mean difference** | **95% CrI** | |
| --- | --- | --- | --- | --- |
| Amitriptyline 75mg | Placebo | -2.87 | -6.94 | 1.76 |
| **Amitriptyline 100mg** | **Placebo** | **-6.14** | **-10.27** | **-0.87** |
| BTX-A 20U | Placebo | -0.54 | -12.64 | 11.41 |
| BTX-A 50U | Placebo | -0.92 | -13.41 | 11.70 |
| BTX-A 86U | Placebo | -0.72 | -13.05 | 11.46 |
| **BTX-A 100U** | **Placebo** | **-3.79** | **-7.16** | **-0.33** |
| BTX-A 150U | Placebo | -0.94 | -13.39 | 11.86 |
| BTX-A 210U | Placebo | -0.48 | -12.96 | 12.01 |
| BTX-A 420U | Placebo | -0.39 | -11.79 | 11.09 |
| Citalopram 20mg | Placebo | 0.03 | -4.05 | 5.56 |
| Flunarizine 5mg | Placebo | -1.70 | -11.82 | 8.49 |
| Flunarizine 10mg | Placebo | -3.41 | -13.18 | 6.51 |
| Ibuprofen 400mg | Placebo | -0.62 | -11.37 | 10.61 |
| L-5-Hydroxytryptophan 100mg | Placebo | 0.39 | -12.97 | 13.74 |
| Mirtazapine 4.5mg | Placebo | -0.03 | -5.72 | 5.97 |
| Mirtazapine 30mg | Placebo | -2.09 | -11.11 | 6.79 |
| Tizanidine 6mg | Placebo | -1.22 | -5.71 | 2.50 |
| Tizanidine 12mg | Placebo | 1.60 | -2.62 | 5.88 |
| Amitriptyline 100mg | Amitriptyline 75mg | -3.26 | -9.46 | 3.06 |
| BTX-A 20U | Amitriptyline 75mg | 2.34 | -11.03 | 15.16 |
| BTX-A 50U | Amitriptyline 75mg | 1.95 | -11.33 | 15.32 |
| BTX-A 86U | Amitriptyline 75mg | 2.16 | -11.31 | 15.16 |
| BTX-A 100U | Amitriptyline 75mg | -0.91 | -6.64 | 4.19 |
| BTX-A 150U | Amitriptyline 75mg | 1.94 | -11.02 | 15.55 |
| BTX-A 210U | Amitriptyline 75mg | 2.40 | -10.61 | 15.20 |
| BTX-A 420U | Amitriptyline 75mg | 2.49 | -10.37 | 14.16 |
| Citalopram 20mg | Amitriptyline 75mg | 2.91 | -1.38 | 7.77 |
| Flunarizine 5mg | Amitriptyline 75mg | 1.17 | -9.61 | 12.38 |
| Flunarizine 10mg | Amitriptyline 75mg | -0.53 | -11.13 | 9.95 |
| Ibuprofen 400mg | Amitriptyline 75mg | 2.26 | -9.56 | 13.80 |
| L-5-Hydroxytryptophan 100mg | Amitriptyline 75mg | 3.27 | -10.82 | 17.30 |
| Mirtazapine 4.5mg | Amitriptyline 75mg | 2.84 | -4.70 | 9.92 |
| Mirtazapine 30mg | Amitriptyline 75mg | 0.79 | -9.48 | 10.35 |
| Tizanidine 6mg | Amitriptyline 75mg | 1.66 | -5.17 | 7.10 |
| Tizanidine 12mg | Amitriptyline 75mg | 4.47 | -2.09 | 10.22 |
| BTX-A 20U | Amitriptyline 100mg | 5.60 | -8.02 | 18.54 |
| BTX-A 50U | Amitriptyline 100mg | 5.22 | -9.04 | 18.33 |
| BTX-A 86U | Amitriptyline 100mg | 5.42 | -8.16 | 18.39 |
| BTX-A 100U | Amitriptyline 100mg | 2.35 | -2.79 | 7.52 |
| BTX-A 150U | Amitriptyline 100mg | 5.20 | -7.98 | 18.59 |
| BTX-A 210U | Amitriptyline 100mg | 5.66 | -7.30 | 18.49 |
| BTX-A 420U | Amitriptyline 100mg | 5.75 | -6.19 | 17.65 |
| Citalopram 20mg | Amitriptyline 100mg | 6.17 | -0.01 | 12.29 |
| Flunarizine 5mg | Amitriptyline 100mg | 4.43 | -6.75 | 15.47 |
| Flunarizine 10mg | Amitriptyline 100mg | 2.73 | -8.36 | 13.63 |
| Ibuprofen 400mg | Amitriptyline 100mg | 5.52 | -6.31 | 17.81 |
| L-5-Hydroxytryptophan 100mg | Amitriptyline 100mg | 6.53 | -7.07 | 20.33 |
| Mirtazapine 4.5mg | Amitriptyline 100mg | 6.11 | -1.77 | 13.14 |
| Mirtazapine 30mg | Amitriptyline 100mg | 4.05 | -6.28 | 14.05 |
| Tizanidine 6mg | Amitriptyline 100mg | 4.92 | -2.25 | 10.72 |
| **Tizanidine 12mg** | **Amitriptyline 100mg** | **7.74** | **1.43** | **13.40** |
| BTX-A 50U | BTX-A 20U | -0.38 | -17.67 | 17.04 |
| BTX-A 86U | BTX-A 20U | -0.18 | -17.41 | 16.74 |
| BTX-A 100U | BTX-A 20U | -3.25 | -15.70 | 9.79 |
| BTX-A 150U | BTX-A 20U | -0.40 | -17.93 | 17.35 |
| BTX-A 210U | BTX-A 20U | 0.06 | -17.42 | 17.54 |
| BTX-A 420U | BTX-A 20U | 0.15 | -16.31 | 16.35 |
| Citalopram 20mg | BTX-A 20U | 0.57 | -12.26 | 14.07 |
| Flunarizine 5mg | BTX-A 20U | -1.17 | -16.70 | 14.21 |
| Flunarizine 10mg | BTX-A 20U | -2.87 | -18.60 | 12.63 |
| Ibuprofen 400mg | BTX-A 20U | -0.08 | -16.03 | 17.00 |
| L-5-Hydroxytryptophan 100mg | BTX-A 20U | 0.93 | -17.09 | 18.85 |
| Mirtazapine 4.5mg | BTX-A 20U | 0.51 | -12.86 | 13.51 |
| Mirtazapine 30mg | BTX-A 20U | -1.55 | -15.74 | 12.70 |
| Tizanidine 6mg | BTX-A 20U | -0.68 | -13.15 | 12.18 |
| Tizanidine 12mg | BTX-A 20U | 2.14 | -10.33 | 15.93 |
| BTX-A 86U | BTX-A 50U | 0.20 | -15.29 | 15.71 |
| BTX-A 100U | BTX-A 50U | -2.87 | -15.67 | 10.51 |
| BTX-A 150U | BTX-A 50U | -0.02 | -15.58 | 16.18 |
| BTX-A 210U | BTX-A 50U | 0.44 | -17.34 | 18.13 |
| BTX-A 420U | BTX-A 50U | 0.53 | -16.64 | 17.78 |
| Citalopram 20mg | BTX-A 50U | 0.95 | -12.12 | 15.33 |
| Flunarizine 5mg | BTX-A 50U | -0.78 | -17.03 | 14.59 |
| Flunarizine 10mg | BTX-A 50U | -2.48 | -17.90 | 13.45 |
| Ibuprofen 400mg | BTX-A 50U | 0.31 | -16.32 | 16.90 |
| L-5-Hydroxytryptophan 100mg | BTX-A 50U | 1.32 | -17.88 | 20.15 |
| Mirtazapine 4.5mg | BTX-A 50U | 0.89 | -12.94 | 14.80 |
| Mirtazapine 30mg | BTX-A 50U | -1.16 | -16.25 | 13.89 |
| Tizanidine 6mg | BTX-A 50U | -0.30 | -13.41 | 12.17 |
| Tizanidine 12mg | BTX-A 50U | 2.52 | -10.69 | 16.30 |
| BTX-A 100U | BTX-A 86U | -3.07 | -15.71 | 10.17 |
| BTX-A 150U | BTX-A 86U | -0.22 | -15.81 | 15.48 |
| BTX-A 210U | BTX-A 86U | 0.24 | -17.83 | 17.33 |
| BTX-A 420U | BTX-A 86U | 0.33 | -16.90 | 17.02 |
| Citalopram 20mg | BTX-A 86U | 0.75 | -11.92 | 14.37 |
| Flunarizine 5mg | BTX-A 86U | -0.98 | -17.04 | 15.19 |
| Flunarizine 10mg | BTX-A 86U | -2.69 | -18.30 | 12.81 |
| Ibuprofen 400mg | BTX-A 86U | 0.10 | -15.97 | 16.46 |
| L-5-Hydroxytryptophan 100mg | BTX-A 86U | 1.11 | -17.59 | 19.21 |
| Mirtazapine 4.5mg | BTX-A 86U | 0.69 | -12.62 | 14.11 |
| Mirtazapine 30mg | BTX-A 86U | -1.37 | -16.35 | 13.52 |
| Tizanidine 6mg | BTX-A 86U | -0.50 | -13.12 | 12.55 |
| Tizanidine 12mg | BTX-A 86U | 2.32 | -10.61 | 15.96 |
| BTX-A 150U | BTX-A 100U | 2.85 | -9.86 | 16.05 |
| BTX-A 210U | BTX-A 100U | 3.31 | -9.56 | 16.15 |
| BTX-A 420U | BTX-A 100U | 3.40 | -8.65 | 15.01 |
| Citalopram 20mg | BTX-A 100U | 3.82 | -1.60 | 9.50 |
| Flunarizine 5mg | BTX-A 100U | 2.08 | -8.49 | 12.95 |
| Flunarizine 10mg | BTX-A 100U | 0.38 | -10.00 | 10.89 |
| Ibuprofen 400mg | BTX-A 100U | 3.17 | -8.25 | 15.02 |
| L-5-Hydroxytryptophan 100mg | BTX-A 100U | 4.18 | -9.34 | 17.84 |
| Mirtazapine 4.5mg | BTX-A 100U | 3.75 | -3.58 | 10.29 |
| Mirtazapine 30mg | BTX-A 100U | 1.70 | -8.80 | 11.35 |
| Tizanidine 6mg | BTX-A 100U | 2.57 | -3.46 | 8.09 |
| Tizanidine 12mg | BTX-A 100U | 5.39 | -0.16 | 10.55 |
| BTX-A 210U | BTX-A 150U | 0.46 | -18.02 | 18.58 |
| BTX-A 420U | BTX-A 150U | 0.55 | -17.01 | 17.61 |
| Citalopram 20mg | BTX-A 150U | 0.97 | -12.52 | 14.06 |
| Flunarizine 5mg | BTX-A 150U | -0.77 | -16.86 | 15.58 |
| Flunarizine 10mg | BTX-A 150U | -2.47 | -19.01 | 13.15 |
| Ibuprofen 400mg | BTX-A 150U | 0.32 | -16.18 | 16.83 |
| L-5-Hydroxytryptophan 100mg | BTX-A 150U | 1.33 | -17.15 | 20.32 |
| Mirtazapine 4.5mg | BTX-A 150U | 0.90 | -13.03 | 14.52 |
| Mirtazapine 30mg | BTX-A 150U | -1.15 | -16.39 | 13.77 |
| Tizanidine 6mg | BTX-A 150U | -0.28 | -13.18 | 12.81 |
| Tizanidine 12mg | BTX-A 150U | 2.54 | -10.69 | 15.50 |
| BTX-A 420U | BTX-A 210U | 0.09 | -12.83 | 13.10 |
| Citalopram 20mg | BTX-A 210U | 0.51 | -12.75 | 13.90 |
| Flunarizine 5mg | BTX-A 210U | -1.23 | -17.30 | 15.10 |
| Flunarizine 10mg | BTX-A 210U | -2.93 | -18.30 | 12.75 |
| Ibuprofen 400mg | BTX-A 210U | -0.14 | -16.80 | 16.85 |
| L-5-Hydroxytryptophan 100mg | BTX-A 210U | 0.87 | -17.25 | 19.13 |
| Mirtazapine 4.5mg | BTX-A 210U | 0.44 | -12.96 | 14.41 |
| Mirtazapine 30mg | BTX-A 210U | -1.61 | -16.87 | 14.16 |
| Tizanidine 6mg | BTX-A 210U | -0.74 | -13.83 | 12.42 |
| Tizanidine 12mg | BTX-A 210U | 2.08 | -11.25 | 14.94 |
| Citalopram 20mg | BTX-A 420U | 0.42 | -11.57 | 13.19 |
| Flunarizine 5mg | BTX-A 420U | -1.32 | -16.50 | 13.88 |
| Flunarizine 10mg | BTX-A 420U | -3.02 | -17.68 | 12.13 |
| Ibuprofen 400mg | BTX-A 420U | -0.23 | -15.76 | 16.28 |
| L-5-Hydroxytryptophan 100mg | BTX-A 420U | 0.78 | -16.63 | 18.39 |
| Mirtazapine 4.5mg | BTX-A 420U | 0.36 | -12.25 | 13.20 |
| Mirtazapine 30mg | BTX-A 420U | -1.70 | -16.21 | 13.29 |
| Tizanidine 6mg | BTX-A 420U | -0.83 | -12.81 | 11.33 |
| Tizanidine 12mg | BTX-A 420U | 1.99 | -9.79 | 14.67 |
| Flunarizine 5mg | Citalopram 20mg | -1.74 | -12.64 | 9.38 |
| Flunarizine 10mg | Citalopram 20mg | -3.44 | -14.54 | 6.97 |
| Ibuprofen 400mg | Citalopram 20mg | -0.65 | -12.21 | 11.34 |
| L-5-Hydroxytryptophan 100mg | Citalopram 20mg | 0.36 | -13.52 | 14.50 |
| Mirtazapine 4.5mg | Citalopram 20mg | -0.06 | -8.49 | 7.28 |
| Mirtazapine 30mg | Citalopram 20mg | -2.12 | -12.97 | 7.50 |
| Tizanidine 6mg | Citalopram 20mg | -1.25 | -8.63 | 4.23 |
| Tizanidine 12mg | Citalopram 20mg | 1.57 | -4.88 | 7.20 |
| Flunarizine 10mg | Flunarizine 5mg | -1.70 | -13.24 | 9.66 |
| Ibuprofen 400mg | Flunarizine 5mg | 1.09 | -14.23 | 16.31 |
| L-5-Hydroxytryptophan 100mg | Flunarizine 5mg | 2.10 | -14.66 | 18.39 |
| Mirtazapine 4.5mg | Flunarizine 5mg | 1.67 | -9.80 | 13.10 |
| Mirtazapine 30mg | Flunarizine 5mg | -0.38 | -13.49 | 13.06 |
| Tizanidine 6mg | Flunarizine 5mg | 0.49 | -10.25 | 11.20 |
| Tizanidine 12mg | Flunarizine 5mg | 3.30 | -7.47 | 13.97 |
| Ibuprofen 400mg | Flunarizine 10mg | 2.79 | -12.26 | 17.95 |
| L-5-Hydroxytryptophan 100mg | Flunarizine 10mg | 3.80 | -12.98 | 20.48 |
| Mirtazapine 4.5mg | Flunarizine 10mg | 3.37 | -8.24 | 15.04 |
| Mirtazapine 30mg | Flunarizine 10mg | 1.32 | -11.94 | 14.35 |
| Tizanidine 6mg | Flunarizine 10mg | 2.19 | -8.55 | 12.38 |
| Tizanidine 12mg | Flunarizine 10mg | 5.00 | -5.84 | 15.64 |
| L-5-Hydroxytryptophan 100mg | Ibuprofen 400mg | 1.01 | -17.20 | 18.47 |
| Mirtazapine 4.5mg | Ibuprofen 400mg | 0.58 | -11.37 | 12.25 |
| Mirtazapine 30mg | Ibuprofen 400mg | -1.47 | -15.50 | 12.16 |
| Tizanidine 6mg | Ibuprofen 400mg | -0.60 | -12.58 | 10.76 |
| Tizanidine 12mg | Ibuprofen 400mg | 2.21 | -9.54 | 13.68 |
| Mirtazapine 4.5mg | L-5-Hydroxytryptophan 100mg | -0.43 | -14.49 | 14.12 |
| Mirtazapine 30mg | L-5-Hydroxytryptophan 100mg | -2.48 | -18.73 | 13.84 |
| Tizanidine 6mg | L-5-Hydroxytryptophan 100mg | -1.61 | -15.38 | 12.07 |
| Tizanidine 12mg | L-5-Hydroxytryptophan 100mg | 1.20 | -12.88 | 14.99 |
| Mirtazapine 30mg | Mirtazapine 4.5mg | -2.05 | -12.41 | 8.30 |
| Tizanidine 6mg | Mirtazapine 4.5mg | -1.18 | -8.11 | 5.41 |
| Tizanidine 12mg | Mirtazapine 4.5mg | 1.63 | -5.62 | 8.83 |
| Tizanidine 6mg | Mirtazapine 30mg | 0.87 | -8.49 | 10.20 |
| Tizanidine 12mg | Mirtazapine 30mg | 3.68 | -5.80 | 13.74 |
| Tizanidine 12mg | Tizanidine 6mg | 2.82 | -1.63 | 7.36 |

**Footnote:** BTX-A, botulinum toxin type-A; CrI, credible interval.

Table S9. The relative effects between different interventions for headache days per month at 12 weeks

| **Treatment** | **Control** | **Mean difference** | **95% CrI** | |
| --- | --- | --- | --- | --- |
| Amitriptyline 75mg | Placebo | -0.47 | -15.58 | 14.55 |
| Amitriptyline 100mg | Placebo | -5.62 | -13.19 | 2.50 |
| Amitriptylinoxide 90mg | Placebo | -0.56 | -15.82 | 14.41 |
| BTX-A 50U | Placebo | -3.20 | -13.45 | 7.16 |
| BTX-A 100U | Placebo | -3.56 | -9.40 | 2.78 |
| BTX-A 210U | Placebo | -0.74 | -13.98 | 12.63 |
| BTX-A 420U | Placebo | -0.23 | -12.61 | 11.46 |
| BTX-A 500U | Placebo | -0.42 | -11.41 | 9.96 |
| L-5-Hydroxytryptophan 100mg | Placebo | -2.54 | -17.28 | 11.81 |
| Lidocaine 25ml | Placebo | -7.75 | -18.61 | 3.33 |
| Sodium valproate 1g | Placebo | -6.53 | -20.50 | 7.29 |
| Amitriptyline 100mg | Amitriptyline 75mg | -5.15 | -21.79 | 12.02 |
| Amitriptylinoxide 90mg | Amitriptyline 75mg | -0.09 | -17.71 | 17.77 |
| BTX-A 50U | Amitriptyline 75mg | -2.73 | -20.08 | 15.37 |
| BTX-A 100U | Amitriptyline 75mg | -3.09 | -18.70 | 12.94 |
| BTX-A 210U | Amitriptyline 75mg | -0.28 | -20.54 | 19.26 |
| BTX-A 420U | Amitriptyline 75mg | 0.24 | -18.67 | 18.93 |
| BTX-A 500U | Amitriptyline 75mg | 0.05 | -18.23 | 18.20 |
| L-5-Hydroxytryptophan 100mg | Amitriptyline 75mg | -2.08 | -22.35 | 18.36 |
| Lidocaine 25ml | Amitriptyline 75mg | -7.28 | -25.51 | 12.00 |
| Sodium valproate 1g | Amitriptyline 75mg | -6.06 | -27.11 | 13.80 |
| Amitriptylinoxide 90mg | Amitriptyline 100mg | 5.06 | -12.30 | 21.36 |
| BTX-A 50U | Amitriptyline 100mg | 2.42 | -10.54 | 14.99 |
| BTX-A 100U | Amitriptyline 100mg | 2.06 | -8.28 | 11.54 |
| BTX-A 210U | Amitriptyline 100mg | 4.88 | -10.44 | 20.09 |
| BTX-A 420U | Amitriptyline 100mg | 5.39 | -8.98 | 19.19 |
| BTX-A 500U | Amitriptyline 100mg | 5.20 | -9.06 | 18.06 |
| L-5-Hydroxytryptophan 100mg | Amitriptyline 100mg | 3.08 | -13.56 | 18.81 |
| Lidocaine 25ml | Amitriptyline 100mg | -2.13 | -15.57 | 11.35 |
| Sodium valproate 1g | Amitriptyline 100mg | -0.91 | -16.49 | 14.66 |
| BTX-A 50U | Amitriptylinoxide 90mg | -2.65 | -20.71 | 16.11 |
| BTX-A 100U | Amitriptylinoxide 90mg | -3.00 | -18.90 | 12.82 |
| BTX-A 210U | Amitriptylinoxide 90mg | -0.19 | -20.06 | 20.02 |
| BTX-A 420U | Amitriptylinoxide 90mg | 0.33 | -19.36 | 19.81 |
| BTX-A 500U | Amitriptylinoxide 90mg | 0.14 | -18.67 | 18.47 |
| L-5-Hydroxytryptophan 100mg | Amitriptylinoxide 90mg | -1.99 | -23.19 | 18.54 |
| Lidocaine 25ml | Amitriptylinoxide 90mg | -7.20 | -25.37 | 11.62 |
| Sodium valproate 1g | Amitriptylinoxide 90mg | -5.97 | -26.28 | 14.55 |
| BTX-A 100U | BTX-A 50U | -0.36 | -12.10 | 12.21 |
| BTX-A 210U | BTX-A 50U | 2.46 | -14.12 | 19.20 |
| BTX-A 420U | BTX-A 50U | 2.97 | -12.78 | 18.98 |
| BTX-A 500U | BTX-A 50U | 2.78 | -12.41 | 17.33 |
| L-5-Hydroxytryptophan 100mg | BTX-A 50U | 0.66 | -16.96 | 18.74 |
| Lidocaine 25ml | BTX-A 50U | -4.55 | -19.65 | 10.96 |
| Sodium valproate 1g | BTX-A 50U | -3.33 | -20.35 | 14.17 |
| BTX-A 210U | BTX-A 100U | 2.81 | -11.80 | 17.42 |
| BTX-A 420U | BTX-A 100U | 3.33 | -10.11 | 16.67 |
| BTX-A 500U | BTX-A 100U | 3.14 | -8.93 | 14.66 |
| L-5-Hydroxytryptophan 100mg | BTX-A 100U | 1.01 | -14.43 | 16.29 |
| Lidocaine 25ml | BTX-A 100U | -4.19 | -16.74 | 8.69 |
| Sodium valproate 1g | BTX-A 100U | -2.97 | -18.14 | 12.48 |
| BTX-A 420U | BTX-A 210U | 0.52 | -13.39 | 14.43 |
| BTX-A 500U | BTX-A 210U | 0.33 | -16.65 | 17.20 |
| L-5-Hydroxytryptophan 100mg | BTX-A 210U | -1.80 | -20.78 | 17.95 |
| Lidocaine 25ml | BTX-A 210U | -7.01 | -23.77 | 9.87 |
| Sodium valproate 1g | BTX-A 210U | -5.79 | -24.99 | 13.99 |
| BTX-A 500U | BTX-A 420U | -0.19 | -16.20 | 15.59 |
| L-5-Hydroxytryptophan 100mg | BTX-A 420U | -2.32 | -20.71 | 15.95 |
| Lidocaine 25ml | BTX-A 420U | -7.52 | -23.64 | 8.81 |
| Sodium valproate 1g | BTX-A 420U | -6.30 | -24.47 | 12.66 |
| L-5-Hydroxytryptophan 100mg | BTX-A 500U | -2.13 | -20.01 | 15.43 |
| Lidocaine 25ml | BTX-A 500U | -7.33 | -22.66 | 8.25 |
| Sodium valproate 1g | BTX-A 500U | -6.11 | -23.36 | 11.71 |
| Lidocaine 25ml | L-5-Hydroxytryptophan 100mg | -5.21 | -23.20 | 13.04 |
| Sodium valproate 1g | L-5-Hydroxytryptophan 100mg | -3.99 | -24.42 | 16.04 |
| Sodium valproate 1g | Lidocaine 25ml | 1.22 | -15.94 | 18.88 |

**Footnote:** BTX-A, botulinum toxin type-A; CrI, credible interval.

Table S10. The relative effects between different interventions for headache days per month at 24 weeks

| **Treatment** | **Control** | **Mean difference** | **95% CrI** | |
| --- | --- | --- | --- | --- |
| Amitriptyline 100mg | Placebo | -5.12 | -12.28 | 4.71 |
| BTX-A 50U | Placebo | -1.32 | -9.01 | 7.64 |
| BTX-A 50U | Amitriptyline 100mg | 3.80 | -7.51 | 13.83 |

**Footnote:** BTX-A, botulinum toxin type-A; CrI, credible interval.

Table S11. Value of SUCRA for each treatment on outcomes

| **Treatment** | **SUCRA** | | | | | | | | | | | |
| --- | --- | --- | --- | --- | --- | --- | --- | --- | --- | --- | --- | --- |
|  | **Headache days per month** | | | | **Headache intensity** | | | | **Headache duration** | | | **Adverse event rate** |
|  | **4 weeks** | **8 weeks** | **12 weeks** | **24 weeks** | **4 weeks** | **8 weeks** | **12 weeks** | **24 weeks** | **4 weeks** | **8 weeks** | **12 weeks** |  |
| Amitriptyline 100mg | **0.85** | **0.85** | 0.69 | **0.87** | 0.52 | 0.59 | 0.58 | **0.62** | NA | NA | NA | 0.18 |
| Amitriptyline 25mg | NA | NA | NA | NA | NA | NA | NA | NA | NA | NA | NA | 0.44 |
| Amitriptyline 75mg | NA | 0.65 | 0.40 | NA | NA | 0.46 | 0.32 | NA | NA | **0.58** | 0.48 | 0.33 |
| Amitriptylinoxide 90mg | NA | NA | 0.41 | NA | NA | NA | 0.31 | NA | NA | NA | 0.527 | 0.63 |
| BTX-A 100U | 0.61 | 0.71 | 0.57 | NA | 0.54 | **0.64** | 0.65 | NA | NA | NA | 0.51 | 0.65 |
| BTX-A 150U | NA | 0.48 | NA | NA | NA | 0.52 | NA | NA | NA | NA | NA | 0.65 |
| BTX-A 20U | 0.34 | 0.46 | NA | NA | 0.45 | 0.45 | NA | NA | NA | NA | NA | 0.43 |
| BTX-A 210U | NA | 0.45 | 0.41 | NA | NA | NA | 0.40 | NA | 0.50 | 0.48 | 0.47 | 0.30 |
| BTX-A 420U | NA | 0.45 | 0.38 | NA | NA | NA | 0.40 | NA | 0.48 | 0.52 | **0.531** | 0.28 |
| BTX-A 500U | NA | NA | 0.38 | NA | NA | NA | NA | NA | NA | NA | NA | **0.14** |
| BTX-A 50U | 0.52 | 0.48 | 0.53 | 0.44 | 0.59 | 0.53 | 0.63 | 0.52 | **0.56** | NA | 0.529 | 0.69 |
| BTX-A 86U | NA | 0.47 | NA | NA | NA | 0.53 | NA | NA | NA | NA | NA | 0.71 |
| Citalopram 20mg | NA | 0.39 | NA | NA | NA | 0.51 | NA | NA | NA | 0.48 | NA | 0.79 |
| Ibuprofen 400mg | NA | 0.46 | NA | NA | NA | 0.37 | NA | NA | NA | 0.48 | NA | 0.65 |
| Lidocaine 25ml | NA | NA | **0.75** | NA | NA | NA | **0.87** | NA | NA | NA | NA | **0.82** |
| L-5-Hydroxytryptophan 100mg | NA | 0.42 | 0.50 | NA | NA | 0.62 | 0.49 | NA | NA | NA | NA | 0.56 |
| Mitazapine 30mg | NA | 0.55 | NA | NA | NA | 0.48 | NA | NA | NA | 0.54 | NA | 0.17 |
| Mitazapine 4.5mg | NA | 0.40 | NA | NA | NA | 0.45 | NA | NA | NA | 0.53 | NA | 0.44 |
| Placebo | 0.27 | 0.38 | 0.31 | 0.19 | 0.41 | 0.42 | 0.32 | 0.35 | 0.49 | 0.48 | 0.45 | 0.73 |
| Sodium valproate 1g | 0.69 | NA | 0.68 | NA | **0.60** | NA | 0.54 | NA | NA | NA | NA | 0.41 |
| Tizanidine 12mg | 0..20 | 0.25 | NA | NA | NA | 0.45 | NA | NA | 0.49 | 0.48 | NA | NA |
| Tizanidine 6mg | 0.53 | 0.50 | NA | NA | 0.49 | 0.47 | NA | NA | 0.48 | 0.44 | NA | NA |

**Footnote:** BTX-A, botulinum toxin type-A; CrI, credible interval; SUCRA, surface under the cumulative rank curve; NA, not applicable. Values nearest 1 indicate preferred treatment.

Table S12. The relative effects between different interventions for headache intensity at 4 weeks

| **Treatment** | **Control** | **Mean difference** | **95% CrI** | |
| --- | --- | --- | --- | --- |
| Amitriptyline 100mg | Placebo | -0.44 | -5.63 | 4.88 |
| BTX-A 20U | Placebo | 0.07 | -7.38 | 6.49 |
| BTX-A 50U | Placebo | -1.01 | -7.31 | 4.87 |
| BTX-A 100U | Placebo | -0.48 | -4.18 | 4.08 |
| Sodium valproate 1g | Placebo | -0.97 | -6.61 | 5.87 |
| Tizanidine 6mg | Placebo | -0.16 | -9.87 | 9.42 |
| Tizanidine 12mg | Placebo | 0.73 | -8.57 | 9.90 |
| BTX-A 20U | Amitriptyline 100mg | 0.51 | -8.14 | 8.33 |
| BTX-A 50U | Amitriptyline 100mg | -0.57 | -8.89 | 6.82 |
| BTX-A 100U | Amitriptyline 100mg | -0.04 | -6.52 | 6.97 |
| Sodium valproate 1g | Amitriptyline 100mg | -0.53 | -7.97 | 8.01 |
| Tizanidine 6mg | Amitriptyline 100mg | 0.28 | -10.36 | 10.86 |
| Tizanidine 12mg | Amitriptyline 100mg | 1.17 | -9.34 | 11.67 |
| BTX-A 50U | BTX-A 20U | -1.08 | -9.34 | 7.85 |
| BTX-A 100U | BTX-A 20U | -0.55 | -8.13 | 7.44 |
| Sodium valproate 1g | BTX-A 20U | -1.04 | -9.78 | 9.23 |
| Tizanidine 6mg | BTX-A 20U | -0.23 | -11.73 | 11.54 |
| Tizanidine 12mg | BTX-A 20U | 0.66 | -10.80 | 11.88 |
| BTX-A 100U | BTX-A 50U | 0.53 | -6.65 | 7.84 |
| Sodium valproate 1g | BTX-A 50U | 0.03 | -8.05 | 9.80 |
| Tizanidine 6mg | BTX-A 50U | 0.85 | -10.15 | 11.82 |
| Tizanidine 12mg | BTX-A 50U | 1.74 | -9.15 | 12.49 |
| Sodium valproate 1g | BTX-A 100U | -0.49 | -7.43 | 7.58 |
| Tizanidine 6mg | BTX-A 100U | 0.32 | -9.53 | 10.60 |
| Tizanidine 12mg | BTX-A 100U | 1.21 | -8.54 | 10.74 |
| Tizanidine 6mg | Sodium valproate 1g | 0.81 | -10.98 | 11.62 |
| Tizanidine 12mg | Sodium valproate 1g | 1.71 | -9.30 | 12.03 |
| Tizanidine 12mg | Tizanidine 6mg | 0.89 | -8.89 | 10.64 |

**Footnote:** BTX-A, botulinum toxin type-A; CrI, credible interval.

Table S13. The relative effects between different interventions for headache intensity at 8 weeks

| **Treatment** | **Control** | **Mean difference** | **95% CrI** | |
| --- | --- | --- | --- | --- |
| Amitriptyline 75mg | Placebo | -0.10 | -4.00 | 3.42 |
| Amitriptyline 100mg | Placebo | -0.68 | -4.05 | 3.03 |
| BTX-A 20U | Placebo | 0.21 | -5.08 | 5.94 |
| BTX-A 50U | Placebo | -0.44 | -5.70 | 5.01 |
| BTX-A 86U | Placebo | -0.48 | -5.87 | 5.01 |
| BTX-A 100U | Placebo | -0.85 | -3.17 | 2.06 |
| BTX-A 150U | Placebo | -0.36 | -5.69 | 5.08 |
| Citalopram 20mg | Placebo | -0.34 | -4.08 | 3.20 |
| Ibuprofen 400mg | Placebo | 0.79 | -3.93 | 5.73 |
| L-5-Hydroxytryptophan 100mg | Placebo | -0.83 | -4.39 | 2.86 |
| Mirtazapine 4.5mg | Placebo | 0.20 | -4.57 | 5.01 |
| Mirtazapine 30mg | Placebo | -0.11 | -6.69 | 5.94 |
| Tizanidine 6mg | Placebo | 0.20 | -8.96 | 9.58 |
| Tizanidine 12mg | Placebo | 0.37 | -8.77 | 9.49 |
| Amitriptyline 100mg | Amitriptyline 75mg | -0.58 | -5.68 | 4.70 |
| BTX-A 20U | Amitriptyline 75mg | 0.31 | -5.99 | 7.17 |
| BTX-A 50U | Amitriptyline 75mg | -0.34 | -6.71 | 6.39 |
| BTX-A 86U | Amitriptyline 75mg | -0.38 | -6.56 | 6.00 |
| BTX-A 100U | Amitriptyline 75mg | -0.75 | -4.87 | 3.96 |
| BTX-A 150U | Amitriptyline 75mg | -0.26 | -6.56 | 6.17 |
| Citalopram 20mg | Amitriptyline 75mg | -0.24 | -4.13 | 3.22 |
| Ibuprofen 400mg | Amitriptyline 75mg | 0.89 | -4.74 | 6.43 |
| L-5-Hydroxytryptophan 100mg | Amitriptyline 75mg | -0.73 | -6.06 | 4.49 |
| Mirtazapine 4.5mg | Amitriptyline 75mg | 0.30 | -5.07 | 6.12 |
| Mirtazapine 30mg | Amitriptyline 75mg | -0.01 | -7.27 | 7.19 |
| Tizanidine 6mg | Amitriptyline 75mg | 0.31 | -9.81 | 10.08 |
| Tizanidine 12mg | Amitriptyline 75mg | 0.47 | -9.30 | 10.20 |
| BTX-A 20U | Amitriptyline 100mg | 0.89 | -5.55 | 7.39 |
| BTX-A 50U | Amitriptyline 100mg | 0.24 | -5.93 | 6.78 |
| BTX-A 86U | Amitriptyline 100mg | 0.20 | -5.96 | 6.75 |
| BTX-A 100U | Amitriptyline 100mg | -0.17 | -4.30 | 4.31 |
| BTX-A 150U | Amitriptyline 100mg | 0.32 | -5.80 | 6.66 |
| Citalopram 20mg | Amitriptyline 100mg | 0.34 | -4.76 | 5.41 |
| Ibuprofen 400mg | Amitriptyline 100mg | 1.47 | -4.09 | 7.08 |
| L-5-Hydroxytryptophan 100mg | Amitriptyline 100mg | -0.14 | -5.52 | 4.81 |
| Mirtazapine 4.5mg | Amitriptyline 100mg | 0.88 | -4.91 | 6.85 |
| Mirtazapine 30mg | Amitriptyline 100mg | 0.58 | -6.95 | 7.66 |
| Tizanidine 6mg | Amitriptyline 100mg | 0.89 | -8.74 | 10.90 |
| Tizanidine 12mg | Amitriptyline 100mg | 1.05 | -8.51 | 10.94 |
| BTX-A 50U | BTX-A 20U | -0.65 | -8.37 | 7.41 |
| BTX-A 86U | BTX-A 20U | -0.69 | -8.43 | 6.98 |
| BTX-A 100U | BTX-A 20U | -1.06 | -7.11 | 5.11 |
| BTX-A 150U | BTX-A 20U | -0.57 | -8.51 | 7.22 |
| Citalopram 20mg | BTX-A 20U | -0.55 | -7.44 | 5.78 |
| Ibuprofen 400mg | BTX-A 20U | 0.58 | -6.62 | 7.93 |
| L-5-Hydroxytryptophan 100mg | BTX-A 20U | -1.04 | -7.75 | 5.15 |
| Mirtazapine 4.5mg | BTX-A 20U | -0.01 | -7.54 | 7.20 |
| Mirtazapine 30mg | BTX-A 20U | -0.32 | -8.75 | 8.20 |
| Tizanidine 6mg | BTX-A 20U | 0.00 | -10.60 | 10.59 |
| Tizanidine 12mg | BTX-A 20U | 0.16 | -10.55 | 10.88 |
| BTX-A 86U | BTX-A 50U | -0.04 | -6.56 | 6.21 |
| BTX-A 100U | BTX-A 50U | -0.42 | -5.71 | 5.10 |
| BTX-A 150U | BTX-A 50U | 0.07 | -6.14 | 6.36 |
| Citalopram 20mg | BTX-A 50U | 0.10 | -6.29 | 6.17 |
| Ibuprofen 400mg | BTX-A 50U | 1.23 | -6.11 | 8.43 |
| L-5-Hydroxytryptophan 100mg | BTX-A 50U | -0.39 | -6.95 | 5.83 |
| Mirtazapine 4.5mg | BTX-A 50U | 0.64 | -6.42 | 7.69 |
| Mirtazapine 30mg | BTX-A 50U | 0.33 | -8.30 | 8.63 |
| Tizanidine 6mg | BTX-A 50U | 0.64 | -10.27 | 11.21 |
| Tizanidine 12mg | BTX-A 50U | 0.81 | -10.18 | 11.58 |
| BTX-A 100U | BTX-A 86U | -0.37 | -5.83 | 5.22 |
| BTX-A 150U | BTX-A 86U | 0.12 | -6.10 | 6.57 |
| Citalopram 20mg | BTX-A 86U | 0.14 | -5.99 | 6.21 |
| Ibuprofen 400mg | BTX-A 86U | 1.27 | -6.11 | 8.31 |
| L-5-Hydroxytryptophan 100mg | BTX-A 86U | -0.35 | -6.80 | 5.84 |
| Mirtazapine 4.5mg | BTX-A 86U | 0.68 | -6.71 | 7.97 |
| Mirtazapine 30mg | BTX-A 86U | 0.37 | -7.97 | 8.55 |
| Tizanidine 6mg | BTX-A 86U | 0.68 | -10.48 | 11.79 |
| Tizanidine 12mg | BTX-A 86U | 0.85 | -9.92 | 11.88 |
| BTX-A 150U | BTX-A 100U | 0.49 | -5.00 | 6.00 |
| Citalopram 20mg | BTX-A 100U | 0.51 | -4.52 | 4.75 |
| Ibuprofen 400mg | BTX-A 100U | 1.64 | -3.84 | 6.97 |
| L-5-Hydroxytryptophan 100mg | BTX-A 100U | 0.03 | -4.63 | 4.31 |
| Mirtazapine 4.5mg | BTX-A 100U | 1.05 | -4.10 | 5.97 |
| Mirtazapine 30mg | BTX-A 100U | 0.75 | -6.27 | 7.45 |
| Tizanidine 6mg | BTX-A 100U | 1.06 | -8.75 | 10.70 |
| Tizanidine 12mg | BTX-A 100U | 1.22 | -8.63 | 10.69 |
| Citalopram 20mg | BTX-A 150U | 0.02 | -6.33 | 6.15 |
| Ibuprofen 400mg | BTX-A 150U | 1.15 | -6.13 | 8.18 |
| L-5-Hydroxytryptophan 100mg | BTX-A 150U | -0.46 | -7.18 | 5.88 |
| Mirtazapine 4.5mg | BTX-A 150U | 0.56 | -6.55 | 7.34 |
| Mirtazapine 30mg | BTX-A 150U | 0.26 | -7.81 | 8.13 |
| Tizanidine 6mg | BTX-A 150U | 0.57 | -10.36 | 11.30 |
| Tizanidine 12mg | BTX-A 150U | 0.73 | -10.15 | 11.60 |
| Ibuprofen 400mg | Citalopram 20mg | 1.13 | -4.55 | 7.01 |
| L-5-Hydroxytryptophan 100mg | Citalopram 20mg | -0.49 | -5.83 | 4.93 |
| Mirtazapine 4.5mg | Citalopram 20mg | 0.54 | -5.09 | 6.74 |
| Mirtazapine 30mg | Citalopram 20mg | 0.23 | -7.07 | 7.34 |
| Tizanidine 6mg | Citalopram 20mg | 0.54 | -9.05 | 10.53 |
| Tizanidine 12mg | Citalopram 20mg | 0.71 | -9.01 | 10.54 |
| L-5-Hydroxytryptophan 100mg | Ibuprofen 400mg | -1.62 | -7.43 | 4.25 |
| Mirtazapine 4.5mg | Ibuprofen 400mg | -0.59 | -5.34 | 4.26 |
| Mirtazapine 30mg | Ibuprofen 400mg | -0.90 | -9.37 | 6.79 |
| Tizanidine 6mg | Ibuprofen 400mg | -0.59 | -11.11 | 9.86 |
| Tizanidine 12mg | Ibuprofen 400mg | -0.42 | -10.91 | 9.71 |
| Mirtazapine 4.5mg | L-5-Hydroxytryptophan 100mg | 1.02 | -4.79 | 7.03 |
| Mirtazapine 30mg | L-5-Hydroxytryptophan 100mg | 0.72 | -6.47 | 7.49 |
| Tizanidine 6mg | L-5-Hydroxytryptophan 100mg | 1.03 | -8.97 | 10.69 |
| Tizanidine 12mg | L-5-Hydroxytryptophan 100mg | 1.20 | -8.47 | 11.10 |
| Mirtazapine 30mg | Mirtazapine 4.5mg | -0.30 | -8.56 | 7.64 |
| Tizanidine 6mg | Mirtazapine 4.5mg | 0.01 | -10.39 | 10.16 |
| Tizanidine 12mg | Mirtazapine 4.5mg | 0.17 | -10.16 | 10.43 |
| Tizanidine 6mg | Mirtazapine 30mg | 0.31 | -11.09 | 11.48 |
| Tizanidine 12mg | Mirtazapine 30mg | 0.48 | -10.51 | 11.37 |
| Tizanidine 12mg | Tizanidine 6mg | 0.17 | -9.07 | 9.37 |

**Footnote:** BTX-A, botulinum toxin type-A; CrI, credible interval.

Table S14. The relative effects between different interventions for headache intensity at 12 weeks

| **Treatment** | **Control** | **Mean difference** | **95% CrI** | |
| --- | --- | --- | --- | --- |
| Amitriptyline 75mg | Placebo | 0.93 | -5.06 | 7.37 |
| Amitriptyline 100mg | Placebo | -0.99 | -4.64 | 2.59 |
| Amitriptylinoxide 90mg | Placebo | 0.94 | -4.98 | 6.95 |
| BTX-A 50U | Placebo | -1.52 | -6.14 | 3.07 |
| BTX-A 100U | Placebo | -1.29 | -3.63 | 1.65 |
| BTX-A 210U | Placebo | -0.23 | -3.71 | 3.18 |
| BTX-A 420U | Placebo | -0.23 | -3.56 | 3.13 |
| L-5-Hydroxytryptophan 100mg | Placebo | -0.61 | -4.68 | 2.94 |
| Lidocaine 25ml | Placebo | -3.10 | -6.78 | 1.03 |
| Sodium valproate 1g | Placebo | -0.88 | -5.31 | 3.99 |
| Amitriptyline 100mg | Amitriptyline 75mg | -1.91 | -9.48 | 5.34 |
| Amitriptylinoxide 90mg | Amitriptyline 75mg | 0.01 | -6.71 | 6.74 |
| BTX-A 50U | Amitriptyline 75mg | -2.45 | -10.52 | 5.04 |
| BTX-A 100U | Amitriptyline 75mg | -2.22 | -8.96 | 4.52 |
| BTX-A 210U | Amitriptyline 75mg | -1.15 | -8.22 | 5.66 |
| BTX-A 420U | Amitriptyline 75mg | -1.15 | -8.49 | 5.70 |
| L-5-Hydroxytryptophan 100mg | Amitriptyline 75mg | -1.53 | -8.72 | 5.42 |
| Lidocaine 25ml | Amitriptyline 75mg | -4.02 | -11.20 | 3.04 |
| Sodium valproate 1g | Amitriptyline 75mg | -1.80 | -9.57 | 6.03 |
| Amitriptylinoxide 90mg | Amitriptyline 100mg | 1.93 | -4.97 | 8.53 |
| BTX-A 50U | Amitriptyline 100mg | -0.53 | -6.45 | 5.24 |
| BTX-A 100U | Amitriptyline 100mg | -0.30 | -4.50 | 4.63 |
| BTX-A 210U | Amitriptyline 100mg | 0.76 | -4.72 | 6.01 |
| BTX-A 420U | Amitriptyline 100mg | 0.76 | -4.28 | 5.92 |
| L-5-Hydroxytryptophan 100mg | Amitriptyline 100mg | 0.38 | -5.06 | 5.65 |
| Lidocaine 25ml | Amitriptyline 100mg | -2.11 | -7.36 | 3.83 |
| Sodium valproate 1g | Amitriptyline 100mg | 0.11 | -5.71 | 6.86 |
| BTX-A 50U | Amitriptylinoxide 90mg | -2.46 | -10.00 | 4.91 |
| BTX-A 100U | Amitriptylinoxide 90mg | -2.23 | -8.59 | 4.22 |
| BTX-A 210U | Amitriptylinoxide 90mg | -1.17 | -7.88 | 5.28 |
| BTX-A 420U | Amitriptylinoxide 90mg | -1.17 | -7.98 | 5.30 |
| L-5-Hydroxytryptophan 100mg | Amitriptylinoxide 90mg | -1.55 | -8.11 | 5.18 |
| Lidocaine 25ml | Amitriptylinoxide 90mg | -4.04 | -10.71 | 2.95 |
| Sodium valproate 1g | Amitriptylinoxide 90mg | -1.82 | -8.89 | 5.62 |
| BTX-A 100U | BTX-A 50U | 0.23 | -4.71 | 5.85 |
| BTX-A 210U | BTX-A 50U | 1.29 | -4.23 | 6.94 |
| BTX-A 420U | BTX-A 50U | 1.29 | -4.10 | 6.79 |
| L-5-Hydroxytryptophan 100mg | BTX-A 50U | 0.91 | -4.82 | 6.98 |
| Lidocaine 25ml | BTX-A 50U | -1.58 | -7.25 | 4.65 |
| Sodium valproate 1g | BTX-A 50U | 0.64 | -5.55 | 7.42 |
| BTX-A 210U | BTX-A 100U | 1.06 | -3.53 | 4.99 |
| BTX-A 420U | BTX-A 100U | 1.06 | -3.50 | 5.01 |
| L-5-Hydroxytryptophan 100mg | BTX-A 100U | 0.68 | -4.33 | 4.82 |
| Lidocaine 25ml | BTX-A 100U | -1.81 | -6.73 | 2.94 |
| Sodium valproate 1g | BTX-A 100U | 0.41 | -4.83 | 5.94 |
| BTX-A 420U | BTX-A 210U | 0.00 | -3.69 | 3.51 |
| L-5-Hydroxytryptophan 100mg | BTX-A 210U | -0.38 | -5.31 | 4.25 |
| Lidocaine 25ml | BTX-A 210U | -2.87 | -7.94 | 2.42 |
| Sodium valproate 1g | BTX-A 210U | -0.65 | -6.04 | 5.08 |
| L-5-Hydroxytryptophan 100mg | BTX-A 420U | -0.38 | -5.42 | 4.15 |
| Lidocaine 25ml | BTX-A 420U | -2.87 | -7.83 | 2.80 |
| Sodium valproate 1g | BTX-A 420U | -0.65 | -6.10 | 5.33 |
| Lidocaine 25ml | L-5-Hydroxytryptophan 100mg | -2.49 | -7.56 | 3.23 |
| Sodium valproate 1g | L-5-Hydroxytryptophan 100mg | -0.27 | -5.75 | 5.94 |
| Sodium valproate 1g | Lidocaine 25ml | 2.22 | -3.35 | 8.17 |

Table S15. The relative effects between different interventions for headache intensity at 24 weeks

| **Treatment** | **Control** | **Mean difference** | **95% CrI** | |
| --- | --- | --- | --- | --- |
| Amitriptyline 100mg | Placebo | -0.85 | -8.17 | 6.61 |
| BTX-A 50U | Placebo | -0.58 | -8.37 | 7.25 |
| BTX-A 50U | Amitriptyline 100mg | 0.27 | -9.38 | 10.76 |

**Footnote:** BTX-A, botulinum toxin type-A; CrI, credible interval.

Table S16. The relative effects between different interventions for headache duration at 4 weeks

| **Treatment** | **Control** | **Mean difference** | **95% CrI** | |
| --- | --- | --- | --- | --- |
| BTX-A 50U | Placebo | -0.76 | -8.16 | 6.48 |
| BTX-A 210U | Placebo | 0.04 | -12.17 | 12.03 |
| BTX-A 420U | Placebo | 0.12 | -10.49 | 10.94 |
| Tizanidine 6mg | Placebo | 0.27 | -10.77 | 10.88 |
| Tizanidine 12mg | Placebo | 0.14 | -10.42 | 11.25 |
| BTX-A 210U | BTX-A 50U | 0.79 | -13.09 | 15.17 |
| BTX-A 420U | BTX-A 50U | 0.88 | -11.87 | 13.55 |
| Tizanidine 6mg | BTX-A 50U | 1.03 | -11.65 | 13.94 |
| Tizanidine 12mg | BTX-A 50U | 0.90 | -11.78 | 13.79 |
| BTX-A 420U | BTX-A 210U | 0.08 | -13.77 | 13.70 |
| Tizanidine 6mg | BTX-A 210U | 0.24 | -15.93 | 16.28 |
| Tizanidine 12mg | BTX-A 210U | 0.10 | -16.30 | 16.64 |
| Tizanidine 6mg | BTX-A 420U | 0.15 | -15.33 | 15.14 |
| Tizanidine 12mg | BTX-A 420U | 0.02 | -14.96 | 15.16 |
| Tizanidine 12mg | Tizanidine 6mg | -0.13 | -10.79 | 10.93 |

**Footnote:** BTX-A, botulinum toxin type-A; CrI, credible interval.

Table S17. The relative effects between different interventions for headache duration at 8 weeks

| **Treatment** | **Control** | **Mean difference** | **95% CrI** | |
| --- | --- | --- | --- | --- |
| Amitriptyline 75mg | Placebo | -1.05 | -7.36 | 5.65 |
| BTX-A 210U | Placebo | 0.22 | -11.68 | 12.42 |
| BTX-A 420U | Placebo | -0.45 | -11.28 | 11.12 |
| Citalopram 20mg | Placebo | -0.05 | -6.65 | 6.45 |
| Ibuprofen 400mg | Placebo | 0.38 | -15.89 | 16.92 |
| Mirtazapine 4.5mg | Placebo | -0.98 | -16.75 | 14.61 |
| Mirtazapine 30mg | Placebo | -1.11 | -16.22 | 13.38 |
| Tizanidine 6mg | Placebo | 0.84 | -9.35 | 11.39 |
| Tizanidine 12mg | Placebo | 0.21 | -10.86 | 11.17 |
| BTX-A 210U | Amitriptyline 75mg | 1.27 | -12.09 | 14.96 |
| BTX-A 420U | Amitriptyline 75mg | 0.60 | -11.77 | 13.52 |
| Citalopram 20mg | Amitriptyline 75mg | 1.00 | -6.25 | 7.81 |
| Ibuprofen 400mg | Amitriptyline 75mg | 1.43 | -16.19 | 19.00 |
| Mirtazapine 4.5mg | Amitriptyline 75mg | 0.06 | -16.84 | 17.04 |
| Mirtazapine 30mg | Amitriptyline 75mg | -0.06 | -16.19 | 16.01 |
| Tizanidine 6mg | Amitriptyline 75mg | 1.89 | -10.75 | 14.49 |
| Tizanidine 12mg | Amitriptyline 75mg | 1.26 | -11.55 | 13.92 |
| BTX-A 420U | BTX-A 210U | -0.67 | -13.85 | 12.76 |
| Citalopram 20mg | BTX-A 210U | -0.27 | -14.15 | 12.96 |
| Ibuprofen 400mg | BTX-A 210U | 0.16 | -20.38 | 20.40 |
| Mirtazapine 4.5mg | BTX-A 210U | -1.21 | -21.23 | 18.82 |
| Mirtazapine 30mg | BTX-A 210U | -1.33 | -20.28 | 17.97 |
| Tizanidine 6mg | BTX-A 210U | 0.62 | -15.51 | 16.90 |
| Tizanidine 12mg | BTX-A 210U | -0.01 | -16.10 | 16.65 |
| Citalopram 20mg | BTX-A 420U | 0.40 | -12.45 | 12.80 |
| Ibuprofen 400mg | BTX-A 420U | 0.83 | -19.57 | 20.98 |
| Mirtazapine 4.5mg | BTX-A 420U | -0.54 | -20.37 | 18.72 |
| Mirtazapine 30mg | BTX-A 420U | -0.66 | -19.11 | 17.72 |
| Tizanidine 6mg | BTX-A 420U | 1.29 | -14.02 | 15.99 |
| Tizanidine 12mg | BTX-A 420U | 0.66 | -14.98 | 16.36 |
| Ibuprofen 400mg | Citalopram 20mg | 0.43 | -17.49 | 18.54 |
| Mirtazapine 4.5mg | Citalopram 20mg | -0.94 | -18.33 | 16.54 |
| Mirtazapine 30mg | Citalopram 20mg | -1.06 | -17.01 | 15.11 |
| Tizanidine 6mg | Citalopram 20mg | 0.89 | -11.46 | 13.37 |
| Tizanidine 12mg | Citalopram 20mg | 0.26 | -13.09 | 12.86 |
| Mirtazapine 4.5mg | Ibuprofen 400mg | -1.37 | -21.97 | 19.47 |
| Mirtazapine 30mg | Ibuprofen 400mg | -1.49 | -23.50 | 21.09 |
| Tizanidine 6mg | Ibuprofen 400mg | 0.46 | -19.55 | 19.91 |
| Tizanidine 12mg | Ibuprofen 400mg | -0.17 | -19.92 | 19.06 |
| Mirtazapine 30mg | Mirtazapine 4.5mg | -0.12 | -21.65 | 21.40 |
| Tizanidine 6mg | Mirtazapine 4.5mg | 1.82 | -16.71 | 20.90 |
| Tizanidine 12mg | Mirtazapine 4.5mg | 1.20 | -17.65 | 20.27 |
| Tizanidine 6mg | Mirtazapine 30mg | 1.95 | -15.64 | 20.30 |
| Tizanidine 12mg | Mirtazapine 30mg | 1.32 | -17.49 | 19.81 |
| Tizanidine 12mg | Tizanidine 6mg | -0.63 | -11.43 | 9.71 |

**Footnote:** BTX-A, botulinum toxin type-A; CrI, credible interval.

Table S18. The relative effects between different interventions for headache duration at 12 weeks

| **Treatment** | **Control** | **Mean difference** | **95% CrI** | |
| --- | --- | --- | --- | --- |
| Amitriptyline 75mg | Placebo | -0.25 | -12.72 | 11.85 |
| Amitriptylinoxide 90mg | Placebo | -0.93 | -13.99 | 12.70 |
| BTX-A 50U | Placebo | -0.84 | -8.18 | 6.69 |
| BTX-A 100U | Placebo | -0.62 | -13.38 | 12.08 |
| BTX-A 210U | Placebo | -0.04 | -12.74 | 12.07 |
| BTX-A 420U | Placebo | -0.93 | -11.71 | 10.11 |
| Amitriptylinoxide 90mg | Amitriptyline 75mg | -0.68 | -15.96 | 14.81 |
| BTX-A 50U | Amitriptyline 75mg | -0.59 | -14.63 | 13.24 |
| BTX-A 100U | Amitriptyline 75mg | -0.38 | -17.99 | 16.95 |
| BTX-A 210U | Amitriptyline 75mg | 0.21 | -17.67 | 17.95 |
| BTX-A 420U | Amitriptyline 75mg | -0.68 | -17.64 | 15.38 |
| BTX-A 50U | Amitriptylinoxide 90mg | 0.09 | -14.73 | 14.82 |
| BTX-A 100U | Amitriptylinoxide 90mg | 0.31 | -18.38 | 18.49 |
| BTX-A 210U | Amitriptylinoxide 90mg | 0.89 | -17.50 | 19.10 |
| BTX-A 420U | Amitriptylinoxide 90mg | 0.00 | -17.14 | 16.78 |
| BTX-A 100U | BTX-A 50U | 0.22 | -14.30 | 15.41 |
| BTX-A 210U | BTX-A 50U | 0.80 | -13.69 | 14.53 |
| BTX-A 420U | BTX-A 50U | -0.09 | -13.11 | 12.89 |
| BTX-A 210U | BTX-A 100U | 0.58 | -17.04 | 18.13 |
| BTX-A 420U | BTX-A 100U | -0.31 | -16.66 | 16.23 |
| BTX-A 420U | BTX-A 210U | -0.89 | -14.54 | 12.97 |

**Footnote:** BTX-A, botulinum toxin type-A; CrI, credible interval.

Table S19. The relative effects between different interventions for adverse event rate

| **Treatment** | **Control** | **Odds Ratio** | **95% CrI** | |
| --- | --- | --- | --- | --- |
| Amitriptyline 25mg | Placebo | 2.92 | 0.20 | 55.35 |
| Amitriptyline 75mg | Placebo | 3.99 | 0.98 | 17.12 |
| **Amitriptyline 100mg** | **Placebo** | **9.53** | **1.15** | **65.77** |
| Amitriptylinoxide 90mg | Placebo | 1.32 | 0.21 | 8.95 |
| BTX-A 20U | Placebo | 2.81 | 0.35 | 22.70 |
| BTX-A 50U | Placebo | 1.11 | 0.35 | 4.38 |
| BTX-A 86U | Placebo | 1.03 | 0.19 | 6.12 |
| BTX-A 100U | Placebo | 1.22 | 0.36 | 4.31 |
| BTX-A 150U | Placebo | 1.23 | 0.21 | 7.71 |
| BTX-A 210U | Placebo | 5.16 | 0.54 | 46.66 |
| BTX-A 420U | Placebo | 5.76 | 0.62 | 53.46 |
| **BTX-A 500U** | **Placebo** | **19.04** | **1.20** | **568.70** |
| Citalopram 20mg | Placebo | 0.71 | 0.10 | 4.83 |
| Ibuprofen 400mg | Placebo | 1.21 | 0.15 | 10.00 |
| L-5-Hydroxytryptophan 100mg | Placebo | 1.73 | 0.16 | 18.86 |
| Lidocaine 25ml | Placebo | 0.58 | 0.07 | 5.02 |
| Mirtazapine 4.5mg | Placebo | 2.68 | 0.31 | 24.42 |
| Mirtazapine 30mg | Placebo | 14.86 | 0.91 | 467.68 |
| Sodium valproate 1g | Placebo | 3.73 | 0.19 | 133.76 |
| Amitriptyline 75mg | Amitriptyline 25mg | 1.37 | 0.06 | 25.83 |
| Amitriptyline 100mg | Amitriptyline 25mg | 3.26 | 0.11 | 80.73 |
| Amitriptylinoxide 90mg | Amitriptyline 25mg | 0.45 | 0.02 | 12.21 |
| BTX-A 20U | Amitriptyline 25mg | 0.96 | 0.03 | 29.15 |
| BTX-A 50U | Amitriptyline 25mg | 0.38 | 0.02 | 8.58 |
| BTX-A 86U | Amitriptyline 25mg | 0.35 | 0.01 | 9.02 |
| BTX-A 100U | Amitriptyline 25mg | 0.42 | 0.02 | 8.40 |
| BTX-A 150U | Amitriptyline 25mg | 0.42 | 0.02 | 12.44 |
| BTX-A 210U | Amitriptyline 25mg | 1.77 | 0.05 | 50.86 |
| BTX-A 420U | Amitriptyline 25mg | 1.97 | 0.05 | 60.44 |
| BTX-A 500U | Amitriptyline 25mg | 6.52 | 0.10 | 473.10 |
| Citalopram 20mg | Amitriptyline 25mg | 0.24 | 0.01 | 6.51 |
| Ibuprofen 400mg | Amitriptyline 25mg | 0.41 | 0.01 | 10.80 |
| L-5-Hydroxytryptophan 100mg | Amitriptyline 25mg | 0.59 | 0.01 | 21.35 |
| Lidocaine 25ml | Amitriptyline 25mg | 0.20 | 0.01 | 6.16 |
| Mirtazapine 4.5mg | Amitriptyline 25mg | 0.92 | 0.02 | 30.58 |
| Mirtazapine 30mg | Amitriptyline 25mg | 5.08 | 0.10 | 379.99 |
| Sodium valproate 1g | Amitriptyline 25mg | 1.28 | 0.02 | 115.14 |
| Amitriptyline 100mg | Amitriptyline 75mg | 2.39 | 0.19 | 25.14 |
| Amitriptylinoxide 90mg | Amitriptyline 75mg | 0.33 | 0.05 | 2.14 |
| BTX-A 20U | Amitriptyline 75mg | 0.70 | 0.06 | 8.95 |
| BTX-A 50U | Amitriptyline 75mg | 0.28 | 0.04 | 2.04 |
| BTX-A 86U | Amitriptyline 75mg | 0.26 | 0.03 | 2.69 |
| BTX-A 100U | Amitriptyline 75mg | 0.31 | 0.05 | 1.99 |
| BTX-A 150U | Amitriptyline 75mg | 0.31 | 0.03 | 3.01 |
| BTX-A 210U | Amitriptyline 75mg | 1.29 | 0.08 | 16.13 |
| BTX-A 420U | Amitriptyline 75mg | 1.44 | 0.10 | 18.69 |
| BTX-A 500U | Amitriptyline 75mg | 4.77 | 0.21 | 194.78 |
| Citalopram 20mg | Amitriptyline 75mg | 0.18 | 0.03 | 1.13 |
| Ibuprofen 400mg | Amitriptyline 75mg | 0.30 | 0.02 | 3.99 |
| L-5-Hydroxytryptophan 100mg | Amitriptyline 75mg | 0.43 | 0.03 | 6.81 |
| Lidocaine 25ml | Amitriptyline 75mg | 0.15 | 0.01 | 2.05 |
| Mirtazapine 4.5mg | Amitriptyline 75mg | 0.67 | 0.05 | 8.54 |
| Mirtazapine 30mg | Amitriptyline 75mg | 3.72 | 0.16 | 122.26 |
| Sodium valproate 1g | Amitriptyline 75mg | 0.94 | 0.03 | 46.08 |
| Amitriptylinoxide 90mg | Amitriptyline 100mg | 0.14 | 0.01 | 2.07 |
| BTX-A 20U | Amitriptyline 100mg | 0.29 | 0.02 | 5.42 |
| BTX-A 50U | Amitriptyline 100mg | 0.12 | 0.01 | 1.53 |
| BTX-A 86U | Amitriptyline 100mg | 0.11 | 0.01 | 1.96 |
| BTX-A 100U | Amitriptyline 100mg | 0.13 | 0.01 | 1.57 |
| BTX-A 150U | Amitriptyline 100mg | 0.13 | 0.01 | 2.26 |
| BTX-A 210U | Amitriptyline 100mg | 0.54 | 0.03 | 11.02 |
| BTX-A 420U | Amitriptyline 100mg | 0.60 | 0.03 | 12.49 |
| BTX-A 500U | Amitriptyline 100mg | 2.00 | 0.07 | 97.38 |
| Citalopram 20mg | Amitriptyline 100mg | 0.07 | 0.01 | 1.26 |
| Ibuprofen 400mg | Amitriptyline 100mg | 0.13 | 0.01 | 2.18 |
| L-5-Hydroxytryptophan 100mg | Amitriptyline 100mg | 0.18 | 0.01 | 4.24 |
| Lidocaine 25ml | Amitriptyline 100mg | 0.06 | 0.004 | 1.45 |
| Mirtazapine 4.5mg | Amitriptyline 100mg | 0.28 | 0.02 | 7.17 |
| Mirtazapine 30mg | Amitriptyline 100mg | 1.56 | 0.05 | 78.98 |
| Sodium valproate 1g | Amitriptyline 100mg | 0.39 | 0.01 | 26.64 |
| BTX-A 20U | Amitriptylinoxide 90mg | 2.13 | 0.14 | 32.45 |
| BTX-A 50U | Amitriptylinoxide 90mg | 0.84 | 0.08 | 9.82 |
| BTX-A 86U | Amitriptylinoxide 90mg | 0.78 | 0.06 | 10.78 |
| BTX-A 100U | Amitriptylinoxide 90mg | 0.93 | 0.10 | 8.27 |
| BTX-A 150U | Amitriptylinoxide 90mg | 0.93 | 0.06 | 15.64 |
| BTX-A 210U | Amitriptylinoxide 90mg | 3.91 | 0.21 | 70.19 |
| BTX-A 420U | Amitriptylinoxide 90mg | 4.36 | 0.21 | 83.58 |
| BTX-A 500U | Amitriptylinoxide 90mg | 14.43 | 0.42 | 691.60 |
| Citalopram 20mg | Amitriptylinoxide 90mg | 0.54 | 0.05 | 5.86 |
| Ibuprofen 400mg | Amitriptylinoxide 90mg | 0.92 | 0.05 | 14.20 |
| L-5-Hydroxytryptophan 100mg | Amitriptylinoxide 90mg | 1.31 | 0.06 | 27.80 |
| Lidocaine 25ml | Amitriptylinoxide 90mg | 0.44 | 0.02 | 7.55 |
| Mirtazapine 4.5mg | Amitriptylinoxide 90mg | 2.03 | 0.11 | 29.71 |
| Mirtazapine 30mg | Amitriptylinoxide 90mg | 11.26 | 0.39 | 514.09 |
| Sodium valproate 1g | Amitriptylinoxide 90mg | 2.83 | 0.09 | 138.97 |
| BTX-A 50U | BTX-A 20U | 0.39 | 0.04 | 4.91 |
| BTX-A 86U | BTX-A 20U | 0.37 | 0.03 | 5.74 |
| BTX-A 100U | BTX-A 20U | 0.43 | 0.04 | 5.00 |
| BTX-A 150U | BTX-A 20U | 0.44 | 0.03 | 7.67 |
| BTX-A 210U | BTX-A 20U | 1.84 | 0.09 | 36.59 |
| BTX-A 420U | BTX-A 20U | 2.05 | 0.08 | 42.02 |
| BTX-A 500U | BTX-A 20U | 6.78 | 0.20 | 345.24 |
| Citalopram 20mg | BTX-A 20U | 0.25 | 0.01 | 3.97 |
| Ibuprofen 400mg | BTX-A 20U | 0.43 | 0.02 | 8.34 |
| L-5-Hydroxytryptophan 100mg | BTX-A 20U | 0.62 | 0.02 | 16.48 |
| Lidocaine 25ml | BTX-A 20U | 0.21 | 0.01 | 3.95 |
| Mirtazapine 4.5mg | BTX-A 20U | 0.96 | 0.05 | 18.92 |
| Mirtazapine 30mg | BTX-A 20U | 5.29 | 0.16 | 292.82 |
| Sodium valproate 1g | BTX-A 20U | 1.33 | 0.04 | 83.05 |
| BTX-A 86U | BTX-A 50U | 0.92 | 0.16 | 5.88 |
| BTX-A 100U | BTX-A 50U | 1.10 | 0.22 | 4.94 |
| BTX-A 150U | BTX-A 50U | 1.11 | 0.16 | 7.57 |
| BTX-A 210U | BTX-A 50U | 4.65 | 0.30 | 64.66 |
| BTX-A 420U | BTX-A 50U | 5.19 | 0.32 | 70.06 |
| BTX-A 500U | BTX-A 50U | 17.17 | 0.78 | 665.67 |
| Citalopram 20mg | BTX-A 50U | 0.64 | 0.06 | 5.82 |
| Ibuprofen 400mg | BTX-A 50U | 1.09 | 0.09 | 10.92 |
| L-5-Hydroxytryptophan 100mg | BTX-A 50U | 1.56 | 0.10 | 22.61 |
| Lidocaine 25ml | BTX-A 50U | 0.53 | 0.04 | 6.01 |
| Mirtazapine 4.5mg | BTX-A 50U | 2.42 | 0.18 | 27.28 |
| Mirtazapine 30mg | BTX-A 50U | 13.40 | 0.56 | 430.50 |
| Sodium valproate 1g | BTX-A 50U | 3.37 | 0.12 | 160.79 |
| BTX-A 100U | BTX-A 86U | 1.19 | 0.17 | 7.57 |
| BTX-A 150U | BTX-A 86U | 1.20 | 0.17 | 9.04 |
| BTX-A 210U | BTX-A 86U | 5.03 | 0.27 | 83.19 |
| BTX-A 420U | BTX-A 86U | 5.61 | 0.28 | 96.79 |
| BTX-A 500U | BTX-A 86U | 18.57 | 0.62 | 784.00 |
| Citalopram 20mg | BTX-A 86U | 0.70 | 0.05 | 7.80 |
| Ibuprofen 400mg | BTX-A 86U | 1.18 | 0.07 | 16.52 |
| L-5-Hydroxytryptophan 100mg | BTX-A 86U | 1.69 | 0.09 | 32.63 |
| Lidocaine 25ml | BTX-A 86U | 0.57 | 0.03 | 8.67 |
| Mirtazapine 4.5mg | BTX-A 86U | 2.62 | 0.15 | 40.16 |
| Mirtazapine 30mg | BTX-A 86U | 14.49 | 0.54 | 654.45 |
| Sodium valproate 1g | BTX-A 86U | 3.64 | 0.12 | 192.95 |
| BTX-A 150U | BTX-A 100U | 1.00 | 0.17 | 7.06 |
| BTX-A 210U | BTX-A 100U | 4.23 | 0.35 | 48.26 |
| BTX-A 420U | BTX-A 100U | 4.72 | 0.34 | 55.62 |
| BTX-A 500U | BTX-A 100U | 15.59 | 0.75 | 615.61 |
| Citalopram 20mg | BTX-A 100U | 0.58 | 0.06 | 5.06 |
| Ibuprofen 400mg | BTX-A 100U | 0.99 | 0.08 | 10.91 |
| L-5-Hydroxytryptophan 100mg | BTX-A 100U | 1.42 | 0.09 | 20.57 |
| Lidocaine 25ml | BTX-A 100U | 0.48 | 0.04 | 5.96 |
| Mirtazapine 4.5mg | BTX-A 100U | 2.20 | 0.19 | 23.90 |
| Mirtazapine 30mg | BTX-A 100U | 12.17 | 0.55 | 431.02 |
| Sodium valproate 1g | BTX-A 100U | 3.06 | 0.12 | 133.59 |
| BTX-A 210U | BTX-A 150U | 4.21 | 0.19 | 66.59 |
| BTX-A 420U | BTX-A 150U | 4.70 | 0.23 | 78.44 |
| BTX-A 500U | BTX-A 150U | 15.53 | 0.47 | 730.44 |
| Citalopram 20mg | BTX-A 150U | 0.58 | 0.04 | 7.65 |
| Ibuprofen 400mg | BTX-A 150U | 0.99 | 0.05 | 14.40 |
| L-5-Hydroxytryptophan 100mg | BTX-A 150U | 1.41 | 0.06 | 31.31 |
| Lidocaine 25ml | BTX-A 150U | 0.48 | 0.03 | 8.29 |
| Mirtazapine 4.5mg | BTX-A 150U | 2.19 | 0.12 | 34.53 |
| Mirtazapine 30mg | BTX-A 150U | 12.12 | 0.41 | 532.38 |
| Sodium valproate 1g | BTX-A 150U | 3.04 | 0.09 | 171.47 |
| BTX-A 420U | BTX-A 210U | 1.12 | 0.12 | 10.57 |
| BTX-A 500U | BTX-A 210U | 3.69 | 0.11 | 190.02 |
| Citalopram 20mg | BTX-A 210U | 0.14 | 0.01 | 2.86 |
| Ibuprofen 400mg | BTX-A 210U | 0.23 | 0.01 | 5.03 |
| L-5-Hydroxytryptophan 100mg | BTX-A 210U | 0.34 | 0.01 | 9.01 |
| Lidocaine 25ml | BTX-A 210U | 0.11 | 0.01 | 2.86 |
| Mirtazapine 4.5mg | BTX-A 210U | 0.52 | 0.02 | 12.59 |
| Mirtazapine 30mg | BTX-A 210U | 2.88 | 0.09 | 205.09 |
| Sodium valproate 1g | BTX-A 210U | 0.72 | 0.02 | 45.47 |
| BTX-A 500U | BTX-A 420U | 3.31 | 0.09 | 182.68 |
| Citalopram 20mg | BTX-A 420U | 0.12 | 0.01 | 2.42 |
| Ibuprofen 400mg | BTX-A 420U | 0.21 | 0.01 | 4.62 |
| L-5-Hydroxytryptophan 100mg | BTX-A 420U | 0.30 | 0.01 | 9.48 |
| Lidocaine 25ml | BTX-A 420U | 0.10 | 0.004 | 2.47 |
| Mirtazapine 4.5mg | BTX-A 420U | 0.47 | 0.02 | 10.92 |
| Mirtazapine 30mg | BTX-A 420U | 2.58 | 0.08 | 144.63 |
| Sodium valproate 1g | BTX-A 420U | 0.65 | 0.01 | 52.17 |
| Citalopram 20mg | BTX-A 500U | 0.04 | 0.001 | 1.02 |
| Ibuprofen 400mg | BTX-A 500U | 0.06 | 0.001 | 2.43 |
| L-5-Hydroxytryptophan 100mg | BTX-A 500U | 0.09 | 0.002 | 3.65 |
| Lidocaine 25ml | BTX-A 500U | 0.03 | 0.001 | 1.15 |
| Mirtazapine 4.5mg | BTX-A 500U | 0.14 | 0.003 | 5.42 |
| Mirtazapine 30mg | BTX-A 500U | 0.78 | 0.01 | 61.87 |
| Sodium valproate 1g | BTX-A 500U | 0.20 | 0.002 | 17.06 |
| Ibuprofen 400mg | Citalopram 20mg | 1.69 | 0.10 | 30.65 |
| L-5-Hydroxytryptophan 100mg | Citalopram 20mg | 2.43 | 0.11 | 55.49 |
| Lidocaine 25ml | Citalopram 20mg | 0.82 | 0.05 | 14.30 |
| Mirtazapine 4.5mg | Citalopram 20mg | 3.76 | 0.25 | 70.46 |
| Mirtazapine 30mg | Citalopram 20mg | 20.81 | 0.68 | 880.52 |
| Sodium valproate 1g | Citalopram 20mg | 5.23 | 0.17 | 288.30 |
| L-5-Hydroxytryptophan 100mg | Ibuprofen 400mg | 1.44 | 0.06 | 34.12 |
| Lidocaine 25ml | Ibuprofen 400mg | 0.48 | 0.03 | 9.47 |
| Mirtazapine 4.5mg | Ibuprofen 400mg | 2.22 | 0.28 | 19.28 |
| Mirtazapine 30mg | Ibuprofen 400mg | 12.30 | 0.31 | 733.91 |
| Sodium valproate 1g | Ibuprofen 400mg | 3.09 | 0.08 | 200.11 |
| Lidocaine 25ml | L-5-Hydroxytryptophan 100mg | 0.34 | 0.01 | 9.25 |
| Mirtazapine 4.5mg | L-5-Hydroxytryptophan 100mg | 1.55 | 0.06 | 39.83 |
| Mirtazapine 30mg | L-5-Hydroxytryptophan 100mg | 8.57 | 0.21 | 472.20 |
| Sodium valproate 1g | L-5-Hydroxytryptophan 100mg | 2.15 | 0.05 | 185.09 |
| Mirtazapine 4.5mg | Lidocaine 25ml | 4.60 | 0.22 | 103.92 |
| Mirtazapine 30mg | Lidocaine 25ml | 25.48 | 0.71 | 1326.34 |
| Sodium valproate 1g | Lidocaine 25ml | 6.40 | 0.16 | 458.31 |
| Mirtazapine 30mg | Mirtazapine 4.5mg | 5.54 | 0.12 | 248.68 |
| Sodium valproate 1g | Mirtazapine 4.5mg | 1.39 | 0.03 | 81.08 |
| Sodium valproate 1g | Mirtazapine 30mg | 0.25 | 0.002 | 27.72 |

**Footnote:** BTX-A, botulinum toxin type-A; CrI, credible interval.

Figure S1. Risk of bias of included RCTs


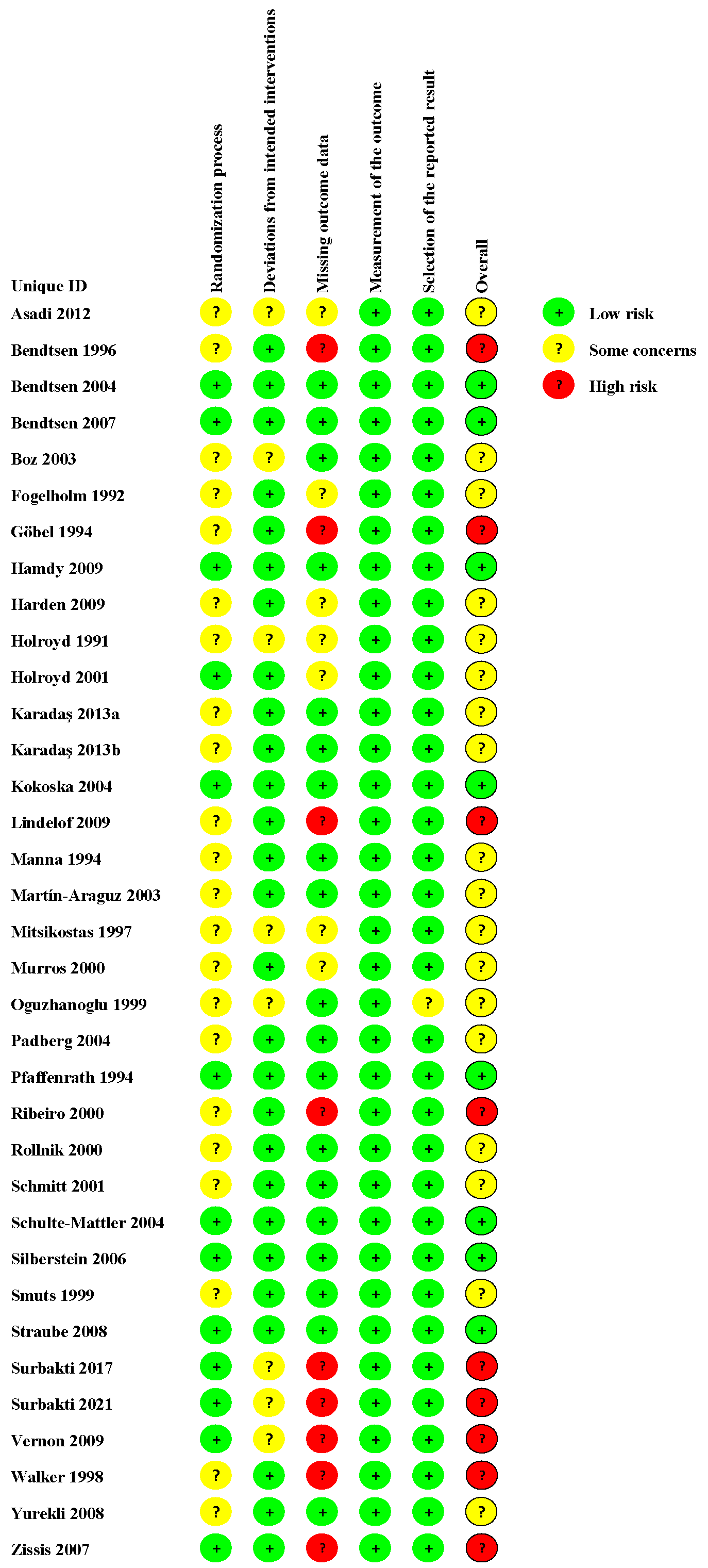


Figure S2. The “dev-dev” plots of random-effects consistency and inconsistency models for NMA of headache days per month


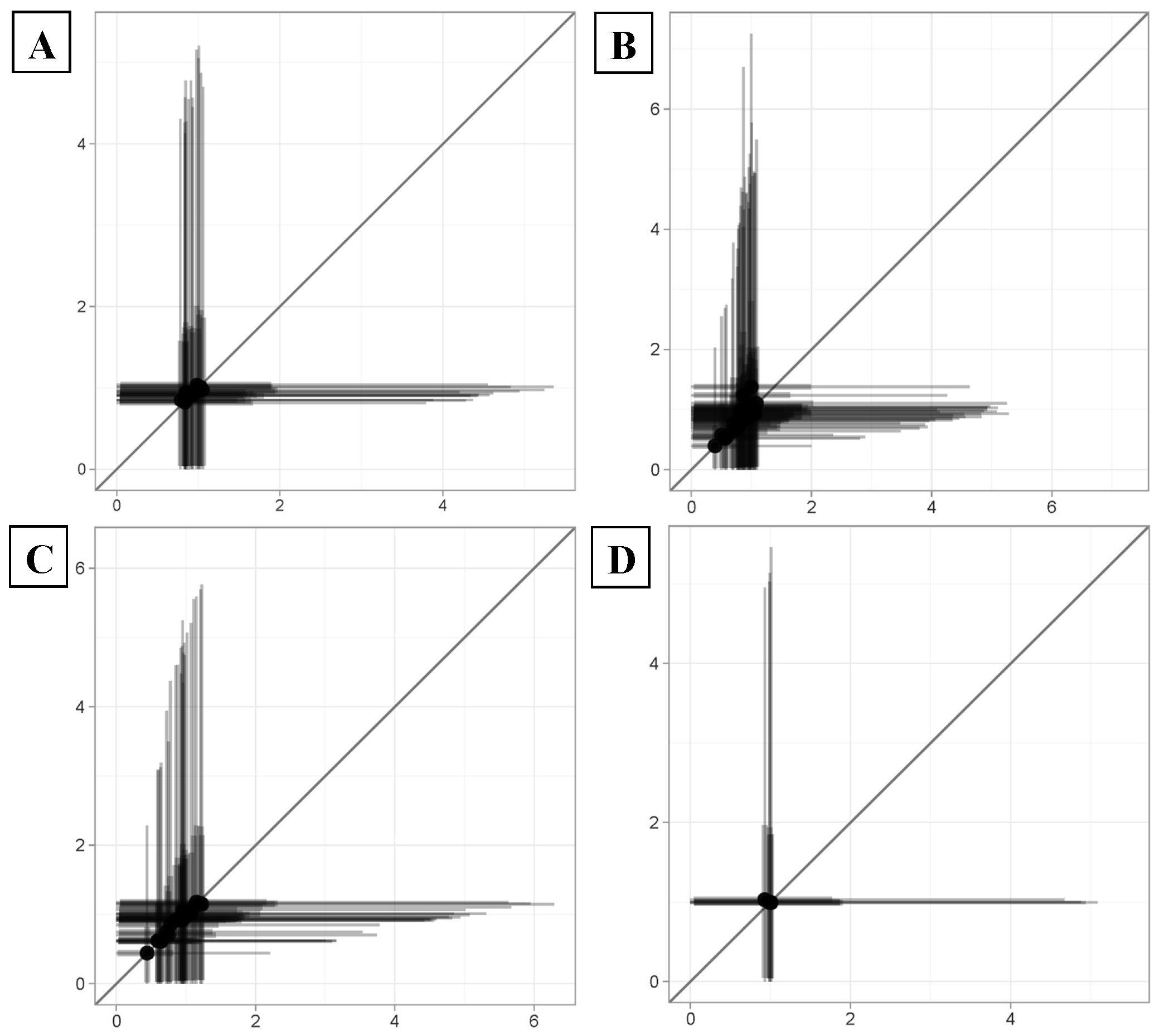


**Footnote:** NMA, network meta-analysis. A: 4 weeks; B: 8 weeks; C: 12 weeks; D: 24 weeks. The horizontal coordinate represents residual deviance of consistency model and the vertical coordinate represents residual deviance of inconsistency model. All points lie roughly on the line of equality, indicating that there is no evidence for inconsistency.

Figure S3. The “dev-dev” plots of random-effects consistency and inconsistency models for NMA of headache intensity


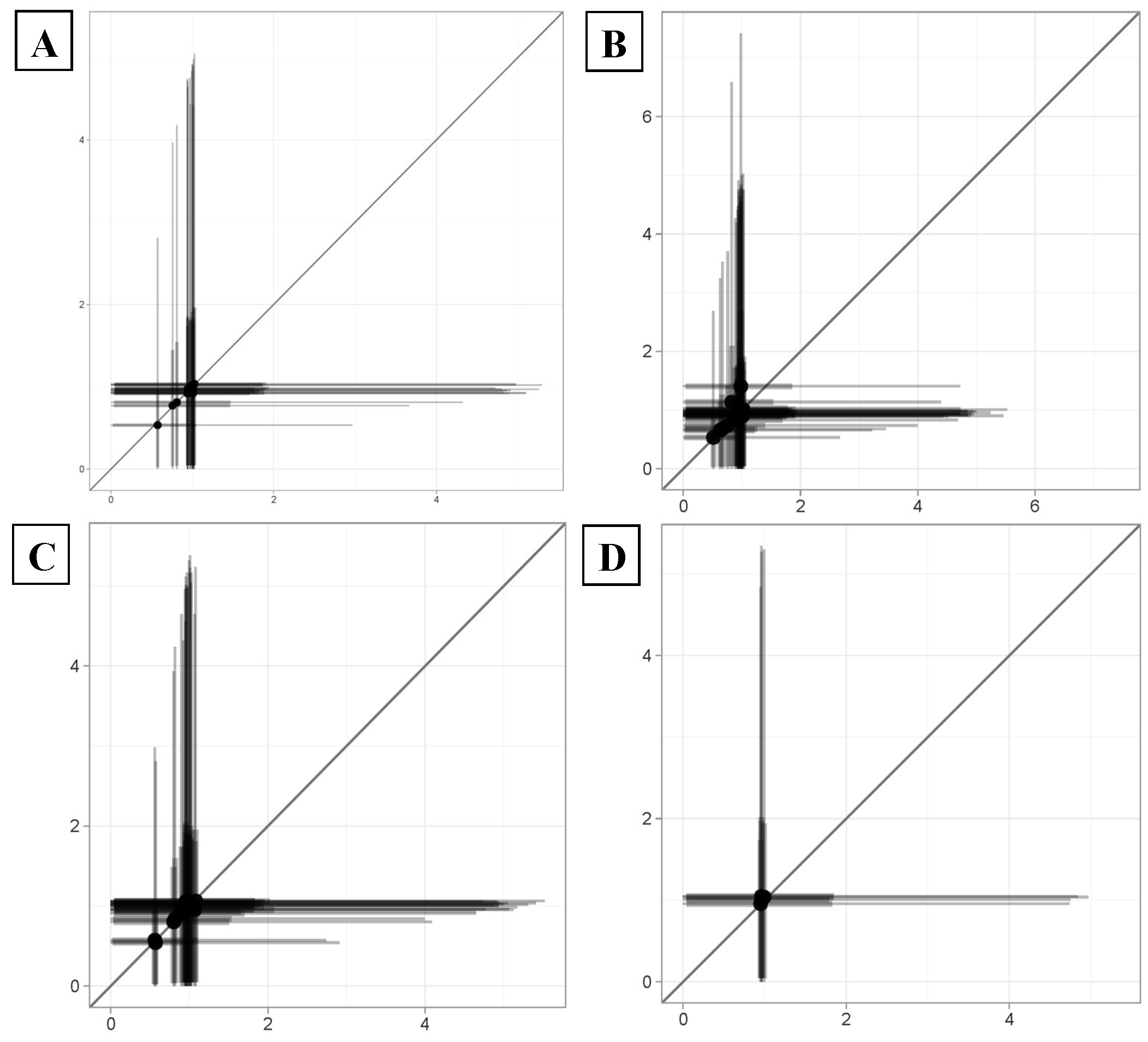


**Footnote:** NMA, network meta-analysis. A: 4 weeks; B: 8 weeks; C: 12 weeks; D: 24 weeks. The horizontal coordinate represents residual deviance of consistency model and the vertical coordinate represents residual deviance of inconsistency model. All points lie roughly on the line of equality, indicating that there is no evidence for inconsistency.

Figure S4. The “dev-dev” plots of random-effects consistency and inconsistency models for NMA of headache duration


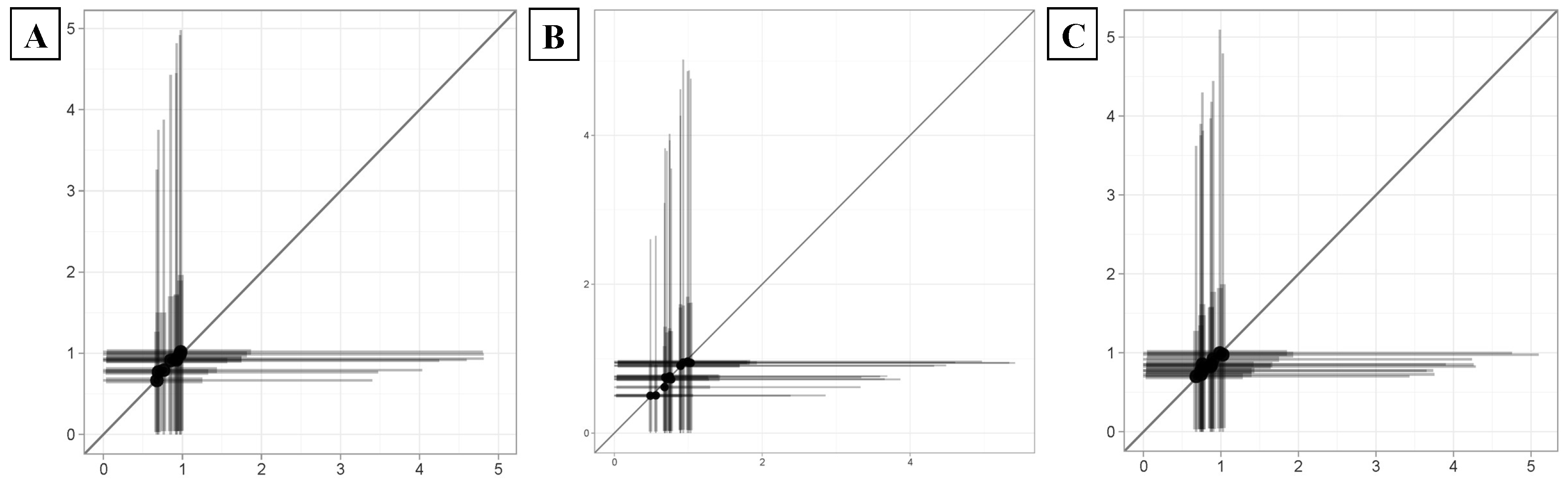


**Footnote:** NMA, network meta-analysis. A: 4 weeks; B: 8 weeks; C: 12 weeks. The horizontal coordinate represents residual deviance of consistency model and the vertical coordinate represents residual deviance of inconsistency model. All points lie roughly on the line of equality, indicating that there is no evidence for inconsistency.

Figure S5. The “dev-dev” plots of random-effects consistency and inconsistency models for NMA of adverse event rate


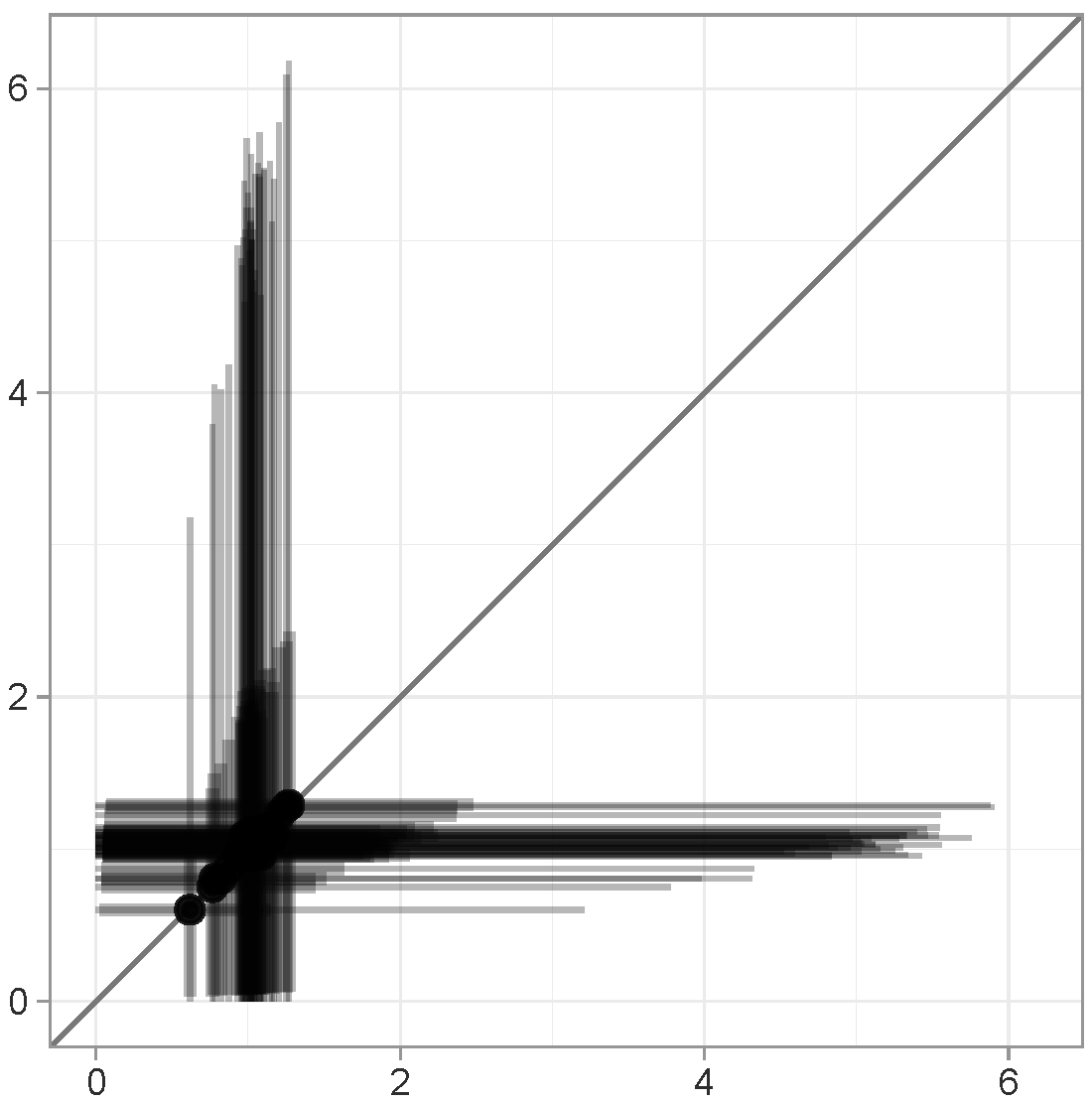


**Footnote:** NMA, network meta-analysis. The horizontal coordinate represents residual deviance of consistency model and the vertical coordinate represents residual deviance of inconsistency model. All points lie roughly on the line of equality, indicating that there is no evidence for inconsistency.

Figure S6. Network diagram of comparison of headache intensity

**
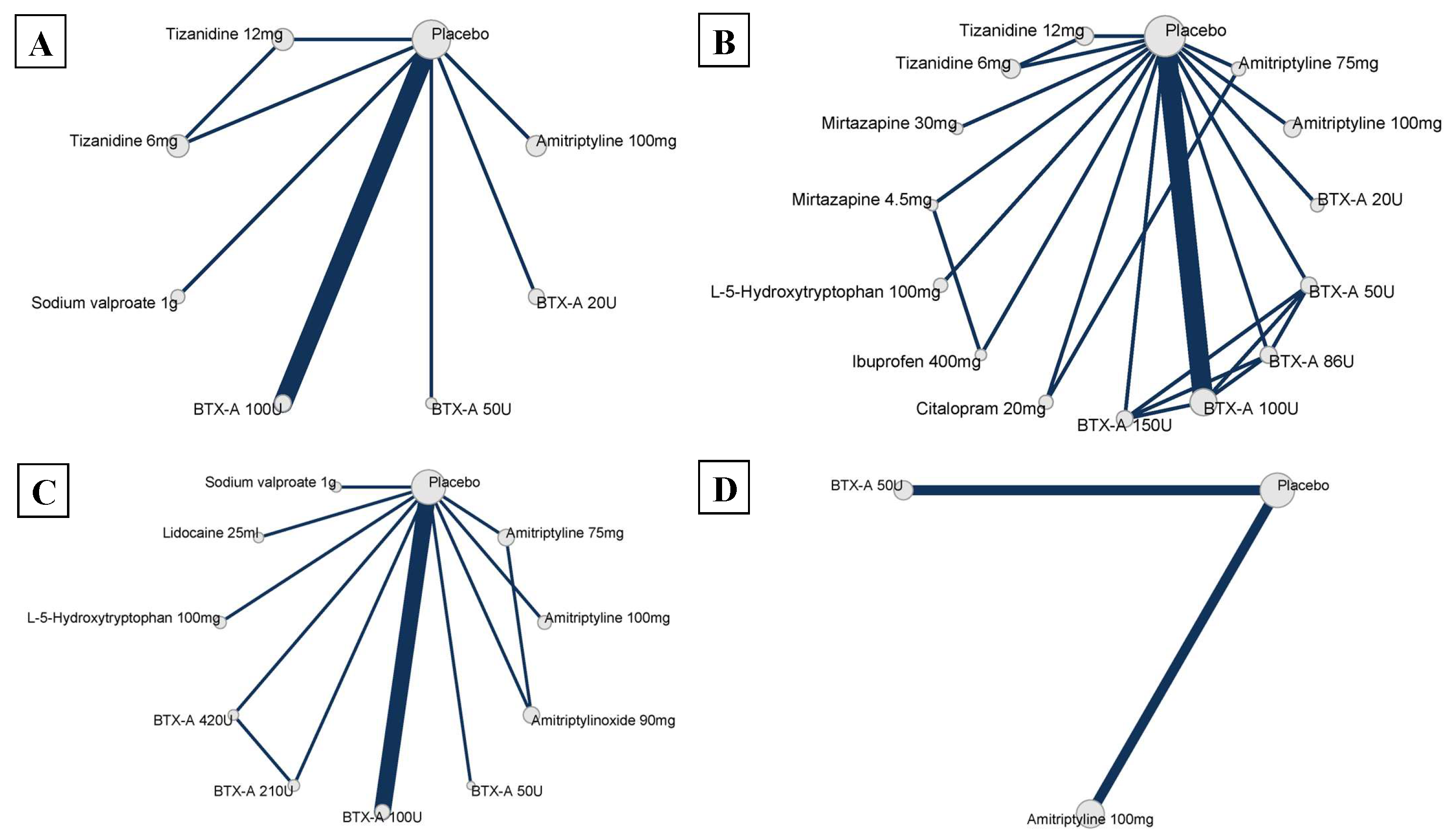
**

**Footnote:** A: 4 weeks; B: 8 weeks; C: 12 weeks; D: 24 weeks. The size of the grey nodes represents the number of included participants, and the thickness of the line represents the number of included studies.

Figure S7. Effect size of pharmacological interventions vs. Placebo of headache intensity


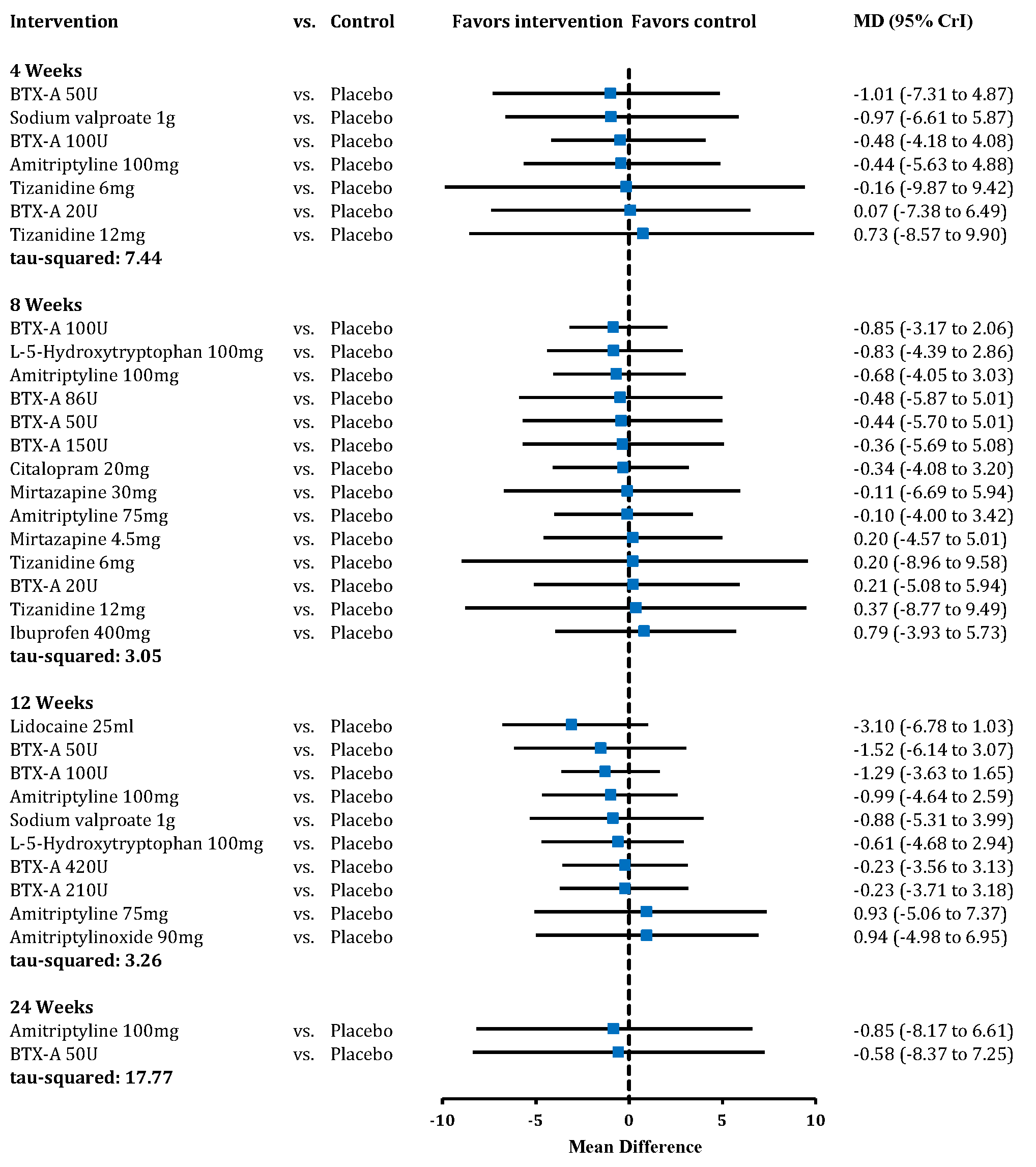


Figure S8. Network diagram of comparison of headache duration

**
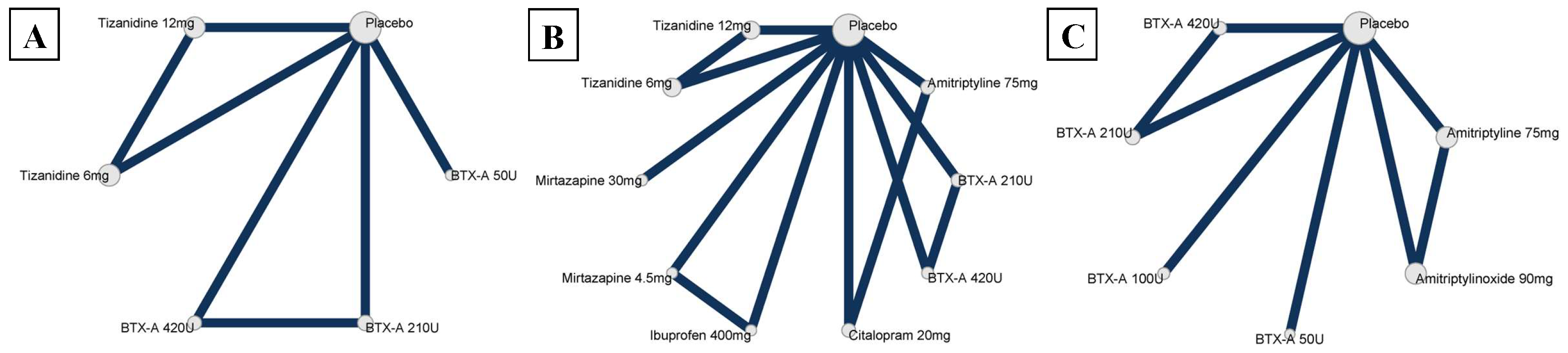
**

**Footnote:** A: 4 weeks; B: 8 weeks; C: 12 weeks. The size of the grey nodes represents the number of included participants, and the thickness of the line represents the number of included studies.

Figure S9. Effect size of pharmacological interventions vs. Placebo of headache duration


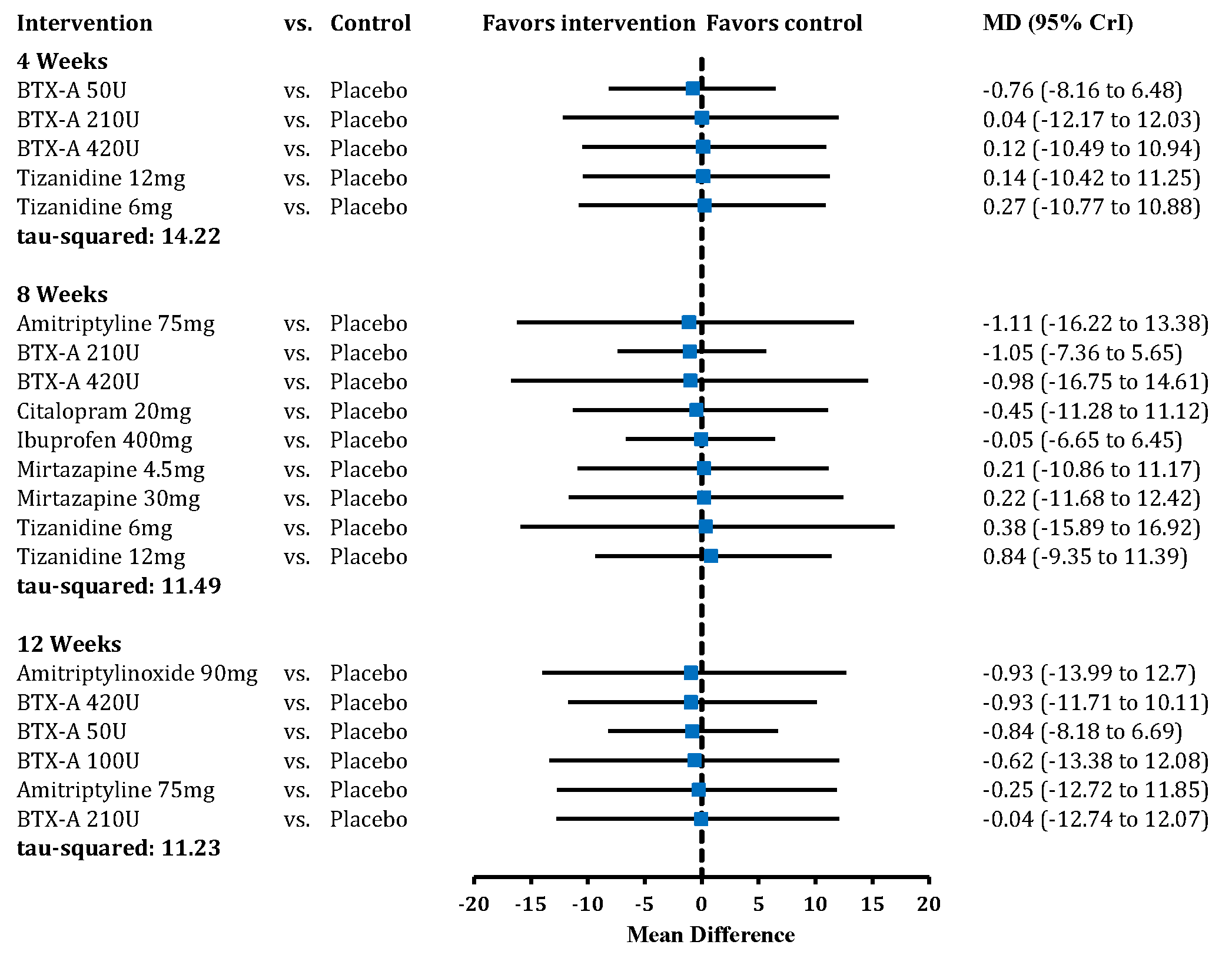


**Footnote:** BTX-A, botulinum toxin type-A; MD, mean difference; CrI, credible interval.

Figure S10. Network diagram of comparison of adverse event rate

**
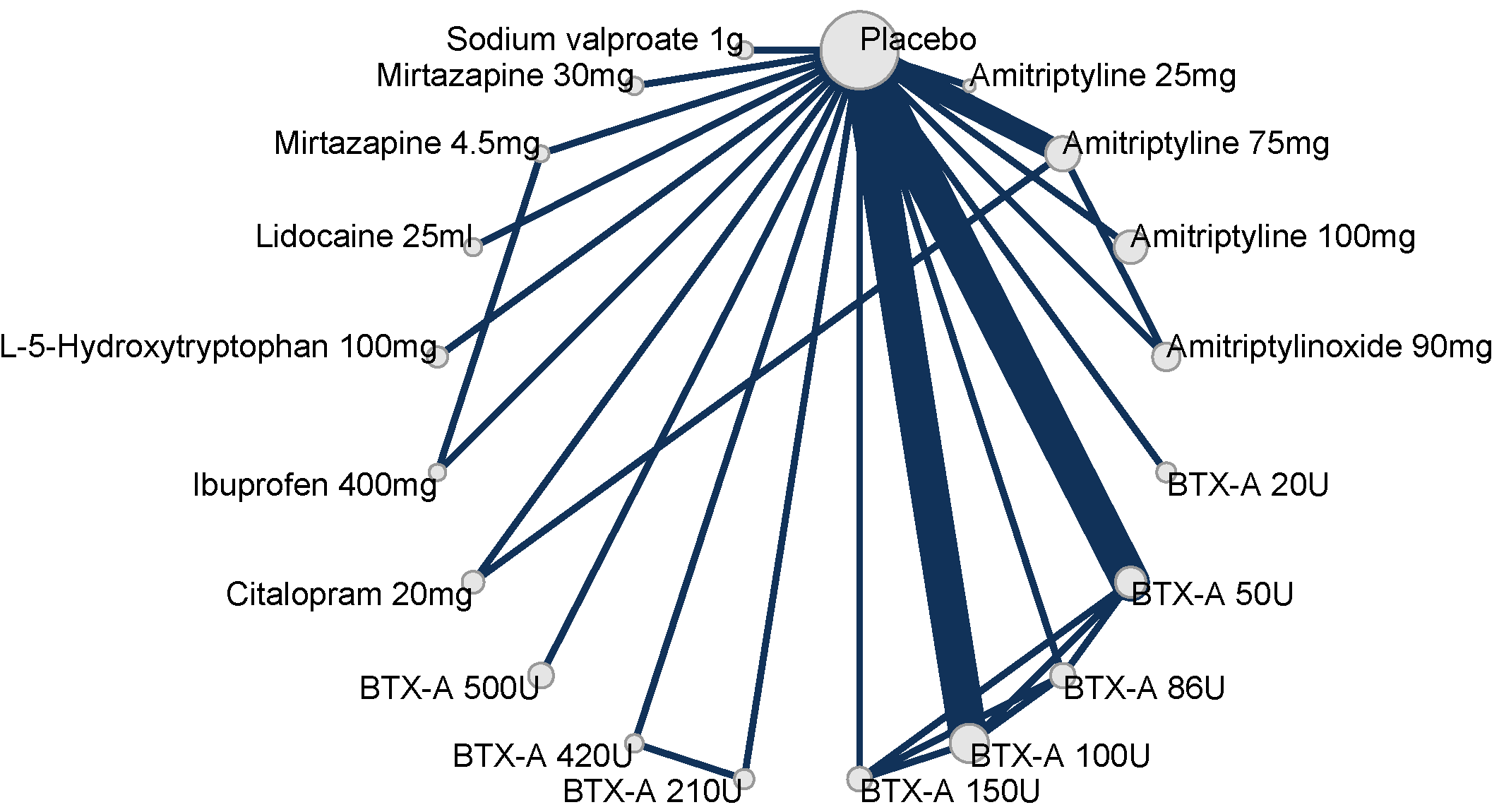
**

**Footnote:** The size of the grey nodes represents the number of included participants, and the thickness of the line represents the number of included studies.

Figure S11. Effect size of pharmacological interventions vs. placebo of adverse event rate


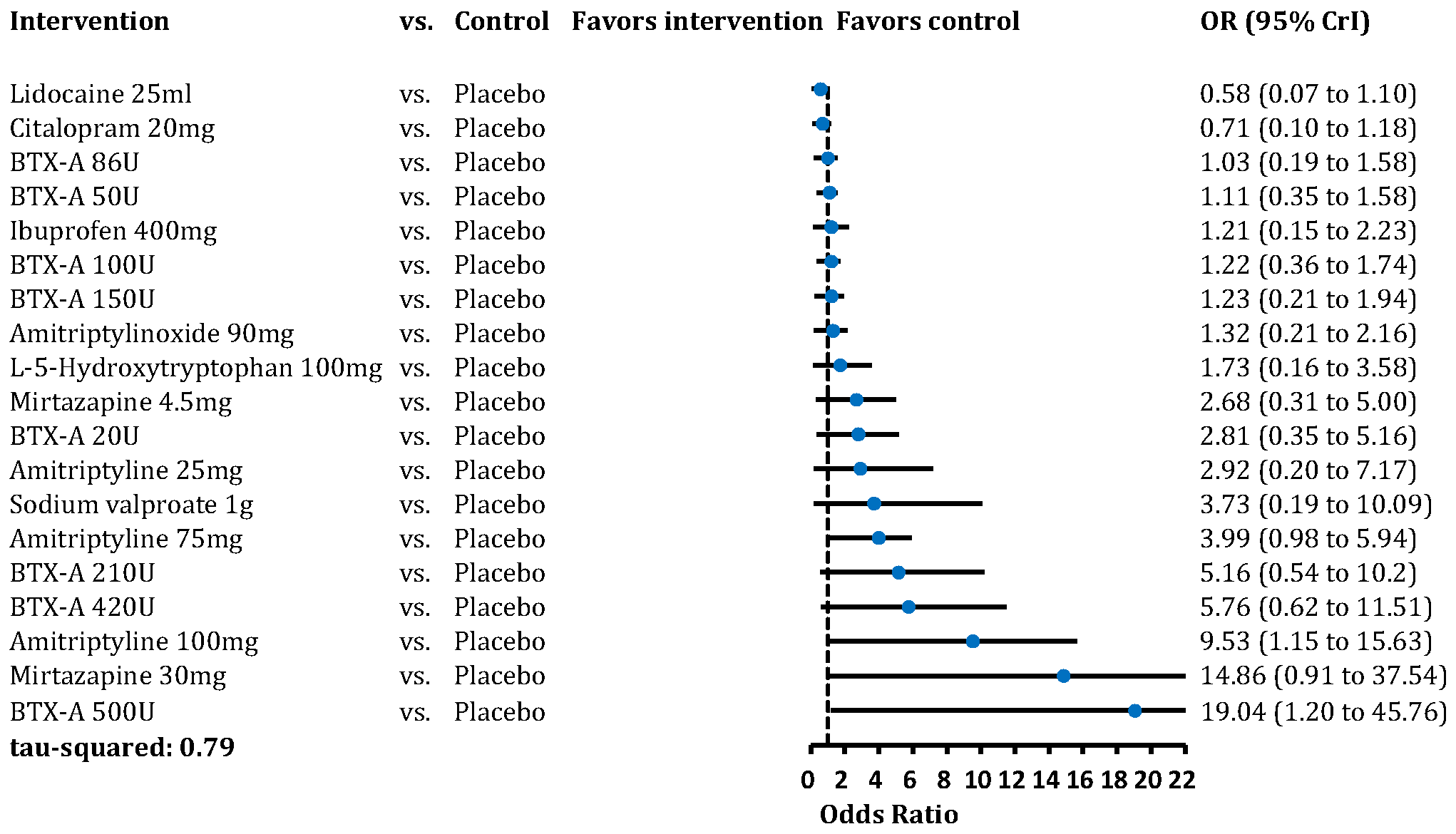


**Footnote:** BTX-A, botulinum toxin type-A; OR, odds ratio; CrI, credible interval.

Figure S12. Sensitivity analysis of headache days per month after excluding RCTs at high risk of bias


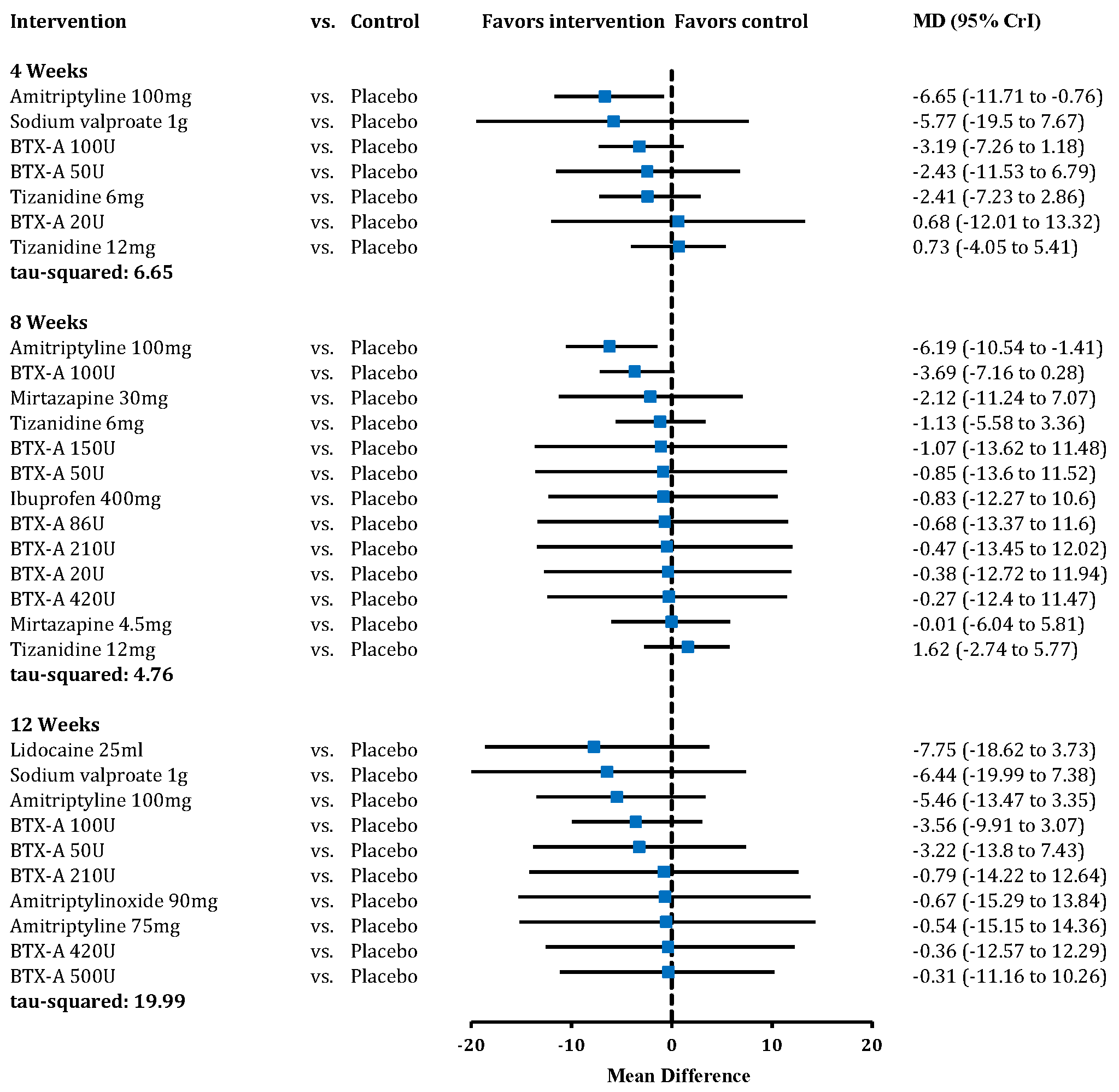


**Footnote:** BTX-A, botulinum toxin type-A; MD, mean difference; CrI, credible interval.

Figure S13. Sensitivity analysis of headache days per month after excluding RCTs less than 50 participants


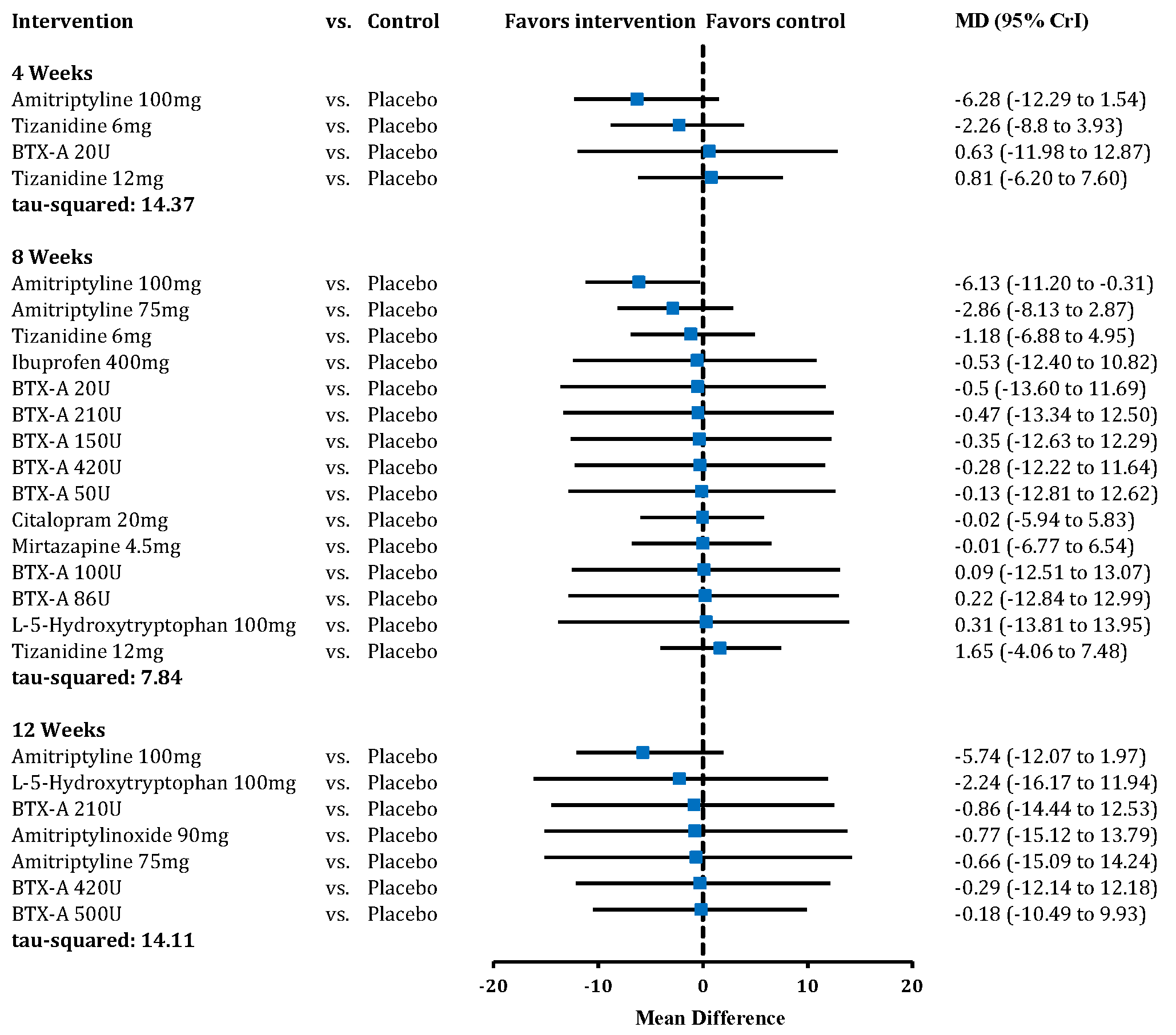


**Footnote:** BTX-A, botulinum toxin type-A; MD, mean difference; CrI, credible interval.

Figure S14. Sensitivity analysis of headache days per month using a frequentist approach


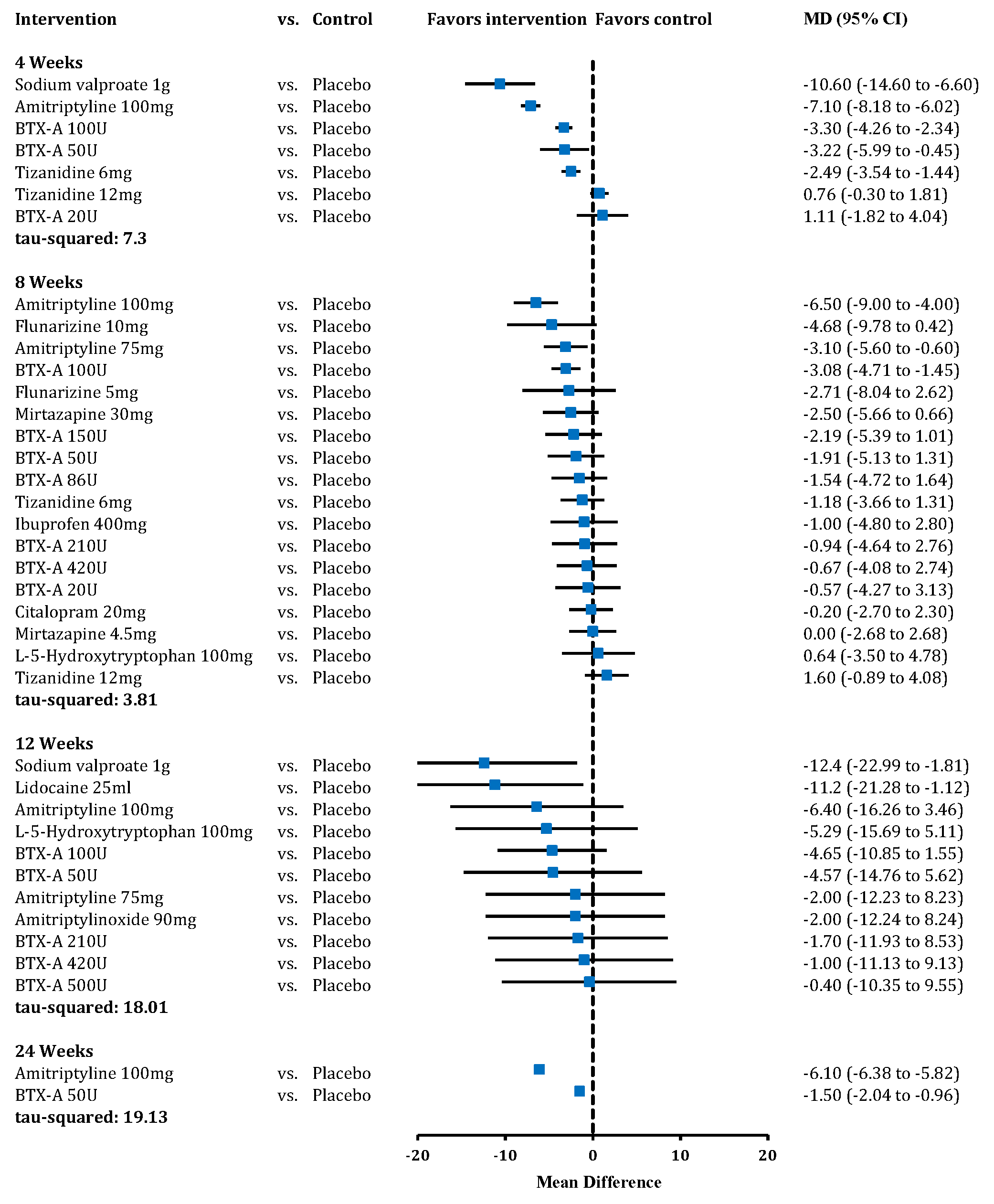


**Footnote:** BTX-A, botulinum toxin type-A; MD, mean difference; CrI, credible interval.

Figure S15. Exploratory analysis of headache days per month by combining data from episodic and chronic tension-type headache


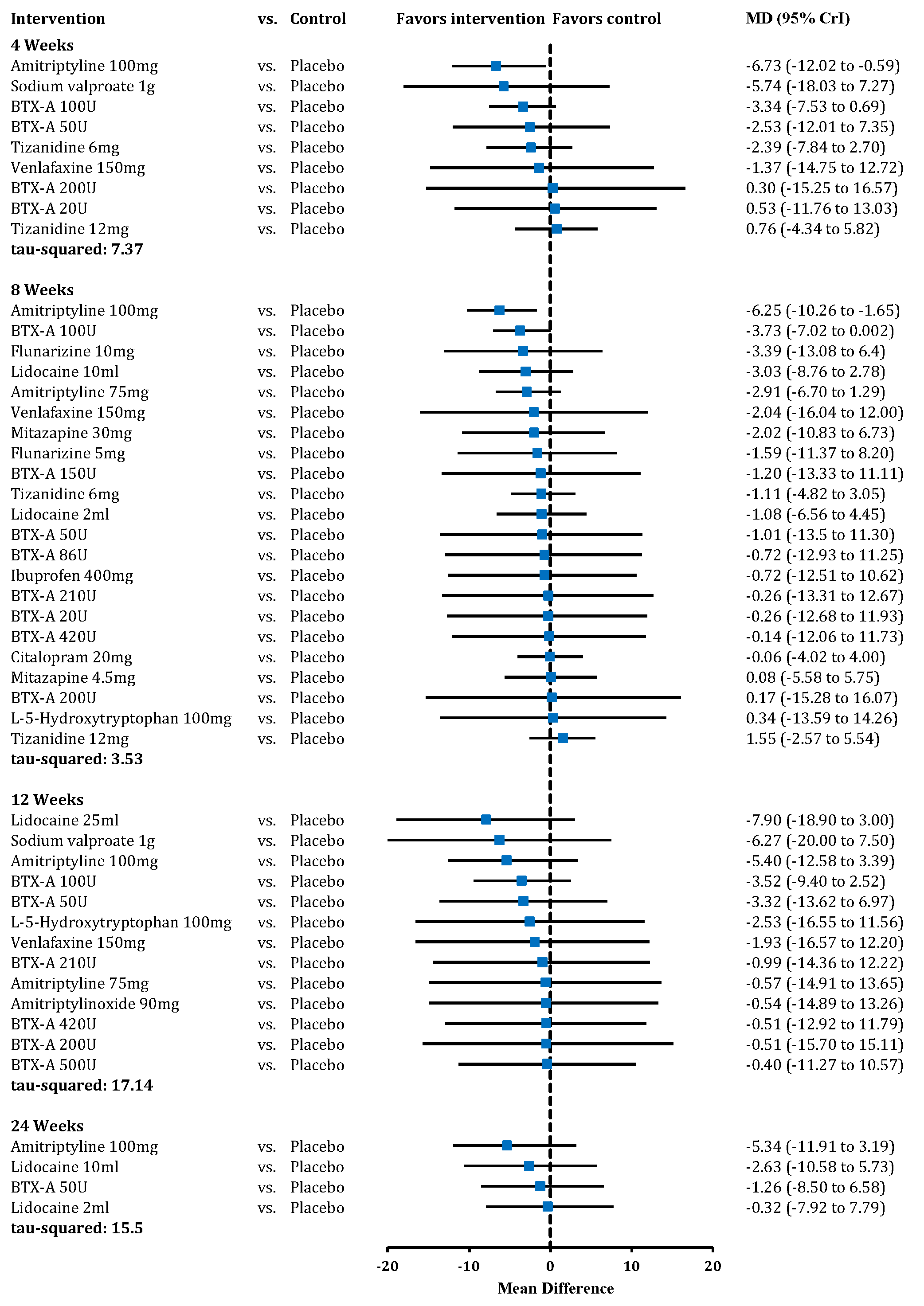


**Footnote:** BTX-A, botulinum toxin type-A; MD, mean difference; CrI, credible interval.
